# Millimeter-scale vertical partitioning of nitrogen cycling in hypersaline mats reveals prominence of genes encoding multi-heme and prismane proteins

**DOI:** 10.1038/s41396-021-01161-z

**Published:** 2021-12-03

**Authors:** P. Maza-Márquez, M. D. Lee, A. M. Detweiler, B. M. Bebout

**Affiliations:** 1 Exobiology Branch, NASA Ames Research Center, Moffett Field, CA USA; 2 Bay Area Environmental Research Institute, Moffett Field, CA USA; 3 Blue Marble Space Institute of Science, Seattle, WA USA; 4 Chan Zuckerberg Biohub, San Francisco, CA USA

**Keywords:** Microbial ecology, Population dynamics, Population genetics, Metagenomics

## Abstract

Microbial mats are modern analogues of the first ecosystems on the Earth. As extant representatives of microbial communities where free oxygen may have first been available on a changing planet, they offer an ecosystem within which to study the evolution of biogeochemical cycles requiring and inhibited by oxygen. Here, we report the distribution of genes involved in nitrogen metabolism across a vertical oxygen gradient at 1 mm resolution in a microbial mat using quantitative PCR (qPCR), retro-transcribed qPCR (RT-qPCR) and metagenome sequencing. Vertical patterns in the presence and expression of nitrogen cycling genes, corresponding to oxygen requiring and non-oxygen requiring nitrogen metabolism, could be seen across gradients of dissolved oxygen and ammonium. Metagenome analysis revealed that genes annotated as hydroxylamine dehydrogenase (proper enzyme designation EC 1.7.2.6, *hao*) and hydroxylamine reductase (*hcp*) were the most abundant nitrogen metabolism genes in the mat. The recovered *hao* genes encode hydroxylamine dehydrogenase EC 1.7.2.6 (HAO) proteins lacking the tyrosine residue present in aerobic ammonia oxidizing bacteria (AOB). Phylogenetic analysis confirmed that those proteins were more closely related to ɛHao protein present in *Campylobacterota* lineages (previously known as *Epsilonproteobacteria*) rather than oxidative HAO of AOB. The presence of *hao* sequences related with ɛHao protein, as well as numerous *hcp* genes encoding a prismane protein, suggest the presence of a nitrogen cycling pathway previously described in *Nautilia profundicola* as ancestral to the most commonly studied present day nitrogen cycling pathways.

## Introduction

Microbial mats, modern analogues of some of the most ancient ecosystems on Earth [1], are often found in environments of high salinity, desiccation, cold, limited nutrients, and high UV exposure [2–5]. As ancient ecosystems, microbial mats played a key role in the evolution of the Earth’s atmosphere, likely responsible for the majority of the oxygen that changed the early atmosphere and oceans from anoxic to oxic at the time of the Great Oxidation Event [6–8]. There is evidence from a number of sources of localized higher oxygen concentrations in an otherwise anoxic ocean before the advent of the modern-day oxygen containing atmosphere [9–12]. Because microbial mats experience both highly oxic and highly reducing conditions on a diel cycle, due to the production of oxygen by phototrophy during the day and high rates of respiration at night [13], these communities may have been the first to experience regularly oxic conditions in a predominantly anoxic ocean on Earth. It has been suggested that modern microbial mats may be models for microbial mat-based oxygen oases in the Archaean [14]. Whether or not microbial mats can be conclusively demonstrated to be the sources of oxygen in these oxygen oases, they are certainly promising communities within which to explore early lineages of oxygen-requiring metabolisms and microorganisms [15]. The nitrogen cycle, with both oxygen-requiring and non-oxygen requiring transformations of nitrogen, could have evolved in such an environment.

Laminated hypersaline microbial mats growing in salterns managed for the production of salt near Guerrero Negro, Baja California Sur, México (GN) are some of the best studied and most diverse microbial mat systems known [16–18]. They are characterized by high rates of metabolic processes carried out by cyanobacteria, phototrophic and chemotrophic sulfur-oxidizing bacteria, sulfate-reducing bacteria, and methanogens, among others [19–21]. Transformations of the nitrogen cycle including N_2_-fixation, nitrification, denitrification, anaerobic ammonium oxidation (anammox), and dissimilatory nitrate reduction were recently quantified in Guerrero Negro microbial mats [22]. Nitrogen necessary for growth of the mat is primarily understood to be obtained through nitrogen fixation, uptake of inorganic and organic sources of nitrogen from the overlying water, and through recycling of previously produced biomass [22, 23].

Here, we examine a set of functional genes catalyzing nitrogen transformations and their expression across a millimeter-scale depth gradient in the Guerrero Negro microbial mats using qPCR/RTqPCR and shotgun metagenome sequencing. The overall aim of this study was to provide a comprehensive view of the partitioning of nitrogen cycling across a depth gradient including oxic and anoxic zones.

## Material and methods

### Microbial mat samples collection

**Fig. 1:**
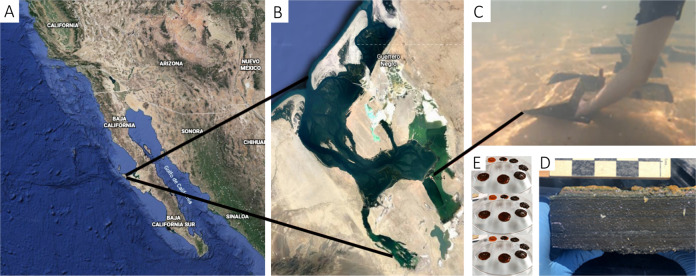
Microbial mats used for this study. Location of study sites in Guerrero Negro, Baja California, México, photo property of Google Earth (**A**). Exportadora de Sal S.A (ESSA) sampling location, Area 4, photo property of Google Earth (**B**). Microbial mat samples collection in Area 4 (**C**). Hypersaline microbial mat at the time of collection, scale at top is in centimeters, photo credit: José Q. García Maldonado (**D**). Samples were collected at 0900 h on June 16th, 2019 from Concentration Area 4. At the time of collection, the temperature of the 1 m water column above the mat was 24.4 °C. Field water measurements included salinity (125 ppt), ammonium (0.12 µM) and oxygen concentrations (7 mg/l), and pH (8.3). Nitrate concentrations were below the detection limit. Mat sections cut into 20 cm × 25 cm × 5 cm blocks. Seven 1-mm thick layers from each of three replicate cores were performed (**E**).

Microbial mats were collected from hypersaline ponds Concentration Area 4 in Exportadora de Sal S.A (ESSA), Guerrero Negro, Baja California Sur, México (27°41′15.1”N 113°54’52.1”W). Mat samples (Fig. 1) were collected and transported to NASA Ames Research Center (Moffett Field, CA) as previously described [24, 25]. Three replicate mat cores (1 cm in diameter) were obtained from one of the mats on July 3, 2019, flash frozen in liquid nitrogen, and preserved at −80 °C until nucleic extraction (three days later). Frozen mats cores were sliced at one-millimeter intervals using sterile scalpels (Fig. 1).

### Oxygen and porewater nutrient profiles

A glass microelectrode (Unisense, Aarhus, Denmark) was positioned in the mat using a laptop-controlled positioning system and data acquisition software. The oxygen concentration of the air-saturated standard was determined using tables provided by Unisense A/S (Aarhus, Denmark) and based on Garcia and Gordon [26]. Ammonium, orthophosphate, and nitrate were quantified using colorimetric methods adapted from those published by refs. [27, 28].

### DNA and RNA extraction

DNA and RNA were extracted from 1-mm slices using DNeasy and RNeasy PowerBiofilm Kit, (Qiagen, Venlo, Netherlands), according to the manufacturer’s user guide. Genomic DNA was checked for quality (A260/A280) and quantity (A260) using a nanophotometer (Implen, GmbH, München, Germany) as well as a fluorometer (Qubit, Invitrogen, Carlsbad, NM, USA) and verified by electrophoresis in agarose gel. The extracted RNA was treated for DNA contamination with TURBO DNA-free Kit (Ambion Inc., Austin, TX, USA). cDNA was synthesized from 400 ng of RNA extract using SuperScript III reverse transcriptase (Thermo Fisher Scientific). The quantity of extracted RNA was determined with a fluorometer (Qubit, Invitrogen, Carlsbad, NM, USA). Nucleic acids extracted from three replicate cores of each of seven 1-mm thick layers were used for qPCR and RT-qPCR assays, while three replicate slices of the uppermost four 1-mm first layers were pooled for metagenome library.

### qPCR and RT-qPCR amplification

Total Bacteria and Archaea were quantified using diagnostic 16 S rRNA genes. Genes involved in the nitrogen cycle were also targeted. Nitrogen fixation was assessed by quantifying copies of *nifH*, which are present in all nitrogen fixing organisms. Genes involved in the transformation of ammonium to nitrite, and subsequently to nitrate (the process of nitrification) were quantified. For the first step in this process, a gene involved in ammonia oxidation and present in ammonia-oxidizing Bacteria and Archaea was quantified separately using the primers Bacterial-*amoA* for Bacteria and Archaeal-*amoA* for Archaea. The second step of the process of nitrification, catalyzed by nitrite oxidizing bacteria, was quantified using primers for the gene nitrite oxidoreductase (*nxrB*) specific for members of the genus *Nitrospira*, the most widespread nitrite oxidizer known and are here referred to as *Nitrospira-nxrB*. Denitrification was assayed by quantifying both the gene for nitrous oxide reductase (*nosZ*) and nitrite reductase (*nirS*). The presence of all organisms known to perform anaerobic ammonium oxidation (anammox), the Planctomycetes, were estimated by quantification of their diagnostic 16 S rRNA genes, as previously described [29]; herein labeled as Planctomycetes-16S rRNA genes (anammox proxy). Gene and transcript copies were quantified in each mat slice by qPCR and RT-qPCR. Quantifications were performed using an Eco Real-Time PCR System (Illumina Inc., San Diego CA, USA). The primer pairs and annealing conditions utilized are presented in Table S1. Quantitative amplifications were performed as described previously [25]. Since the quantification of genes by qPCR is known to be influenced by the method used for nucleic acid extractions [30], gene copy numbers were normalized both by mass of mat and by mass of nucleic acid.

### Metagenome sequencing

Metagenome sequencing and library preparation were performed at Molecular Research (MR DNA, Texas, USA, http://www.mrdna.org/contact.html). The libraries were constructed using 50 ng DNA with Nextera DNA Flex library preparation kit (Illumina) according the manufacturer’s instruction. The samples were fragmented and the adapters were added. Metagenome sequencing of the libraries (pooled and diluted to 0.6 nM) was achieved using the NovaSeq 6000 platform (2 × 150 cycles; Illumina). Metagenome sequence data from the 4 depths are available through NCBI at BioProject PRJNA688760. Details of metagenome data processing are described in Supplementary Text 1, and annotated code is documented at our Open-Science Foundation [31] site: https://osf.io/9kwn3/wiki

### Statistical analyses

Differences in ratios of cDNA/DNA were compared for statistical significance using the IBM SPSS Statistics package (v.19, SPSS INC., IBM, USA). The normality of the data was checked using Shapiro–Wilk’s test. Kruskal–Wallis and Conover-Iman non-parametric tests (*p* < 0.05 significance level) were used to search for differences between the samples, since the Shapiro–Wilk test indicated that the majority of the data sets did not fit the normal distribution. The Primer software (PRIMER-E v.6.0, Plymouth, UK) was selected to analyze cDNA/DNA ratios of nitrogen gene derived from the quantifications by qPCR/RT-qPCR. The vectors representing the abiotic variables were overlaid over the MDS plots, according to Spearman rank coefficient.

## Results

### Porewater concentrations of dissolved oxygen and nutrients

The sampling location and appearance of the microbial mats used in this study in cross section are shown in Fig. 1. Profound changes in dissolved oxygen concentration were observed over the diel cycle because of high rates of oxygenic photosynthesis in the daytime and oxygen-requiring respiration at night (Table 1). Briefly, Layer 1 was characterized by oxygen concentration fluctuations in the range of 200–800 µM. Layers 2 and 3 ranged from 0–1200 µM and 0–200 µM, respectively. Mat Layer 4 (3–4 mm below the surface) may contain some dissolved oxygen near noon on days when there is high solar irradiance but stays anoxic for most hours of most days. Layers 5–7 (4–7 mm from the surface) remain anoxic.

**Table 1:** Oxygen concentrations throughout the first 4 mm of the mat measured at 100 µm resolution using microsensors, measured on 22 August, 2019.

Layer (mm)	O_2_ (µM)	NH_4_^+^ (µM)	NO_3_^−^ (µM)	PO_4_^−^ (µM)
1 (0–1)	200–800	34.1 ± 12.8	33.2 ± 10.9	5.5 ± 1.3
2 (1–2)	0–1200	58.6 ± 27.5	30.3 ± 2.8	2.6 ± 0.3
3 (2–3)	0–200	110.8 ± 62.4	30.5 ± 3.7	3.1 ± 0.7
4 (3–4)	only detectable under very high light	109.2 ± 52.7	30.5 ± 3.7	3.1 ± 0.7
5 (4–5)	nd	116.5 ± 69.0	30.6 ± 10.9	2.7 ± 1.2
6 (5–6)	nd	115.6 ± 38.4	30.6 ± 10.9	2.7 ± 1.2
7 (6–7)	nd	124.0 ± 34.3	26.05 ± 5.2	4.6 ± 0.7

Ranges are based on oxygen measurements taken over the course of the day at approximately 15 min intervals. Ammonium, orthophosphate, and nitrate were measured at 1 mm resolution in porewater samples extracted from the mat using a centrifugation technique. Microbial mat was cored using a corer made from a 50 cc syringe and sectioned at 1 mm intervals with razor blades. Mat slices were placed into perforated cups made from 15 mL Nalgene bottles which were then placed into 50 mL centrifuge tubes. Upon centrifugation at 2500 rpm for 5 min at room temperature, porewater left the mat, went through a glass fiber filter (Whatman GF/C) located at the bottom of the perforated cup, and collected in the bottom of the centrifuge tubes. Typically, 100 to 500 µL of porewater was recovered from each 1 mm mat slice. Average values ± standard deviation of the concentration of ammonium (NH_4_^+^), nitrate (NO_3_^−^) and phosphate (PO_4_^−^) in a hypersaline microbial mat at different depths. The depths shown are Layer 1 (0–1 mm from surface), Layer 2 (1–2 mm from surface), Layer 3 (2–3 mm from surface), Layer 4 (3–4 mm from surface), Layer 5 (4–5 mm from surface), Layer 6 (5–6 mm from surface), Layer 7 (6–7 mm from surface); nd: not detectable.

Concentrations of ammonium (Table 1) reveal a pattern of increasing concentration with depth (34–124 µM) through the layers examined here. Nitrate concentrations ranged between 26–33 µM, with low variation across depths. The concentration of phosphate ranged between 3–6 µM, with the highest concentration detected in Layer 1 (0–1 mm from surface) at 5.5 µM.

### Analysis of genes and transcripts in mat layers by qPCR and RT-qPCR

**Fig. 2:**
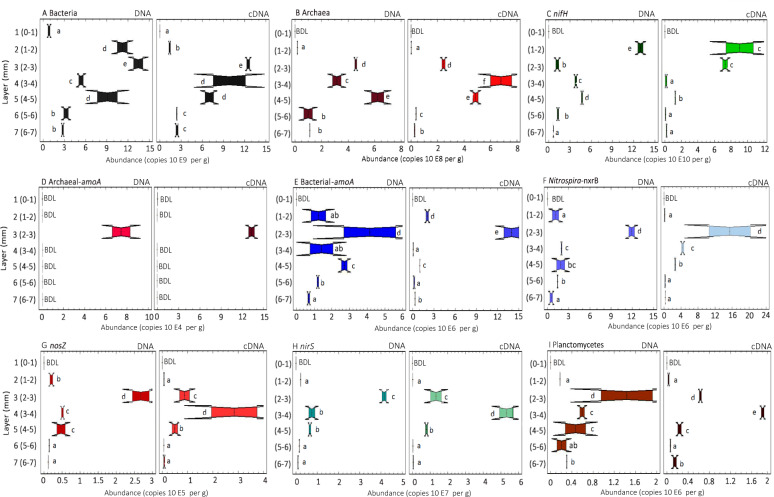
Vertical patterns in the abundance (DNA) and expression (cDNA) of Bacterial and Archaeal ribosomal and nitrogen cycling genes. Number of copies of DNA and cDNA genes recovered for Bacteria (**A**), Archaea (**B**), *nifH* (**C**), Archaeal-*amoA* (**D**), Bacterial-*amoA* (**E**), *Nitrospira-nxrB* (**F**), *nosZ* (**G**), *nirS* (**H**) and Planctomycetes-16S rRNA gene marker (anammox proxy) (**I**), per g of microbial mat, quantified by qPCR and RT-qPCR in hypersaline microbial mat profiles from different depths. *P*-values from Kruskal–Wallis test are overlain on each, and different letters indicate significantly different values for the given gene based on a Conover-Iman test *p*-value of < = 0.05.

Gene-copy number ranges for both DNA and cDNA across all layers for all genes examined are summarized as follows: Bacteria, 10^4^−10^10^ per g mat and 10^1^−10^5^, per ng nucleic acid; Archaea, 10^6^−10^8^ and 10^2^−10^4^; *nifH*, 10^8^−10^11^ and 10^4^−10^7^; Archaeal-*amoA*, 10^4^−10^5^ and 2–3; Bacterial-*amoA*, 10^4^−10^7^ and 3–335; *Nitrospira-nxrB*, 10^5^−10^7^ and 27–372; *nosZ*, 10^3^−10^5^ and 2–10; *nirS*, 10^5^−10^7^ and 33–1941; Planctomycetes-16S rRNA gene and cDNA of transcripts, 10^4^−10^6^ and 6–66 (Fig. 2, S1).

The number of DNA and cDNA copies of 16 S rRNA genes attributable to Bacteria and to Archaea exhibited vertical changes across depths examined (Fig. 2A, B and S1). The highest copy numbers of 16 S rRNA gene markers for Bacteria per g of mat were detected in Layers 2 and 3 (1–3 mm from surface), while the highest transcript numbers per g were detected in Layers 3 and 4 (2–4 mm from surface). The highest number of 16 S rRNA gene and 16 S transcript markers for Archaea were detected in Layers 3–5 (2–5 mm from surface), being one order of magnitude higher than the other layers (Fig. 2B).

The abundance of *nifH* genes was highest in Layer 2 (1–2 mm from surface). The greatest number of transcribed *nifH* genes were found in Layers 2 and 3 in the data normalized by mass of mat, while highest number of transcripts were detected in Layer 2 when the data was normalized by ng of cDNA (Fig. 2C; Fig. S1C).

Archaeal *amoA* genes and transcripts were only detected in Layer 3 (2–3 mm from surface) (Fig. 2D). Layer 3 (2–3 mm) had the highest numbers of gene copies and transcripts of Archaeal-*amoA*, Bacterial-*amoA* and *Nitrospira-nxrB* (DNA: 7.5 × 10^4^, 4.2 × 10^6^ and 1.2 × 10^7^; cDNA: 1.3 × 10^5^, 1.4 × 10^7^ and 1.6 × 10^7^ copies per g mat, respectively; Fig. 2D–F). Moreover, the transcript number of Bacterial-*amoA* and *Nitrospira-nxrB* were one order of magnitude higher in Layer 3 than in the other layers.

The number of DNA and cDNA copies of *nosZ* and *nirS* genes across all layers ranged from 6.9 × 10^3^ to 2.8 × 10^5^; 1.9 × 10^5^ to 5.3 × 10^7^; copies per g mat, respectively (Fig. 2G, H). For both genes, the highest number of DNA copies was detected in Layer 3 (2.7 × 10^5^; 4.2 × 10^7^; copies per g mat, *nosZ* and *nirS* genes, respectively), while the highest number of transcripts were found in Layer 4 (3-4 mm): 2.8 × 10^5^; 5.3 × 10^7^ copies per g mat, *nosZ* and *nirS* genes, respectively.

The abundance and transcripts numbers of Planctomycetes-16S rRNA genes in the different layers varied in a range from 3.23 × 10^4^ to 1.81 × 10^6^ per g mat (or 6.23 to 40.5 copies per ng of nucleic acid). The highest numbers of copies were detected in Layer 3 (2–3 mm from surface), while the greatest number of transcripts were detected in Layer 4 (Fig. 2I).

### cDNA/DNA ratios across depths in the mat

**Fig. 3:**
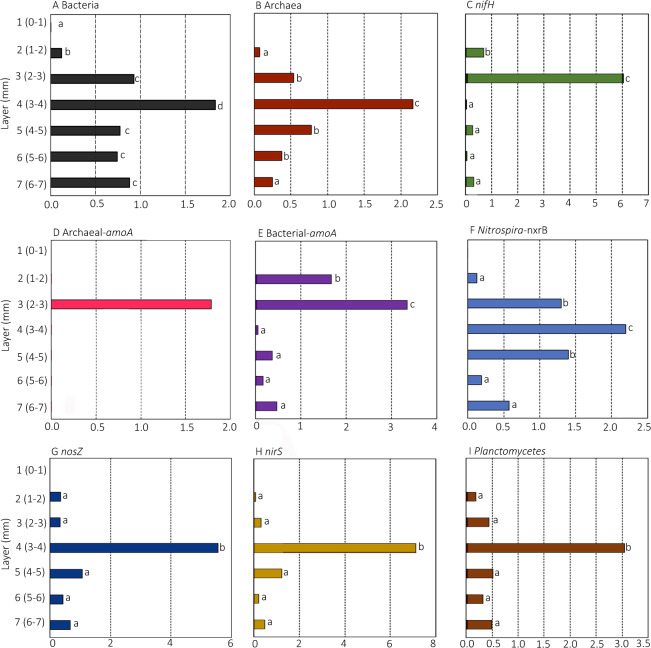
Vertical patterns in the expression of Bacterial and Archaeal ribosomal and nitrogen cycling genes. Ratios of cDNA/DNA for Bacterial (**A**), Archaea (**B**), *nifH* (**C**), Archaeal-*amoA* (**D**), Bacterial-*amoA* (**E**), *Nitrospira-nxrB* (**F**), *nosZ* (**G**), *nirS* (**H**) and Planctomycetes-16S rRNA gene marker (anammox proxy) (**I**), per g of microbial mat, quantified by qPCR and RT-qPCR in hypersaline microbial mat profiles from different depths. Different letters indicate significantly different values based on layers for each marker gene based on Conover-Iman tests with a *p*-value of < = 0.05.

The cDNA/DNA ratio (a proxy for gene transcription) for all genes quantified by qPCR is shown in Fig. 3. The cDNA/DNA ratio of all genes exhibited significant changes across depths. For all genes examined, the ratio was maximal in either Layer 3 or Layer 4. The cDNA/DNA ratios for the domain-specific genes for Bacteria and Archaea had a more even distribution across depths than the nitrogen cycling genes, with the exception of *Nitrospira-nxrB*, which was also more evenly distributed. The genes *nifH*, Archaeal*-amoA*, and Bacterial*-amoA*, all had a maximal cDNA/DNA ratio in Layer 3. The amplicons *Nitrospira-nxrB*, *nosZ*, *nirS*, and Planctomycetes 16 S rRNA genes all had a maximal cDNA/DNA ratio in Layer 4. The overall magnitude of the cDNA/DNA ratio was also different between the genes, with *nirS* and *nosZ* both exhibiting the highest ratios (over 5 and 7, respectively) and the greatest differences between the peak layer (Layer 4) and the other layers in the mat.

### NMDS ordination analysis of genes

**Fig. 4:**
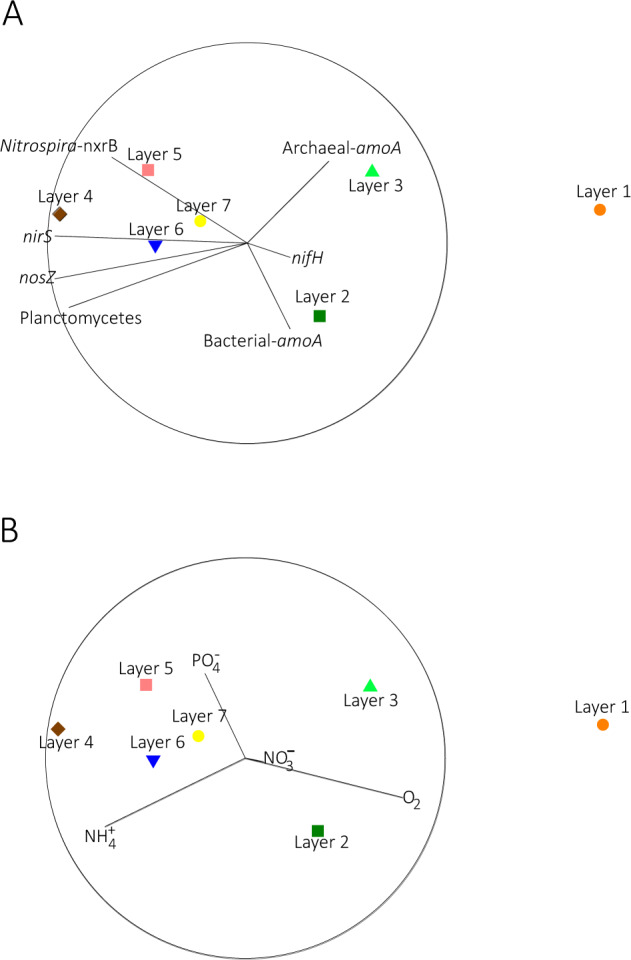
Non-metric multidimensional scaling (NMDS) plots of quantification of all nitrogen genes across all layers examined in this study. Genes associated with the following nitrogen transformations were examined: nitrogen fixation (*nifH*), nitrification (Bacterial-*amoA*, Archaeal-*amoA*, *Nitrospira-nxrB*), denitrification (*nosZ, nirS*) and Planctomycetes-16S rRNA gene marker (anammox proxy). The biotic data was standardized, and a sample resemblance matrix was generated using Bray-Curtis coefficient of similarity. In order to analyze the influence of abiotic variables (porewater nutrient and oxygen concentration) on the patterns of the biotic data, monotonic correlations of the abiotic variables were performed. In the plots, the distance between the samples’ points reflects their relative similarity, according to Bray-Curtis similarity matrices based on cDNA/DNA ratios of nitrogen genes examined. The vectors in panel **A** represent the cDNA/DNA ratios of nitrogen gene examined. In panel **B**, the vectors represent the environmental variables.

NMDS ordination analysis of the cDNA/DNA ratio of genes involved in nitrogen transformations was conducted to examine the relationship of these ratios to each other and to the dissolved oxygen and nutrient concentrations measured in these layers (Fig. 4). *nifH* and *amoA* genes were positively correlated (*r* ≥ 0.60) and moreover, the highest ratios of *nifH* and *amoA* genes were detected in Layer 2 and 3 (2–3 mm from surface). *Nitrospira-nxrB* displayed a strong positive correlation with *nirS* (*r* = 0.96), *nosZ* (*r* = 0.78), and Planctomycetes-16S rRNA genes (*r* = 0.96). The highest cDNA/DNA ratios of denitrifying genes (*nirS* and *nosZ*) were found in Layer 4 and strong positive correlation was detected between them (*r* > 0.8). Furthermore, the ratio of Planctomycetes-16S rRNA genes (Fig. 4A) was positively correlated with denitrifying organisms’ genes (*r* > 0.8, Table 2).

**Table 2:** (A) Spearman correlations coefficient (*r*) between the ratios of cDNA/DNA of nitrogen fixation (*nifH*), nitrification (Bacterial-*amoA*, Archaeal-*amoA*, *Nitrospira-nxrB*), denitrification (*nosZ, nirS*) and Planctomycetes-16S rRNA gene marker (anammox proxy) and oxygen, ammonium, nitrate and phosphate concentrations. (B) Spearman correlation *p*-value.

A	*nifH*	Archaeal-*amoA*	Bacterial-*amoA*	*Nitrospira-nxrB*	Planctomycetes-16S	*nirS*	*nosZ*
*nifH*	1	0.61	1	0.11	0.04	0.04	−0.14
Archaeal-*amoA*	0.61	1	0.61	0.20	0	0	−0.41
Bacterial-*amoA*	1	0.61	1	0.11	0.03	0.04	−0.14
*Nitrospira-nxrB*	0.11	0.20	0.11	1	0.96	0.96	0.78
Planctomycetes-16S	0.03	0	0.03	0.96	1	1	0.89
*nirS*	0.03	0	0.03	0.96	1	1	0.89
*nosZ*	−0.14	−0.41	−0.14	0.78	0.89	0.89	1
O_2_ (µM)	0.24	0.22	0.24	−0.68	−0.77	−0.77	−0.79
NH_4_^+^ (µM)	−0.05	−0.31	−0.05	0.38	0.50	0.50	0.67
NO_3_^−^(µM)	−0.75	−0.52	−0.75	−0.38	−0.24	−0.24	−0.09
PO_4_^−^(µM)	−0.90	−0.52	−0.90	0.05	0.13	0.13	0.20

B							
*nifH*		0.13	0.00	0.79	0.93	0.93	0.73
Archaeal-*amoA*	0.13		0.13	0.62	1.00	1.00	0.32
Bacterial-*amoA*	0.00	0.13		0.79	0.93	0.93	0.73
*Nitrospira-nxrB*	0.79	0.62	0.79		0.02	0.02	0.05
Planctomycetes-16S	0.93	1.00	0.93	0.02		0.00	0.03
*nirS*	0.93	1.00	0.93	0.02	0.00		0.03
*nosZ*	0.73	0.32	0.73	0.05	0.03	0.03	
O_2_ (µM)	0.61	0.63	0.61	0.99	0.04	0.04	0.04
NH_4_^+^ (µM)	0.91	0.50	0.91	0.40	0.23	0.25	0.11
NO_3_^−^(µM)	0.05	0.23	0.05	0.39	0.61	0.61	0.84
PO_4_^−^(µM)	0.00	0.23	0.00	0.9	0.78	0.78	0.66

*nifH*, Bacterial-*amoA* and Archaeal-*amoA* were positively correlated with oxygen concentration (*r* ≥ 0.22, Table 2), while *Nitrospira-nxrB* was negatively correlated with oxygen (*r* = −0.68, Table 2). Denitrification genes (*nosZ*, *nirS*) and Planctomycetes-16S rRNA genes were all positively correlated with ammonium (*r* ≥ 0.5) and orthophosphate (*r* ≥ 0.13) and negatively correlated with oxygen (*r* > −0.70).

### Metagenome analysis of nitrogen cycling

**Fig. 5:**
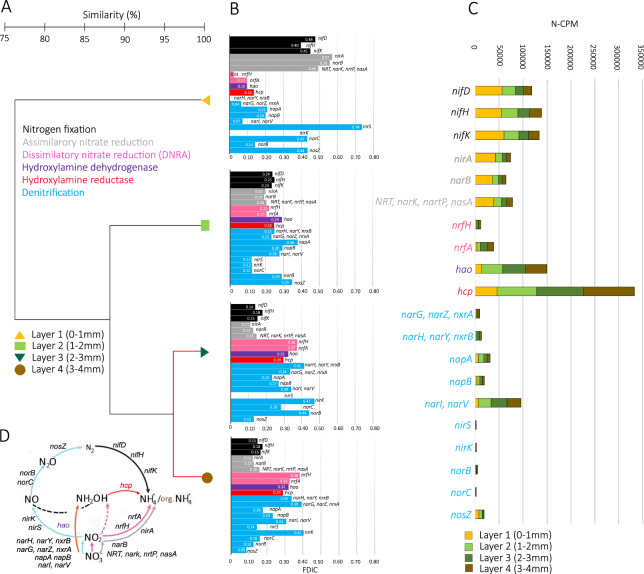
Functional nitrogen gene distribution based on metagenome analysis. **A** Cluster analysis illustrating the similarity of normalized coverages of all recovered nitrogen metabolism genes across the uppers 4 layers examined [(Layer 1 (0–1 mm from surface), Layer 2 (1–2 mm from surface), Layer 3 (2–3 mm from surface), Layer 4 (3–4 mm from surface)]. Red lines show non-significant differences, according to SIMPROF analysis (*p* > 0.05). **B** The bar plots show the genes of the metabolic pathways in the nitrogen cycle identified in the mat, according metagenome analysis, with relative coverage of each nitrogen cycling gene across depths examined (Fraction of Depth Integrated Coverage, FDIC). 355 unique genes were recovered from KEGG’s Nitrogen Metabolism pathway: 60 annotated as involved in nitrogen fixation, 15 in assimilatory nitrate reduction, 38 in dissimilatory nitrate reduction to ammonia (DNRA), 52 in hydroxylamine dehydrogenase EC 1.7.2.6, 121 in hydroxylamine reductase, 69 in denitrification pathway. **C** Values of Nitrogen-focused Coverage per Million (N-CPM). The following enzymes perform nitrogen transformation in the mat: nitrogenase molybdenum-iron protein alpha chain (*nifD*), nitrogenase iron protein *NifH*, nitrogenase molybdenum-iron protein beta chain (*nifK*), hydroxylamine dehydrogenase EC 1.7.2.6 (*hao*), hydroxylamine reductase (*hcp*), nitrate reductase/nitrite oxidoreductase, alpha subunit (*narG, narZ, nxrA*), nitrate reductase/nitrite oxidoreductase, beta subunit (*narH, narY, nxrB*), nitrate reductase (cytochrome) (*napA*), nitrate reductase (cytochrome), electron transfer subunit (*napB*), nitrite reductase (NO-forming) / hydroxylamine reductase (*nirS*), nitrogenase molybdenum-iron protein beta chain (*nirK*), nitric oxide reductase subunit B (*norB*), nitric oxide reductase subunit C (*norC*), nitrous-oxide reductase (*nosZ*), nitrate reductase gamma subunit (*narI, narV*), cytochrome c nitrite reductase small subunit (*nrfH*), nitrite reductase (cytochrome c-552) (*nrfA*), ferredoxin-nitrite reductase (*nirA*), ferredoxin-nitrate reductase (*narB*), MFS transporter, NNP family, nitrate/nitrite transporter (*NRT, nark, nrtP, nasA*). **D** Nitrogen cycling genes recovered in this study and the transformation that they catalyze.

A total number of 922 324 genes were identified; 1305 of these genes were annotated with KOs that are part of KEGG’s Nitrogen Metabolism pathway (Table S2, S3). A dendrogram based on Bray-Curtis similarities of normalized coverages of all recovered nitrogen metabolism genes is shown in Fig. 5A. Overall, the similarity between the layers was >75%. According to SIMPROF analysis, there was a significant difference in the N-related gene coverages (based on an alpha value of 0.05) between Layers 1-Layer 2, Layer 3, and Layer 4 (*p* = 0.001) and Layer 2-Layer 3, and Layer 4 (*p* = 0.001), but not between Layers 3 and Layer 4 (*p* = 1), where the similarity was >90%.

The nitrogen fixation pathway was identified with *nifD, nifH*, and *nifK* genes (Fig. 5B, C, Table S4). Of the 60 genes detected in this metabolic pathway 17 genes were annotated as *nifD*, 22 genes as *nifH*, and 21 genes as *nifK*. The normalized coverage of these genes showed a decreasing trend with depth. Layer 1 was characterized by the highest values of Nitrogen-focused coverage per million (N-CPM, see Supplementary Text 1) of *nifD, nifH*, and *nifK* genes: 56264.7, 54934.2 and 60059.2, respectively. On average, the three genes involved in nitrogen fixation, *nifD, nifH*, and *nifK*, decreased with depth, (2.7-fold from Layer 1 to Layer 4, with a nearly 2-fold difference solely between Layer 1 and Layer 2).

Genes involved in nitrite and nitrate assimilation, annotated as *nirA* and *narB* which code for ferredoxin nitrite and nitrate reductases respectively, were 3 times as abundant in Layer 1 than Layer 2, but decreased less markedly from Layer 2 to Layers 3 and 4.

Genes for dissimilatory nitrite reduction (*nrfA, and nrfH*) were 4 and 16 times more abundant in Layer 4 than Layer 1. Similarly, the nitrate/nitrite regulator protein genes *narl* and *narV* displayed a nearly inverse pattern, with Layer 1 having the least proportion of genes, a large increase from Layer 1 to Layer 2, and additional increases from Layer 2 to Layers 3 and Layer 4 (Fig. 5B, C, Table S4).

Genes associated with nitrification were very poorly represented in the metagenome. No genes associated with ammonia oxidation (*amoA*) were detected. Genes associated with nitrite oxidation (*nxrA, nxrB*) that were detected are so closely related to denitrifier genes (*narG, narZ, narH, narY*) as to be annotated with the same KEGG KO models (K00370 representing *narG*, *narZ*, *nxrA*; and K00371 representing *narH*, *narY*, *nxrB*).

The following genes involved in denitrification were detected: *napA, napB, narG, narZ, narH, narY, narI, narV, nirK, nirS, norB, norC*, and *nosZ* (Fig. 5B, C). The nitrate reduction metabolic pathway was represented by 4 genes encoding the nitrate reductase-nitrite oxidoreductase-alpha subunit (*narG, narZ, nxrA* genes), 6 genes encoding the nitrate reductase-nitrite oxidoreductase-beta subunit (*narH, narY, nxrB* genes), 31 genes encoding the nitrate reductase gamma subunit (*narI, narV*), 5 genes encoding the nitrate reductase -cytochrome electron transfer subunit (*napB*) and 7 genes encoding the nitrate reductase -cytochrome (*napA*) (Table S4). The N-CPM of nitrate reductase increased with depth, but with a similar proportion of those genes in Layers 3 and 4. With respect to nitrite reductase (*nirk* and *nirS* genes, 2 and 1 genes, respectively), no *nirK* genes were detected in Layer 1, where the highest N-CPM of *nirS* was recovered (Fig. 5B). In contrast, Layer 3 had no detected *nirS* and the highest N-CPM of *nirK*. Regarding nitric oxide reductase (*norB* and *norC* genes, 6 and 1 genes, respectively), the highest normalized coverage of *norB* was detected in Layer 3, while highest for *norC* was in Layer 1. Finally, *nosZ* (6 genes) was detected in all the layers, steadily decreasing in normalized coverage from the top layer to the deepest (Fig. 5B, C; Table S4).

DNRA metabolism was represented by *nrfA* (26 genes) and *nrfH* (12 genes), and by *narI, narV* (31). Layer 1 was characterized by the lowest normalized coverage of *narI, narV, nrfA*, and *nrfH* genes (6880.2, 3724.6, and 284.6 N-CPM, respectively), while Layer 3 was characterized by the greatest coverage of *narI, narV*, *nrfA*, and *nrfH* genes (32760.5, 14417.9 and 4504.1, respectively; Fig. 5B, C; Table S4).

Genes for hydroxylamine dehydrogenase EC 1.7.2.6 and hydroxylamine reductase (*hao* and *hcp*, respectively) were the most abundant nitrogen metabolism genes in the mat: *hao* having a cumulative N-CPM of ~150000 and *hcp* having a cumulative N-CPM of nearly 350,000 across the 4 depths (Fig. 5C). Both genes increased in abundance with depth; *hcp* increased two-fold between Layer 1 and Layer 2, and more gradually in Layer 3 and Layer 4. *Hao* exhibited a three-fold increase in relative abundance from Layer 1 to Layer 2 and remained relatively constant through Layer 3 and Layer 4 (Fig. 5B, C; Table S4).

## Discussion

Microbial mats are visibly stratified to the eye, and are characterized by steep vertical gradients in oxygen, H_2_S, pH, and light [13, 32]. Dynamic changes in electron donors and acceptors to diverse microbial metabolisms are likely associated with the physiochemical gradients within this physically and chemically stratified structure. To the best of our knowledge, the present study is the first to examine the abundance and activity of genes involved in the nitrogen cycle at 1 mm resolution in microbial mats. Previous high resolution studies of microbial mat community composition recognized the importance of a dynamic and extreme gradient in redox, but necessarily inferred function from the phylogenetic affinities of the organisms sequenced [18, 33, 34].

### The depth distribution of nitrogen gene copy numbers detected by qPCR-RTqPCR

Layer 1 (0–1 mm from surface) was characterized by the lowest number of copies and transcripts of all the targeted groups. Moreover, the numbers of Archaea, *nifH*, Bacterial-*amoA*, Archaeal-*amoA*, *Nitrospira*-*nxrB*, Planctomycetes-16S rRNA transcripts (anammox proxy), *nosZ*, and *nirS* all ranged below of the limit of detection at this first layer (Fig. 2). This is perhaps not surprising as Layer 1 was characterized microscopically as consisting primarily of diatoms and exopolysaccharides and was the least cohesive and least dense of all layers examined. Moreover, the low numbers of microorganisms detected in Layer 1 is consistent with a recent study based on quantification of eukaryotic microorganisms in the same microbial mat [35].

Microbial mats are well known for their capacity to fix nitrogen and there have been many studies of the distribution of the genes responsible. The greatest amount of DNA and cDNA copies of *nifH* were detected in Layer 2 (1–2 mm from surface) and numbers of copies generally decrease in deeper layers (Fig. 2C). The highest cDNA/DNA ratios of *nifH* genes were detected 2–3 mm from surface, above the oxic/anoxic chemocline. This distribution of copies and transcripts of *nifH* is perhaps surprising given the well-documented sensitivity of the process of nitrogen fixation to oxygen. However, the presence of maximal rates of nitrogen fixation in the surface layers of microbial mats has been reported previously [36–38]. Certainly, it is the case that the large amounts of energy derived from sunlight are necessary for nitrogen fixation [23]. High light, low dissolved ammonium concentrations, and a greater metabolic requirement for the production of new biomass are all characteristics of the surface layers of microbial mats. Higher ammonium concentrations in deeper layers of the mat may be explained by ammonification, which provides an additional source of fixed nitrogen to microorganisms removed from the surface layers and/or lacking the capacity for diazotrophy, as has been previously speculated [39], and more recently experimentally verified [22].

A number of studies of the process of nitrogen fixation in microbial mats have reported a strong diel pattern of acetylene reduction, a proxy for nitrogen fixation, with peak rates occurring at night [23, 36, 37, 40–43]. The samples processed for molecular sequencing were collected at noon, when the cDNA/DNA ratio for *nifH* should have been relatively lower. It may be the case that many more transcripts of *nifH* would be detected in a sample of the mat taken at night, and this possibility is presently being investigated. It seems likely, however, that the peak cDNA/DNA ratio of *nifH* will always occur in the oxic portion of the mat. It is also important to highlight the fact that the *nifH* primers used in this study, as with all primers, have several limitations [44] and that there is known extensive post-transcriptional regulation of *nifH* [45]. It has also been shown that nitrogenase activity can be measured even if no expression of the gene is detected [46].

The dissolved oxygen that is necessary for the process of nitrification would be only minimally available below Layer 3 and then only during the daytime portion of the diel cycle. The expression-ratio (as denoted by cDNA/DNA ratio) of Bacteria-*amoA* and Archaea-*amoA* can be seen to be highest in Layer 3. Nitrifying microorganisms, however, are known to be sensitive to light [47], and are also likely to be outcompeted for ammonium by photosynthetic organisms in the sunlit upper layers of the mat. The maximal expression of *Nitrospira-nxrB* was found in Layer 4 (Fig. 2F). Certainly, it is the case that difference between the peak activity and the activity through the other layers is much less pronounced for *Nitrospira-nxrB* than for any other nitrogen cycling gene examined. Spearman rank showed a negative correlation with oxygen (Table 2). Nitrite oxidizing bacteria (NOB) are a phylogenetically diverse and metabolically versatile group, including mixotrophic metabolisms [48] and complex lifestyles beyond the nitrogen cycle [49]. The oxygen necessary in the oxidation of nitrite to nitrate is taken from water and not from dioxygen gas, making the reaction independent of the oxygen status of the environment [50], and members of this diverse functional group have been detected in oxygen minimum zones [51].

Genes associated with nitrogen transformations not requiring, and/or inhibited by oxygen (*nosZ*, *nirS* and Planctomycetes-16S rRNA genes) are maximal in Layer 4. Negative correlations were detected between *nosZ* and *nirS* gene ratios and oxygen concentration (Table 2). Low oxygen concentrations in Layers 4 and below (even during periods of daytime photosynthetic activity) would likely be conducive for the use of nitrate as an alternative electron acceptor. Layer 4 is characterized by oxygen tensions, which are only occasionally (and only under extremely high light), rise above zero (Table 1).

### The vertical distribution of nitrogen cycling as represented by metagenome sequencing

Genes involved in nitrogen fixation and in assimilatory nitrate reduction were most abundant in Layer 1. The presence of *nifD*, *nifH* and *nifK* is consistent with the high abundance of cyanobacteria in this layer, as seen in both the gene-level and read-based taxonomies (Fig. S3) and the generally-recognized large quantitative role of cyanobacteria in nitrogen fixation in microbial mats. The highest N-CPM of assimilatory nitrite and nitrate reduction (*nirA* and *narB* genes) were also detected in Layer 1 (Fig. 5), and were also contributed by genes taxonomically classified as Cyanobacteria (Table S5).

Genes associated with the process of nitrification (*amo*, *hao* and *nxr*) were not well-represented in the metagenomes. The gene *amoA*, which is one of at least 3 known subunits present in the complex AMO enzyme needed for catalyze the oxidation of ammonia to hydroxylamine [52] was not detected in the metagenome, despite it having been amplified by qPCR. To investigate if this could be due to a failure of any copies of *amoA* successfully assembling, or not being successfully annotated, we recruited each samples’ metagenome reads to a reference database of *amoA* genes. This revealed that any known *amoA* genes are indeed present only in low relative abundance, as the only recruitment was for 3 read-pairs in Layer 2 (~96% identical to *Nitrosospira*), explaining their lack of detection in the metagenome data. Representatives of known obligate ammonia oxidizers (*Nitrosomonas* and *Nitrosospira*) were, however, detected in the read-based taxonomy from the metagenome (Table S6). Nitrite oxidation was potentially represented in the metagenome by nitrite oxidoreductase (*nxrA, nxrB)* genes. However, *nxrA* and *nxrB* are closely related to denitrifying genes (*narG, narZ*, and *narH, narY*), and are annotated with the same KO models (K00370 representing *narG*, *narZ*, *nxrA*; and K00371 representing *narH*, *narY*, *nxrB*). In both cases, it is not certain there is enough information in their sequences alone to distinguish them functionally (Figs. S4, S5). Similar results were reported in a study based on metagenome assembled genomes from ammonium-rich geothermal groundwater microbial mat [53]. In a recent study based on stable isotope tracer measurements in the same microbial mat studied here, nitrification metabolism was detected only at very low rates [22]. Previous discussions of reasons for low rates of nitrification in microbial mat communities have noted sensitivities of nitrifying organisms to light and salinity [47, 54].

It was notable that the genes encoding hydroxylamine dehydrogenase EC 1.7.2.6 and hydroxylamine reductase (*hao* and *hcp*, respectively) were the most abundant N cycling genes in the mat. Both *hao* and *hcp* genes are related with hydroxylamine metabolism. Hydroxylamine is a potent toxin, and its presence in microbial communities has been previously noted as a factor driving the evolution of enzymatic function and mechanisms for its detoxification [55, 56]. With respect to sources, hydroxylamine is well-characterized as an intermediate in the oxic nitrogen metabolic pathway of the aerobic ammonia oxidizing bacteria (AOB) [52]. We are aware only a few inconclusive reports of other microbial sources of hydroxylamine, e.g., denitrification of nitrate with generation of hydroxylamine [57–59]. Hydroxylamine is also involved in anaerobic ammonium oxidation, and eight of the *hcp* genes detected in the metagenome were annotated as belonging to Planctomycetes, the phylum of Bacteria containing all known anaerobic ammonium oxidizing organisms. However, while qPCR assays did detect both the presence and expression of Planctomycetes-16S rRNA gene marker in the mat, with a sharp peak located in Layer 4, no sequences of the enzyme hydrazine oxidoreductase (*hzo*) and hydrazine synthase (*hzs*), normally present in ammonium oxidizing organisms, were detected; 277 genes related to microorganisms described as anammox bacteria such as Candidatus *Scalindua* sp, Candidatus *Scalindua rubra*, Candidatus *Jettenia ecosi*, Candidatus *Jettenia caeni*, Candidatus *Kuenenia stuttgartiensis*, Candidatus *Brocadiae bacterium*, Candidatus *Brocadia* sp, were identified in the taxonomy from the metagenome (Table S7). This, coupled with a lack of anammox activity detected in ^15^N-labeling experiments [22], is consistent with the possibility that there may be Planctomycetes in these Guerrero Negro mats that are not performing anammox. No *hzs* genes were detected in another hypersaline mat from Australia in a study employing a metagenome-assembled genomes approach [34].

**Fig. 6:**
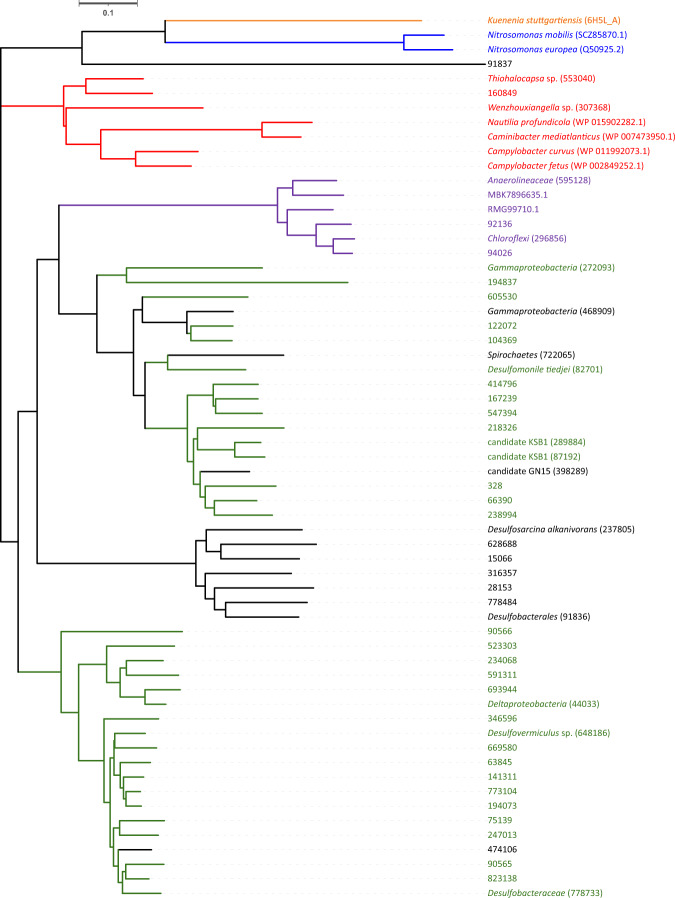
Phylogenetic tree based on different hydroxylamine dehydrogenase (HAO) proteins. Phylogenetic tree constructed with the 52 HAO proteins sequences detected in our study, HAO from AOB *Nitrosomonas europaea* and *Nitrosomonas mobilis* (blue color), HAO from anammox bacteria *Kuenenia stuttgartiensis* (orange color) and HAO from *Campylobacterota*: *Campylobacter fecus, Campylobacter curvus, Caminibacter mediatlanticus* and *Nautilia profundicola* (red color). HAO from nitrate reductase (purple color).

Important clues as to the function of *hao* and *hcp* genes were provided by an examination of the proteins they are predicted to encode. Turning first to *hao*, the top Pfam annotation for all 52 of our *hao* (K10535) genes was PF13447 (Table S8), a multiheme cytochrome for which the majority of sequences underlying the Pfam model were annotated as hydroxylamine dehydrogenase EC 1.7.2.6 (HAO). HAO reductases may be differentiated from oxidative HAOs by one structural characteristic, namely a tyrosine residue which cross links the active site to the catalytic heme moiety of an adjacent subunit; the presence of this cross-linked active site heme has been hypothesized to modulate enzyme reactivity toward oxidative catalysis [60]. Conversely, the absence of this cross-link is predicted to favor reductive catalysis. In order to determine whether or not the tyrosine residue was present, the 52 HAO sequences recovered in the metagenome presented here (Table S8) were aligned with HAO from *Nitrosomonas europea* and *Nitrosomonas mobilis* (AOB), HAO from the anaerobic ammonium-oxidizing (anammox) bacterium *Kuenenia stuttgartiensis* described in the Ferousi [60] and Dietl [61], ɛHao protein (HAO subfamily) from the *Campylobacterota* [62] (previously known as *Epsilonproteobacteria*) and other microorganisms described in Hasse [63] (Fig. S6). For the correct position of residual tyrosine in HAO from *Nitrosomonas europea*, we follow the study of Klotz [56]. Without exception, the HAO EC 1.7.2.6 proteins predicted from the mat metagenome *hao* genes do not have the tyrosine residue present in HAO oxidase of AOB (Fig. S6). A tree constructed using these predicted proteins confirmed that the majority of them were not closely related with the HAO of AOB (*Nitrosomonas mobilis* and *Nitrosomonas europea*) or with the HAO of anammox *Kuenenia stuttgartiensis*, which clustered in separate clades (Fig. 6). Three of the HAO proteins identified in this study (in red color, Fig. 6), were related to ɛHAO proteins and share the same clade. ɛHAO proteins included in this tree have been described with capability of nitrite reduction [63]. Two sequences related to nitrate reductase in publicly GenBank database were included (MBK7896635.1 and RMG99710.1; in purple Fig. 6). Four of the HAO proteins identified in this study form a monophyletic clade with them (>74% of identity and 99–98% query cover on the amino acid level, Table S9). Moreover, 38 of the proteins from this study (33 in green color, 4 related to nitrate reductase activity in purple and one related to ɛHao protein in red) have two conserved amino acid that Hasse considered important in ɛHao protein. A tryptophan in the equivalent position of the key tyrosine in nitrifier HAO and a conserved methionine eight amino acids prior to the tryptophan (see the alignment in Fig. S6).

Pfam annotations supported the KO annotations of all 121 of our *hcp* (K05601) genes, annotating them all as PF03063 (prismane), for which the vast majority of sequences underlying the annotation model are annotated as hydroxylamine reductase (Table S8). The type of prismane proteins these models capture (those with hybrid-cluster Fe-S complexes) have been suggested to have a role in nitrate/nitrite respiration under anaerobic conditions [64]. *Hcp* codes for hydroxylamine reductase, a hybrid cluster or prismane protein which has been described as important in the processes of scavenging hydroxylamine and with NO detoxification [55, 56]. The presence of hydroxylamine in microbial communities has been previously noted as a factor driving the evolution of enzymatic function and mechanisms for its detoxification [55, 56].

It is unclear why the coverage of genes associated with hydroxylamine metabolism are so high in the metagenomes of the microbial mat studied here. Certainly, the ability to detoxify hydroxylamine would seem an important capability for mat microorganisms. It is also worth noting these genes may exist as many paralogs within the same organism, further suggesting they may be functionally divergent despite being closely related. For example, in the Planctomycetes *Kuenenia stuttgartiensis*, there are 10 paralogs of *hao* [65]. An intriguing possibility, explaining many of the observations reported here, is that the N cycle in the mat contains a previously described novel pathway of microbial nitrite reduction, the reverse hydroxylamine: ubiquinone reductase module (reverse-HURM) pathway [66]. Subsequent to the description from its genome, the pathway was experimentally verified in the *Nautilia profundicola* [67]. ɛHao proteins derived from the phylum Campylobacterota have been described as a “missing link” in the evolution of the multiheme cytochrome c family (MCC). Some MCCs important to nitrogen cycling include pentaheme cytochrome c nitrite reductase (NrfA), octaheme hydroxylamine dehydrogenase (HAO), and octaheme hydrazine dehydrogenase (HDH) [63]. The presence of *hao* EC 1.7.2.6 sequences related with ɛHao protein and HCP prismane protein in the metagenome may indicate that this ancestral metabolic pathway is present in the mat and would explain hydroxylamine generation in anoxic conditions rather than by a traditional HAO oxidase and *amoA* gene from AOB. HAO in this pathway would reduce nitrite (and possibly nitrate, based on copies we recovered that group with known nitrate reductases-purple in Fig. 6) to hydroxylamine which would be subsequently reduced to ammonium by HCP (Fig. 5D). A high capacity for hydroxylamine consumption was noted in cultures of *N. profundicola*, suggesting a selective pressure to keep hydroxylamine concentrations low due to mutagenic effect on cell growth [67]. In our study, *hcp* was the most abundant N cycling gene, indicating this as a possible mechanism of cell protection from mutagenic hydroxylamine in the mat. Further experiments are needed in order to corroborate this pathway or to elucidate any other roles these genes may be involved in that may be responsible for their prominence in this system.

It is not currently clear how to reconcile the notable diversity of ammonia oxidizing bacteria (AOB) and anammox bacteria with the lack of octaheme cytochrome c (OCC) proteins, hydroxylamine dehydrogenase (HAO; which oxidizes hydroxylamine to NO), and hydrazine dehydrogenase (HDH; which catalyzes the oxidation of hydrazine to N_2_) described in these microorganisms. Microbial mats, which may represent the first environments in which free oxygen was present on a reliable basis, are valuable environments for studying the evolution of biogeochemical cycling.

## Supplementary information

Supplementary material for: Millimeter-scale vertical partitioning of nitrogen cycling in hypersaline mats reveals prominence of genes encoding multi-heme and prismane proteins

Supplementary tables for: Millimeter-scale vertical partitioning of nitrogen cycling in hypersaline mats reveals prominence of genes encoding multi-heme and prismane proteins

## References

[1] Awramik SM. Ancient stromatolites and microbial mats. In: Microbial mats: Stromatolites. Cohen Y, Castenholz RW, Haivorson HO, editors. Alan R Liss, NY; 1984. p. 133–148.

[2] Walter MR, Buick R, Dunlop JSR (1980). Stromatolites 3,400–3,500 Myr old from the North Pole area, Western Australia. Nature.

[3] Cohen Y. The Solar Lake cyanobacterial mats: strategies of photosynthetic life under sulfide. In: Microbial mats: Stromatolites. Cohen Y, Castenholz RW, Haivorson HO, editors. Alan R Liss, NY; 1984. p. 133–148.

[4] Foster JS, Mobberley JM. Past, present, and future: microbial mats as models for astrobiological research. In: Microbial Mats: Modern and Ancient Microorganisms in Stratified Systems. Seckbach J, Oren A, editors. Springer, Berlin, Heidelberg; 2010. p. 563–582.

[5] Stal LJ. Microbial mats in coastal environments. In: Microbial Mats. Stal LJ, Caumetter P, editors. Springer, Berlin, Heidelberg; 1994. p. 21–32.

[6] Hayes JM. Geochemical evidence bearing on the origin of aerobiosis, a speculative hypothesis. In: Schopf JW, editor. Earth’s Earliest Biosphere: Its Origin and Evolution. Princeton University Press; 1983. p. 292–301.

[7] Schopf JW, Klein C, editors. Frontmatter. In: The Proterozoic biosphere: a multidisciplinary study. Cambridge, University Press; 1992. p. i–vi.

[8] Hoehler TM, Bebout BM, Des, Marais DJ (2001). The role of microbial mats in the production of reduced gases on the early Earth. Nature.

[9] Olson SL, Kump LR, Kasting JF (2013). Quantifying the areal extent and dissolved oxygen concentrations of Archean oxygen oases. Chem Geol.

[10] Lalonde SV, Konhauser KO (2015). Benthic perspective on Earth’s oldest evidence for oxygenic photosynthesis. PNAS.

[11] Eigenbrode JL, Freeman KH (2006). Late Archean rise of aerobic microbial ecosystems. PNAS.

[12] Kendall B, Reinhard CT, Lyons TW, Kaufman AJ, Poulton SW, Anbar AD (2010). Pervasive oxygenation along late Archaean ocean margins. Nat Geosci.

[13] Revsbech NP, Jorgensen BB, Blackburn TH, Cohen Y (1983). Microelectrode studies of the photosynthesis and O2, H2S, and pH profiles of a microbial mat. Limnol Oceanogr.

[14] Sumner DY, Hawes I, Mackey TJ, Jungblut AD, Doran PT (2015). Antarctic microbial mats: a modern analog for Archean lacustrine oxygen oases. Geology.

[15] Nisbet EG, Fowler CMR (1999). Archaean metabolic evolution of microbial mats. Proc R Soc Lond B.

[16] Ley RE, Harris JK, Wilcox J, Spear JR, Miller SR, Bebout BM (2006). Unexpected diversity and complexity of the Guerrero Negro hypersaline microbial mat. Appl Environ Microbiol.

[17] Feazel LM, Spear JR, Berger AB, Harris JK, Frank DN, Ley RE (2008). Eucaryotic diversity in a hypersaline microbial mat. Appl Environ Microbiol.

[18] Harris JK, Caporaso JG, Walker JJ, Spear JR, Gold NJ, Robertson CE (2013). Phylogenetic stratigraphy in the Guerrero Negro hypersaline microbial mat. ISME J.

[19] Canfield DE, Des Marais DJ (1993). Biogeochemical cycles of carbon, sulfur, and free oxygen in a microbial mat. Geochim Cosmochim Acta.

[20] Canfield DE, Des, Marais DJ (1991). Aerobic sulfate reduction in microbial mats. Science.

[21] Tazaz AM, Bebout BM, Kelley CA, Poole J, Chanton JP (2013). Redefining the isotopic boundaries of biogenic methane: methane from endoevaporites. Icarus.

[22] Coban O, Rasigraf O, de Jong AEE, Spott O, Bebout BM (2021). Quantifying potential N turnover rates in hypersaline microbial mats by 15N tracer techniques. Appl Environ Microbiol.

[23] Bebout BM, Fitzpatrick MW, Paerl HW (1993). Identification of the sources of energy for nitrogen fixation and physiological characterization of nitrogen-fixing members of a marine microbial mat community. Appl Environ Microbiol.

[24] Bebout BM, Carpenter SP, Des Marais DJ, Discipulo M, Embaye T, Garcia-Pichel F (2002). Long-term manipulations of intact microbial mat communities in a greenhouse collaboratory: simulating earth’s present and past field environments. Astrobiology.

[25] Coban O, Williams M, Bebout BM (2018). Mechanisms of nitrogen attenuation from seawater by two microbial mats. Water Res.

[26] Garcia HE, Gordon LI (1992). Oxygen solubility in seawater: better fitting equations. Limnol Oceanogr.

[27] Parsons TR, Maita Y, Lalli CM, editors. A manual of chemical and biological methods for seawater analysis. Oxford, NY, Pergamon Press; 1984.

[28] Ringuet S, Sassano L, Johnson ZI (2011). A suite of microplate reader-based colorimetric methods to quantify ammonium, nitrate, orthophosphate and silicate concentrations for aquatic nutrient monitoring. J Environ Monit.

[29] Humbert S, Zopfi J, Tarnawski SE (2012). Abundance of anammox bacteria in different wetland soils. Environ Microbiol Rep.

[30] Smith CJ, Osborn AM (2009). Advantages and limitations of quantitative PCR (Q-PCR)-based approaches in microbial ecology. FEMS Microbiol Ecol.

[31] Foster M, Deardorff M. Open Science Framework (OSF). J Medical Library Association. 2017: 105.

[32] Jorgensen BB, Des, Marais DJ (1990). The diffusive boundary layer of sediments: oxygen microgradients over a microbial mat. Limnol Oceanogr.

[33] Wong HL, Smith DL, Visscher P, Burns BP (2015). Niche differentiation of bacterial communities at a millimeter scale in Shark Bay microbial mats. Sci Rep.

[34] Wong HL, White RA, Visscher PT, Charlesworth JC, Vázquez-Campos X, Burns BP (2018). Disentangling the drivers of functional complexity at the metagenomic level in Shark Bay microbial mat microbiomes. ISME J.

[35] Maza-Márquez P, Lee MD, Bebout BM (2021). The abundance and diversity of fungi in a hypersaline microbial mat from Guerrero Negro, Baja California, México. J Fungi.

[36] Stal LJ, Grossberger S, Krumbein WE (1984). Nitrogen fixation associated with the cyanobacterial mat of a marine laminated microbial ecosystem. Mar Biol.

[37] Woebken D, Burow L, Prufert-Bebout L, Bebout BM, Hoehler TM, Pett-Ridge J (2012). Identification of a novel cyanobacterial group as active diazotrophs in a coastal microbial mat using NanoSIMS analysis. ISME J.

[38] Woebken D, Burow L, Behnam F, Mayali X, Schintlmeister A, Fleming ED (2015). Revisiting N_2_ fixation in Guerrero Negro intertidal microbial mats with a functional single-cell approach. ISME J.

[39] Bebout BM, Paerl HW, Bauer JE, Canfield DE, Des Marais DJ. Nitrogen cycling in microbial mat communities: the quantitative importance of N-fixation and other sources of N for primary productivity. In: Microbial Mats. NATO ASI Series (Series G: Ecological Sciences). Stal LJ, Caumette P, editors. Springer, Berlin, Heidelberg; 1994. p. 265–271.

[40] Bebout BM, Paerl HW, Crocker KM, Prufert LE (1987). Diel interactions of oxygenic photosynthesis and N_2_ fixation (acetylene reduction) in a marine microbial mat community. Appl Environ Microbiol.

[41] Omoregie EO, Crumbliss LL, Bebout BM, Zehr JP (2004). Comparison of diazotroph community structure in Lyngbya sp. and *Microcoleus chthonoplastes* dominated microbial mats from Guerrero Negro, Baja, Mexico. FEMS Microbiol Ecol.

[42] Omoregie EO, Crumbliss LL, Bebout BM, Zehr JP (2004). Determination of nitrogen-fixing phylotypes in Lyngbya sp. and *Microcoleus chthonoplastes* cyanobacterial mats from Guerrero Negro, Baja California, Mexico. Appl Environ Microbiol.

[43] Paerl HW, Fitzpatrick M, Bebout BM (1996). Seasonal nitrogen fixation dynamics in a marine microbial mat: potential roles of cyanobacteria and microheterotrophs. Limnol Oceanogr.

[44] Severin I, Stal LJ (2010). NifH expression by five groups of phototrophs compared with nitrogenase activity in coastal microbial mats. FEMS Microbiol Ecol.

[45] Zehr JP, Wyman M, Miller V, Duguay L, Capone DG (1993). Modification of the Fe protein of nitrogenase in natural populations of *Trichodesmium thiebautii*. Appl Environ Microbiol.

[46] Steunou AS, Jensen SI, Brecht E, Becraft ED, Bateson MM, Kilian O (2008). Regulation of nif gene expression and the energetics of N_2_ fixation over the diel cycle in a hot spring microbial mat. ISME J.

[47] Guerrero MA, Jones RD (1996). Photoinhibition of marine nitrifying bacteria. I. Wavelength-dependent response. Mar Ecol Prog Ser.

[48] Black EM, Just CL (2018). The genomic potentials of NOB and comammox nitrospira in river sediment are impacted by native freshwater mussels. Front Microbiol.

[49] Daims H, Lücker S, Wagner M (2016). A new perspective on microbes formerly known as nitrite-oxidizing bacteria. Trends Microbiol.

[50] Aleem MI, Hoch GE, Varner JE (1965). Water as the source of oxidant and reductant in bacterial chemosynthesis. PNAS.

[51] Beman J, Leilei Shih J, Popp B (2013). Nitrite oxidation in the upper water column and oxygen minimum zone of the eastern tropical North Pacific Ocean. ISME J.

[52] Soler-Jofra A, Pérez J, van Loosdrecht MCM (2021). Hydroxylamine and the nitrogen cycle: a review. Water Res.

[53] Kato S, Sakai S, Hirai M, Tasumi E, Nishizawa M, Suzuki K (2018). Long-term cultivation and metagenomics reveal ecophysiology of previously uncultivated thermophiles involved in biogeochemical nitrogen cycle. Microbes Environ.

[54] Jeffries TC, Seymour JR, Newton K, Smith RJ, Seuront L, Mitchell JG (2012). Increases in the abundance of microbial genes encoding halotolerance and photosynthesis along a sediment salinity gradient. Biogeosciences.

[55] Cabello P, Roldán MD, Castillo F, Moreno-Vivián C Nitrogen cycle. In: Encyclopedia of Microbiology, 3rd edn. Schaechter M, editor. Academic Press, Oxford; 2009. p. 299–321.

[56] Klotz MG, Stein LY (2008). Nitrifier genomics and evolution of the nitrogen cycle. FEMS Microbiol Lett.

[57] Zhang X, Xia Y, Wang C, Li J, Wu P, Ma L (2020). Enhancement of nitrite production via addition of hydroxylamine to partial denitrification (PD) biomass: Functional genes dynamics and enzymatic activities. Bioresour Technol.

[58] Lees H, Simpson J, Jensen H, Sorensen H (1954). Formation of Nitrite from Oximes and Hydroxylamine by Microorganisms. Nature.

[59] Tanaka M (1953). Occurrence of Hydroxylamine in Lake Waters as an Intermediate in Bacterial Reduction of Nitrate. Nature.

[60] Ferousi C, Schmitz RA, Maalcke WJ, Lindhoud S, Versantvoort W, Jetten MSM (2021). Characterization of a nitrite-reducing octaheme hydroxylamine oxidoreductase that lacks the tyrosine cross-link. JBC.

[61] Dietl A, Maalcke WJ, Ferousi C, Jetten MSM, Kartal B (2019). Barends TRM. A 60-heme reductase complex from an anammox bacterium shows an extended electron transfer pathway. Acta Crystallogr. D. Struct. Biol..

[62] Waite DW, Vanwonterghem I, Rinke C, Parks DH, Zhang Y, Takai K (2018). Addendum: comparative genomic analysis of the class epsilonproteobacteria and proposed reclassification to epsilonbacteraeota (phyl. nov.). Front Microbiol.

[63] Haase D, Hermann B, Einsle O, Simon J (2017). Epsilonproteobacterial hydroxylamine oxidoreductase (εHao): characterization of a ‘missing link’ in the multihaem cytochrome c family. Mol Microbiol.

[64] van den Berg WAM, Hagen WR, van Dongen WMAM (2000). The hybrid-cluster protein (‘prismane protein’) from *Escherichia coli*. Eur J Biochem.

[65] Maalcke WJ, Reimann J, de Vries S, Butt JN, Dietl A, Kip N (2016). Characterization of anammox hydrazine dehydrogenase, a key N_2_-producing enzyme in the global nitrogen cycle. J Biol Chem.

[66] Campbell BJ, Smith JL, Hanson TE, Klotz MG, Stein LY, Lee CK (2009). Adaptations to submarine hydrothermal environments exemplified by the genome of *Nautilia profundicola*. PLOS Genet.

[67] Hanson T, Campbell BJ, Kalis KM, Campbell MA, Klotz MG. Nitrate ammonification by *Nautilia profundicola* AmH: experimental evidence consistent with a free hydroxylamine intermediate. Front Microbiol. 2013; 4. 10.3389/fmicb.2013.00180 PMC3701875 23847604

