# Correction to: Millimeter-scale vertical partitioning of nitrogen cycling in hypersaline mats reveals prominence of genes encoding multi-heme and prismane proteins

**DOI:** 10.1038/s41396-021-01180-w

**Published:** 2022-01-19

**Authors:** P. Maza-Márquez, M. D. Lee, A. M. Detweiler, B. M. Bebout

**Affiliations:** 1 Exobiology Branch, NASA Ames Research Center, Moffett Field, CA USA; 2 Bay Area Environmental Research Institute, Moffett Field, CA USA; 3 Blue Marble Space Institute of Science, Seattle, WA USA; 4 Chan Zuckerberg Biohub, San Francisco, CA USA

**Keywords:** Microbial ecology, Population dynamics, Population genetics, Metagenomics

Correction to: *The ISME Journal* 10.1038/s41396-021-01161-z, published online 03 December 2021

Following the publication of this article it was noted that there were a number of typographical errors. These have now been corrected.

The conclusions of the article remain unchanged.

The original article has been corrected.

