# Supplementary material for: Millimeter-scale vertical partitioning of nitrogen cycling in hypersaline mats reveals prominence of genes encoding multi-heme and prismane proteins
Source: ISME J. 2021 Dec 3;16(4):1119–29. doi: 10.1038/s41396-021-01161-z (PMC8940962; doi:10.1038/s41396-021-01161-z)
Supplement: Supplementary file 1 — Supplementary material for: Millimeter-scale vertical partitioning of nitrogen cycling in hypersaline mats reveals prominence of genes encoding multi-heme and prismane proteins [file 41396_2021_1161_MOESM1_ESM.pdf]

Supplementary material for: Millimeter-scale vertical partitioning of nitrogen cycling in hypersaline mats reveals prominence of genes encoding multi-heme and prismane proteins

Authors: P. Maza-Márquez[a], M.D. Lee[a,b], A.M. Detweiler[a, c, d], B.M. Bebout[a]

Affiliations:

[a]Exobiology Branch, NASA Ames Research Center, Moffett Field, CA, USA.

[b]Blue Marble Space Institute of Science, Seattle, WA, USA.

[c] Bay Area Environmental Research Institute, Moffett Field, California, USA.

[d]Chan Zuckerberg Biohub, San Francisco, CA, USA.

***Corresponding author:**

Paula Maza-Márquez. Exobiology Branch, NASA Ames Research Center, CA, USA

E-mail: paulamazamar@gmail.com

This PDF file includes:

Supplementary Tables S1-S2

Supplementary Figures S1-S6

Text 1. Supplementary methods Metagenomic data processing

I. Supplementary tables S1-S2

Table S1. Primers (**A**) and qPCR cycling conditions (**B**) used for the quantification of the abundance of Bacteria, Archaea, *nifH*, archaeal-*amoA*, bacterial-*amoA*, *Nitrospira-nxrB*, *nosZ*, *nirS*, and Planctomycetes-16S rRNA gene in the mat samples.

A

Group and molecular marker	Primers		References
	Name	Sequence (5'- 3')	
Bacteria	341F	CCTACGGGAGGCAGCAG	[1]
	534R	ATTACCGCGGCTGCTGG	
Archaea	UNI-b-rev	GAGGGCGGTGTGTRCAA	[2]
	ARCH915	AGGAATTGGCGGGGGA GCAC	
nifH	nifH1	GGAATTCCTGYGAYCCN AARGCNGA	[3]
	nifH2	CGGATCCGDNGCCATCA TYTCNCC	
Archaeal-*amoA*	CrenamoA23F	ATGGTCTGGCTWAGACG	[4]
	CrenamoA616R	GCCATCCATCTGTATGT CCA	
Bacterial-*amoA*	AmoA1F	GGGGTTTCTACTGGTGG T	[5]
	AmoA2R	CCCCTCKGSAAAGCCTT CTTC	

Nitrospira-nxrB	nxrBF916	GAGCAGGTGGCGCTCCCGC	[6]
	nxrBR1237	GTAGATCGGCTCTTCGACCTG	
nosZ	nosZ1840F	CGCRACGGCAASAAGGTSMSSGT	[7]
	nosZ2090R	CAKRTGCAKSGCRTGGCAGAA	
nirS	nirS4QF	AACGYSAAGGARACSGG	[8]
	nirS6QR	GASTTCGGRTGSGTCTTSAYGAA	
Planctomycetes-16S rRNA gene	A438f	GTCRGGAGTTADGAAATG	[9, 10]
	A684r	ACCAGAAGTTCCACTCTC	

B

		Amplification (×40 cycles)		
	Initial denaturalization	Denaturalization	Primers annealing	Elongation
Bacteria	95°C, 7 minutes	95°C, 30 seconds	60°C, 40 seconds	72°C, 30 seconds
Archaea	95°C, 7 minutes	95°C, 15 seconds	55˚C, 30 seconds	72°C, 45 seconds
nifH	95°C, 7 minutes	95°C, 15 seconds	54°C, 30 seconds	72°C, 45 seconds
Archaeal-*amoA*	95°C, 7 minutes	95°C, 15 seconds	52°C, 30 seconds	72°C, 45 seconds
Bacterial-*amoA*	95°C, 7 minutes	95°C, 15 seconds	60°C, 30 seconds	72°C, 45 seconds
Nitrospira-nxrB	95°C, 7 minutes	95°C, 30 seconds	68°C, 40 seconds	72°C, 30 seconds

nosZ	95°C, 7 minutes	95°C, 15 seconds	50°C, 30 seconds	72°C, 45 seconds
nirS	95°C, 7 minutes	95°C, 15 seconds	50°C, 30 seconds	72°C, 45 seconds
Plantomycetes-16S rRNA gene	95°C, 7 minutes	95°C, 30 seconds	50°C, 40 seconds	72°C, 30 seconds

Table S2. A. Number of read-pairs sequenced in each layer **B.** Assembly summary statistics (utilizing contigs > 1,000 bp)

A.

Layers	Read pairs (in millions)
Layer 1 (0-1mm)	16.8
Layer 2 (1-2mm)	25.6
Layer 3 (2-3mm)	20.4
Layer 4 (3-4mm)	35.7

B.

Summary metric	Value
Num. contigs	341,114
Num. contigs ≥ 10000 bps	7,595
Num. contigs ≥ 50000 bps	245
Largest contig	331,702 bps
Total assembly length	826 Mbps
N50	2,730
L50	67,558
Total num. of gene calls	922,324
Num. of N-related gene calls*	1,305

*Based on 65 N-related KEGG Orthology terms

II. Supplementary Figures S1-S6

Fig. S1. Number of copies of (A) Bacteria, (B) Archaea, (C) *nifH* genes, (D) Archaeal-*amoA*, (E) Bacterial-*amoA*, (F) *Nitrospira-nxrB*, (G) *nosZ*, (H) *nirS*, (I) Planctomycetes-16S rRNA gene, per ng of DNA or cDNA, quantified by qPCR and RT-qPCR in hypersaline microbial mat profiles from different depths.

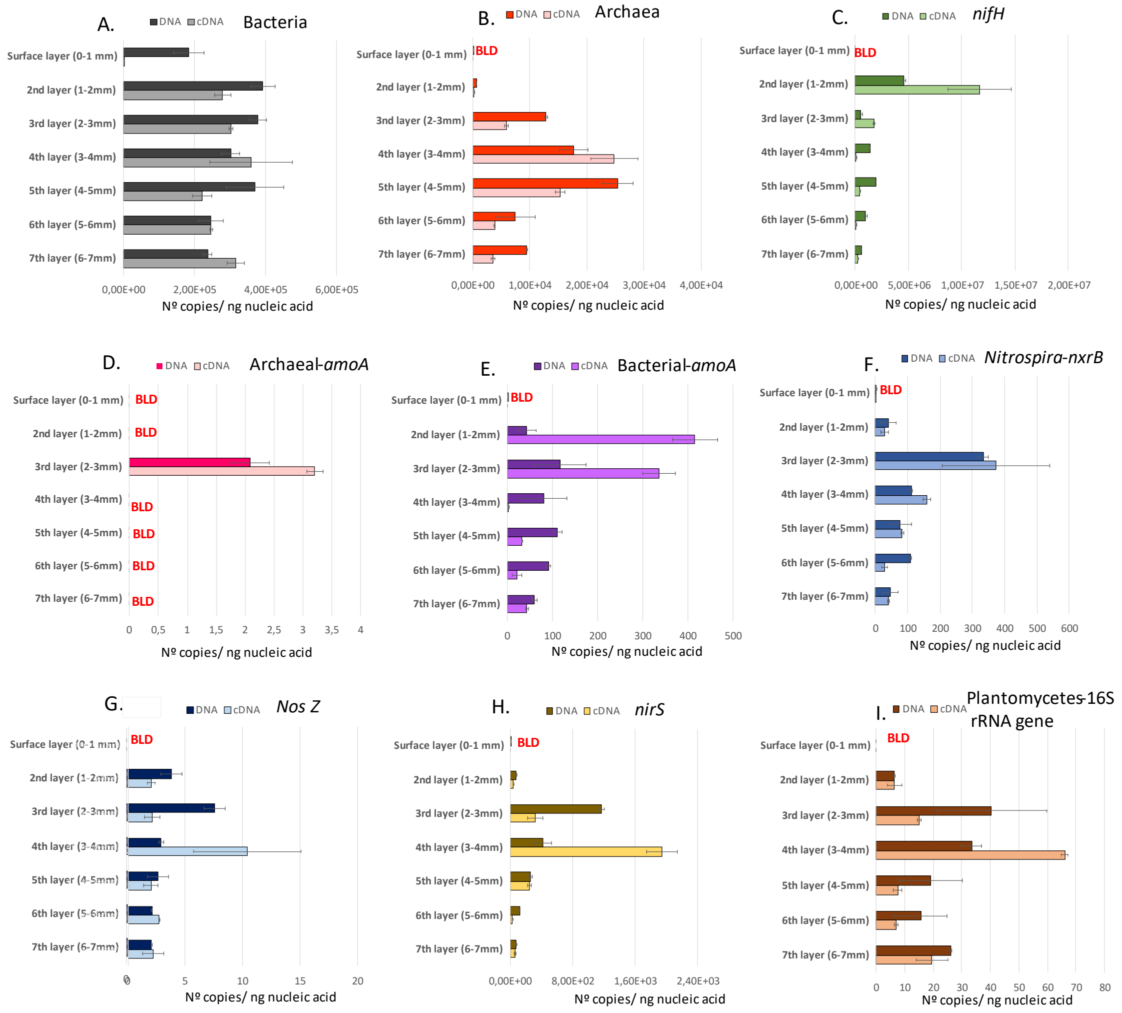

Fig. S2. Ratios of (A) Bacteria, (B) Archaea, (C) *nifH* genes, (D) Archaeal-*amoA*, (E) Bacterial-*amoA*, (F) *Nitrospira-nxrB*, (G) *nosZ*, (H) *nirS*, (I) Planctomycetes-16S rRNA gene in hypersaline microbial mat profiles from different depths.

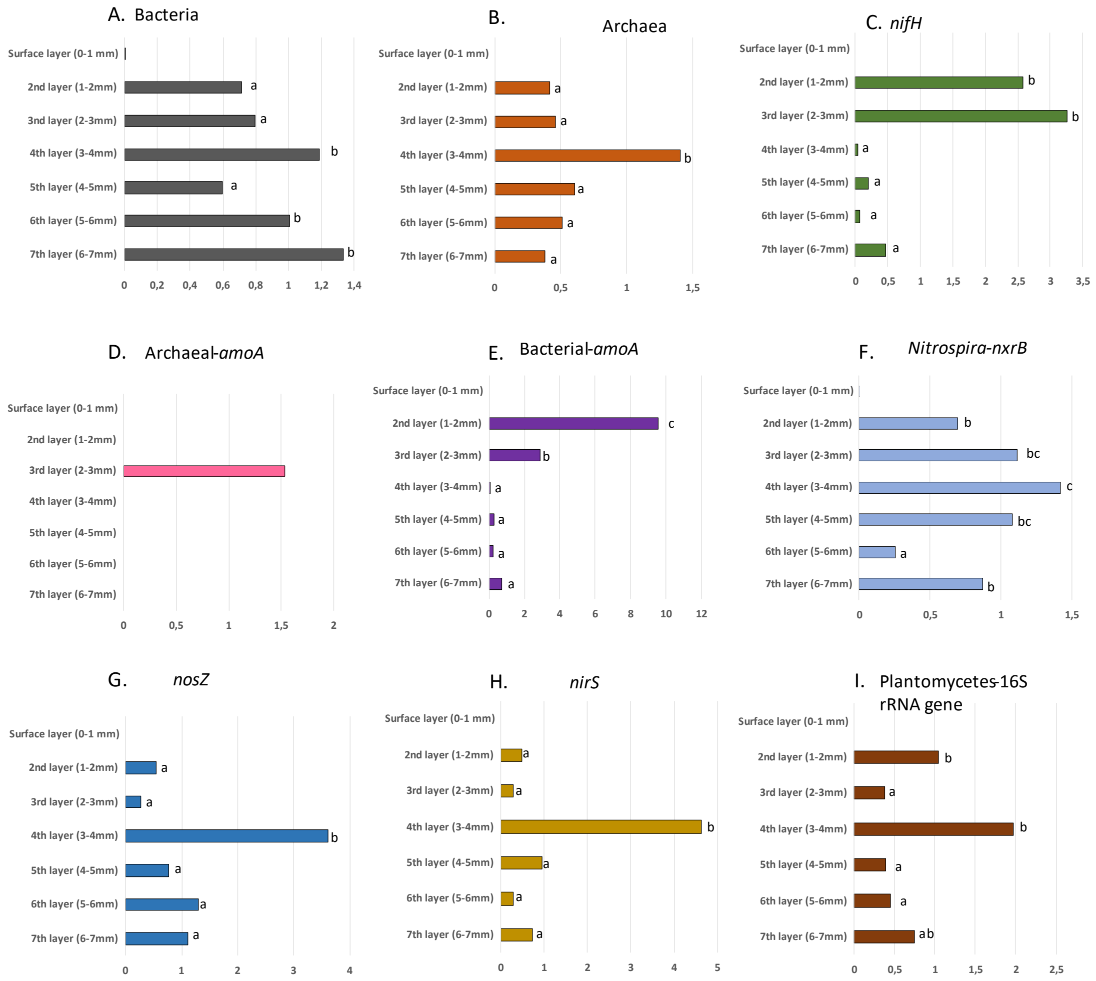

Fig. S3. Heatmap representing the relative abundance of Bacterial taxa at Phylum (A) and Genus (B) detected by read-based classification of metagenomic sequencing in the different layers [Layer 1 (0-1 mm from surface), Layer2 (1-2 mm from surface), Layer 3 (2-3 mm from surface), Layer 4 (3-4 mm from surface)].

A.

	Layer 1 (0-1 mm)	Layer 2 (1-2 mm)	Layer 3 (2-3 mm)	Layer 4 (3-4 mm)
Proteobacteria	45,50	49,95	48,30	48,48
Actinobacteria	15,10	20,53	21,40	20,89
Firmicutes	3,42	4,92	6,15	6,40
Cyanobacteria	21,87	8,93	5,66	5,89
Bacteroidetes	6,28	4,86	5,78	5,73
Planctomycetes	2,20	2,77	3,73	3,73
Euryarchaeota	0,89	1,33	1,69	1,56
Chloroflex	1,08	1,64	1,39	1,32
DeinococcusThermus	0,72	0,91	0,90	0,88
Verrucomicrobia	0,56	0,64	0,72	0,79
Ascomycota	0,55	0,66	0,73	0,75
Spirochaetes	0,33	0,56	0,62	0,67
Acidobacteria	0,31	0,37	0,41	0,41
Chlorob	0,19	0,27	0,32	0,32
Tenericutes	0,10	0,17	0,25	0,26
Kiritimatiellaeota	0,05	0,14	0,23	0,20
Nitrospirae	0,13	0,18	0,19	0,19
Fusobacteria	0,05	0,10	0,16	0,17
Gemmatimonadete	0,11	0,14	0,16	0,15
Synergistetes	0,06	0,12	0,15	0,15
Crenarchaeota	0,05	0,09	0,11	0,11
Thermotogae	0,03	0,06	0,09	0,09
Armatimonadete	0,05	0,07	0,08	0,08
Basidiomycot	0,06	0,07	0,07	0,07
NA	0,03	0,05	0,07	0,07
Lentisphaerae	0,02	0,04	0,06	0,06
Deferribacteres	0,02	0,04	0,06	0,06
Ignavibacteria	0,01	0,03	0,05	0,05
Aquificae	0,03	0,04	0,05	0,05
Calditrichaeota	0,02	0,04	0,06	0,05
Thaumarchaeota	0,02	0,04	0,05	0,05
Chlamydia	0,03	0,04	0,05	0,05
candidate division Zixibacte	0,01	0,02	0,04	0,04
Balneolaeota	0,04	0,03	0,03	0,04
Thermodesulfobacteria	0,01	0,02	0,03	0,03
Chrysiogenetes	0,02	0,03	0,03	0,03
Candidatus Bipolaricaul	0,01	0,02	0,03	0,03
Candidatus Cloacimone	0,00	0,01	0,02	0,02
Elusimicrobia	0,01	0,01	0,01	0,01
Dictyoglom	0,01	0,01	0,01	0,01
Candidatus Saccharibacte	0,01	0,01	0,01	0,01
Fibrobacteres	0,01	0,01	0,01	0,01
Candidatus Gracilibacter	0,00	0,00	0,00	0,01
Microsporidia	0,00	0,00	0,00	0,01
Caldiserica	0,00	0,00	0,00	0,00
Candidatus Micrarchaeo	0,01	0,00	0,00	0,00
Coprothermobacterota	0,00	0,00	0,00	0,00
Candidatus Korarchaeo	0,00	0,00	0,00	0,00

B.

Phylum	Family	Genus	Layer 1 (0-1 mm)	Layer 2 (1-2 mm)	Layer3 (2-3 mm)	Layer4 (3-4 mm)
Actinobacteria	*Iamiaceae*	NA	4,12	4,39	4,88	4,89
Actinobacteria	*Streptomycetaceae*	*Streptomyces*	3,74	5,24	5,43	5,25
Proteobacteria	*Pseudomonadaceae*	*Pseudomonas*	2,94	3,46	3,15	3,13
Cyanobacteria	*Synechococcaceae*	*Synechococcus*	2,42	0,99	0,66	0,70
Cyanobacteria	*Nostocaceae*	*Nostoc*	1,89	0,81	0,46	0,50
Cyanobacteria	*Oscillatoriaceae*	*Oscillatoria*	1,63	0,59	0,34	0,38
Cyanobacteria	*Calotrichaceae*	*Calothrix*	1,37	0,59	0,34	0,38
Cyanobacteria	*Leptolyngbyaceae*	*Leptolyngbya*	1,09	0,41	0,25	0,27
Proteobacteria	*Rhodobacteraceae*	*Paracoccus*	1,07	0,67	0,59	0,61
Cyanobacteria	*Microcoleaceae*	*Microcoleus*	0,93	0,43	0,17	0,21
Cyanobacteria	*Aphanothecaceae*	*Halothece*	0,91	0,37	0,56	0,38
Proteobacteria	*Rhodobacteraceae*	*Rhodobacter*	0,84	0,54	0,47	0,48
Proteobacteria	*Burkholderiaceae*	*Burkholderia*	0,80	0,95	0,94	0,93
Cyanobacteria	*Coleofasciculaceae*	*Geitlerinema*	0,77	0,26	0,17	0,18
Cyanobacteria	*Oscillatoriaceae*	*Moorea*	0,74	0,34	0,18	0,20
Proteobacteria	*Bradyrhizobiaceae*	*Bradyrhizobium*	0,70	0,74	0,75	0,77
Proteobacteria	*Sphingomonadaceae*	*Sphingomonas*	0,67	0,66	0,66	0,69
Cyanobacteria	*Prochlorotrichaceae*	*Halomicronema*	0,66	0,24	0,15	0,16
Proteobacteria	*Methylobacteriaceae*	*Methylobacterium*	0,64	0,67	0,65	0,63
Cyanobacteria	*Hyellaceae*	*Pleurocapsa*	0,64	0,22	0,12	0,14
Cyanobacteria	*Microcoleaceae*	*Arthrospira*	0,63	0,22	0,13	0,14
Actinobacteria	*Micromonosporaceae*	*Micromonospora*	0,60	0,85	0,88	0,85
Bacteroidetes	*Hymenobacteraceae*	*Hymenobacter*	0,58	0,36	0,35	0,34
Proteobacteria	*Rhodospirillaceae*	*Azospirillum*	0,58	0,65	0,62	0,61
Proteobacteria	*Halomonadaceae*	*Halomonas*	0,57	0,65	0,54	0,51
Firmicutes	*Paenibacillaceae*	*Paenibacillus*	0,56	0,70	0,80	0,81
Cyanobacteria	*Synechococcaceae*	*Dactylococcopsis*	0,56	0,14	0,14	0,11
Proteobacteria	*Erythrobacteraceae*	*Erythrobacter*	0,55	0,20	0,18	0,18
Cyanobacteria	*Aphanothecaceae*	*Gloeothece*	0,54	0,21	0,11	0,13
Actinobacteria	*Corynebacteriaceae*	*Corynebacterium*	0,54	0,69	0,73	0,71
Proteobacteria	*Phyllobacteriaceae*	*Mesorhizobium*	0,54	0,54	0,55	0,55
Proteobacteria	*Burkholderiaceae*	*Cupriavidus*	0,50	0,60	0,59	0,57
Actinobacteria	*Actinomycetaceae*	*Actinomyces*	0,50	0,68	0,71	0,69
Proteobacteria	*Rhodobacteraceae*	*Roseovarius*	0,49	0,29	0,26	0,26
Proteobacteria	*Polyangiaceae*	*Sorangium*	0,45	0,57	0,54	0,52
Actinobacteria	*Microbacteriaceae*	*Microbacterium*	0,44	0,59	0,62	0,60
Actinobacteria	*Mycobacteriaceae*	*Mycobacterium*	0,43	0,57	0,58	0,58
DeinococcusThermus	*Deinococcaceae*	*Deinococcus*	0,43	0,56	0,55	0,53
Proteobacteria	*Rhizobiaceae*	*Rhizobium*	0,42	0,46	0,48	0,48
Proteobacteria	*Burkholderiaceae*	*Paraburkholderia*	0,42	0,45	0,45	0,45
Proteobacteria	*Desulfovibrionaceae*	*Desulfovibrio*	0,41	0,80	1,05	1,06
Actinobacteria	*Nocardiaceae*	*Nocardia*	0,41	0,58	0,58	0,58
Proteobacteria	*Rhodobacteraceae*	*Rhodovulum*	0,41	0,23	0,21	0,22
Actinobacteria	*Nocardioidaceae*	*Nocardioides*	0,40	0,59	0,60	0,58
Proteobacteria	*Rhodobacteraceae*	*Sulfitobacter*	0,39	0,25	0,22	0,23
Proteobacteria	*Oxalobacteraceae*	*Massilia*	0,39	0,47	0,45	0,44
Proteobacteria	*Ectothiorhodospiraceae*	*Thioalkalivibrio*	0,39	0,55	0,37	0,31
Actinobacteria	*Mycobacteriaceae*	*Mycolicibacterium*	0,37	0,44	0,45	0,45
Actinobacteria	*Nocardiaceae*	*Rhodococcus*	0,37	0,44	0,46	0,45
Firmicutes	*Bacillaceae*	*Bacillus*	0,35	0,44	0,56	0,70
Proteobacteria	*Alcaligenaceae*	*Bordetella*	0,34	0,46	0,46	0,46
Bacteroidetes	*Flavobacteriaceae*	*Flavobacterium*	0,34	0,24	0,29	0,30
Proteobacteria	*Xanthomonadaceae*	*Stenotrophomonas*	0,34	0,40	0,36	0,36
Proteobacteria	*Alcaligenaceae*	*Achromobacter*	0,34	0,39	0,38	0,37
Actinobacteria	*Pseudonocardiaceae*	*Amycolatopsis*	0,34	0,45	0,47	0,47
Proteobacteria	*Xanthomonadaceae*	*Lysobacter*	0,34	0,38	0,35	0,34
Proteobacteria	*Desulfobacteraceae*	*Desulfosarcina*	0,34	1,21	1,49	1,62
Proteobacteria	*Geobacteraceae*	*Geobacter*	0,33	0,58	0,72	0,72
Proteobacteria	*Xanthomonadaceae*	*Xanthomonas*	0,33	0,36	0,32	0,32
Proteobacteria	*Rhodobacteraceae*	*Celeribacter*	0,32	0,19	0,16	0,17
Proteobacteria	*Caulobacteraceae*	*Caulobacter*	0,27	0,31	0,30	0,30
Proteobacteria	*Vibrionaceae*	*Vibrio*	0,26	0,27	0,30	0,30
Chloroflexi	*Roseiflexaceae*	*Roseiflexus*	0,26	0,30	0,16	0,16
Proteobacteria	*Sphingomonadaceae*	*Sphingobium*	0,25	0,27	0,29	0,29
Proteobacteria	*Comamonadaceae*	*Variovorax*	0,25	0,32	0,32	0,31
Proteobacteria	*Zoogloeaceae*	*Azoarcus*	0,25	0,28	0,26	0,26
Proteobacteria	*Bradyrhizobiaceae*	*Bosea*	0,25	0,27	0,26	0,26
Chloroflexi	*Ardenticatenaceae*	*CandidatusPromineofilum*	0,25	0,46	0,38	0,37
Proteobacteria	*Chromatiaceae*	*Thiodictyon*	0,24	0,38	0,31	0,29
Bacteroidetes	*Flavobacteriaceae*	*Chryseobacterium*	0,23	0,24	0,31	0,32
Proteobacteria	*Bradyrhizobiaceae*	*Rhodopseudomonas*	0,23	0,24	0,24	0,24
Proteobacteria	*Aeromonadaceae*	*Aeromonas*	0,23	0,28	0,26	0,26
Proteobacteria	*Myxococcaceae*	*Myxococcus*	0,23	0,31	0,31	0,30
Proteobacteria	*Caulobacteraceae*	*Brevundimonas*	0,22	0,24	0,23	0,23
Proteobacteria	*Alteromonadaceae*	*Marinobacter*	0,22	0,27	0,25	0,24
Actinobacteria	*Micrococcaceae*	*Arthrobacter*	0,21	0,30	0,31	0,31
Proteobacteria	*Rhizobiaceae*	*Sinorhizobium*	0,21	0,22	0,22	0,22
Actinobacteria	*Frankiaceae*	*Frankia*	0,21	0,28	0,28	0,27
Proteobacteria	*Sphingomonadaceae*	*Sphingopyxis*	0,21	0,20	0,20	0,20
Proteobacteria	*Sphingomonadaceae*	*Novosphingobium*	0,20	0,21	0,21	0,21
Proteobacteria	*Burkholderiaceae*	*Pandoraea*	0,19	0,21	0,20	0,20
Firmicutes	*Lactobacillaceae*	*Lactobacillus*	0,19	0,26	0,32	0,32
Proteobacteria	*Comamonadaceae*	*Acidovorax*	0,19	0,25	0,22	0,22
Actinobacteria	*Gordoniaceae*	*Gordonia*	0,18	0,24	0,25	0,24
Actinobacteria	*Pseudonocardiaceae*	*Pseudonocardia*	0,18	0,24	0,25	0,25
Proteobacteria	*Chromatiaceae*	*Thioflavicoccus*	0,18	0,30	0,24	0,23
Firmicutes	*Clostridiaceae*	*Clostridium*	0,17	0,33	0,48	0,50
Proteobacteria	*Chromatiaceae*	*Marichromatium*	0,16	0,26	0,21	0,20
Proteobacteria	*Desulfobacteraceae*	*Desulfococcus*	0,15	0,50	0,68	0,74
Proteobacteria	*Desulfovibrionaceae*	*Pseudodesulfovibrio*	0,12	0,25	0,33	0,34

Fig. S4. Phylogenetic tree for *narG, narZ, nxrA* (K00370)

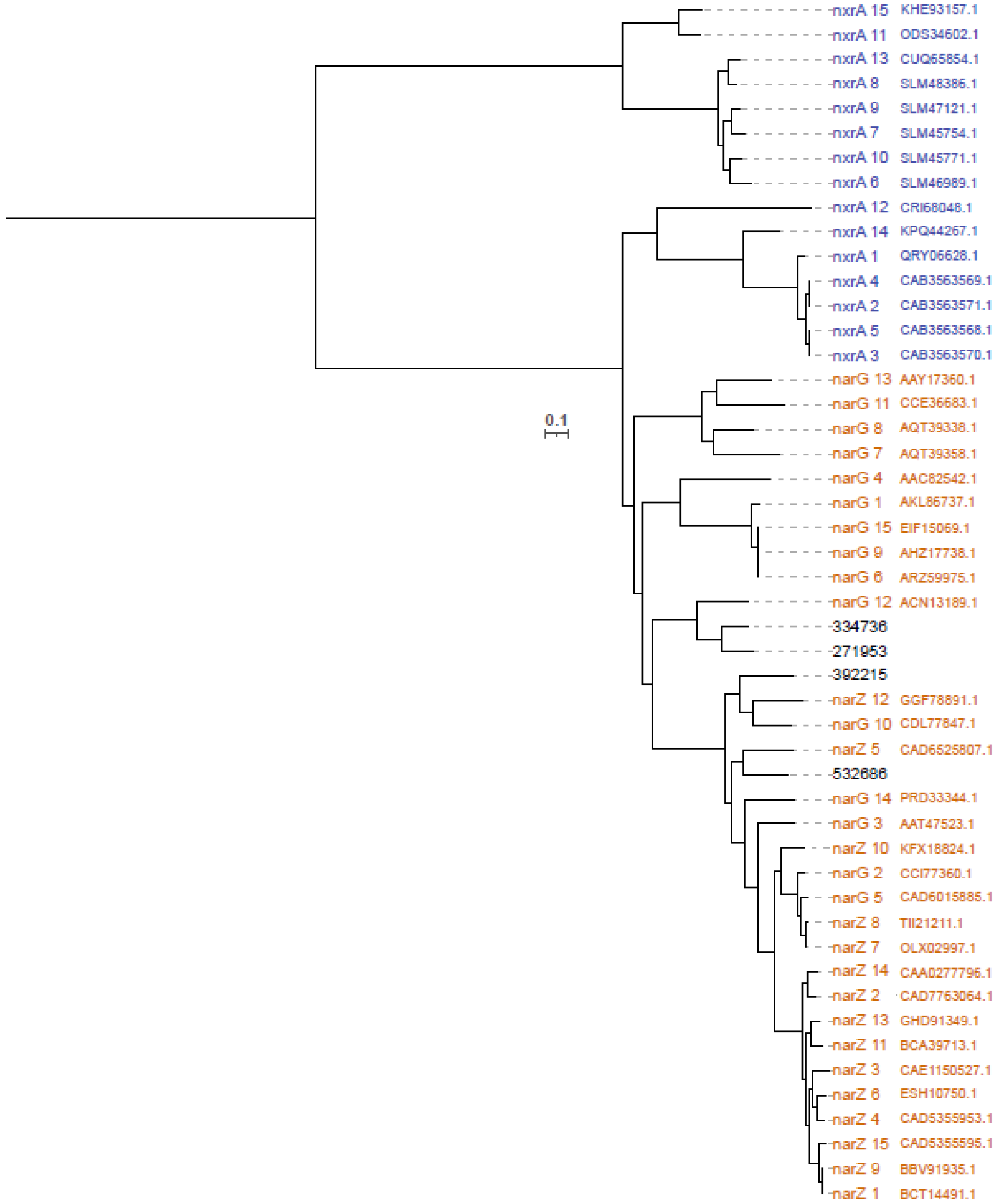

Fig. S5. Phylogenetic tree for *narH*, *narY*, *nxrB* (K00371)

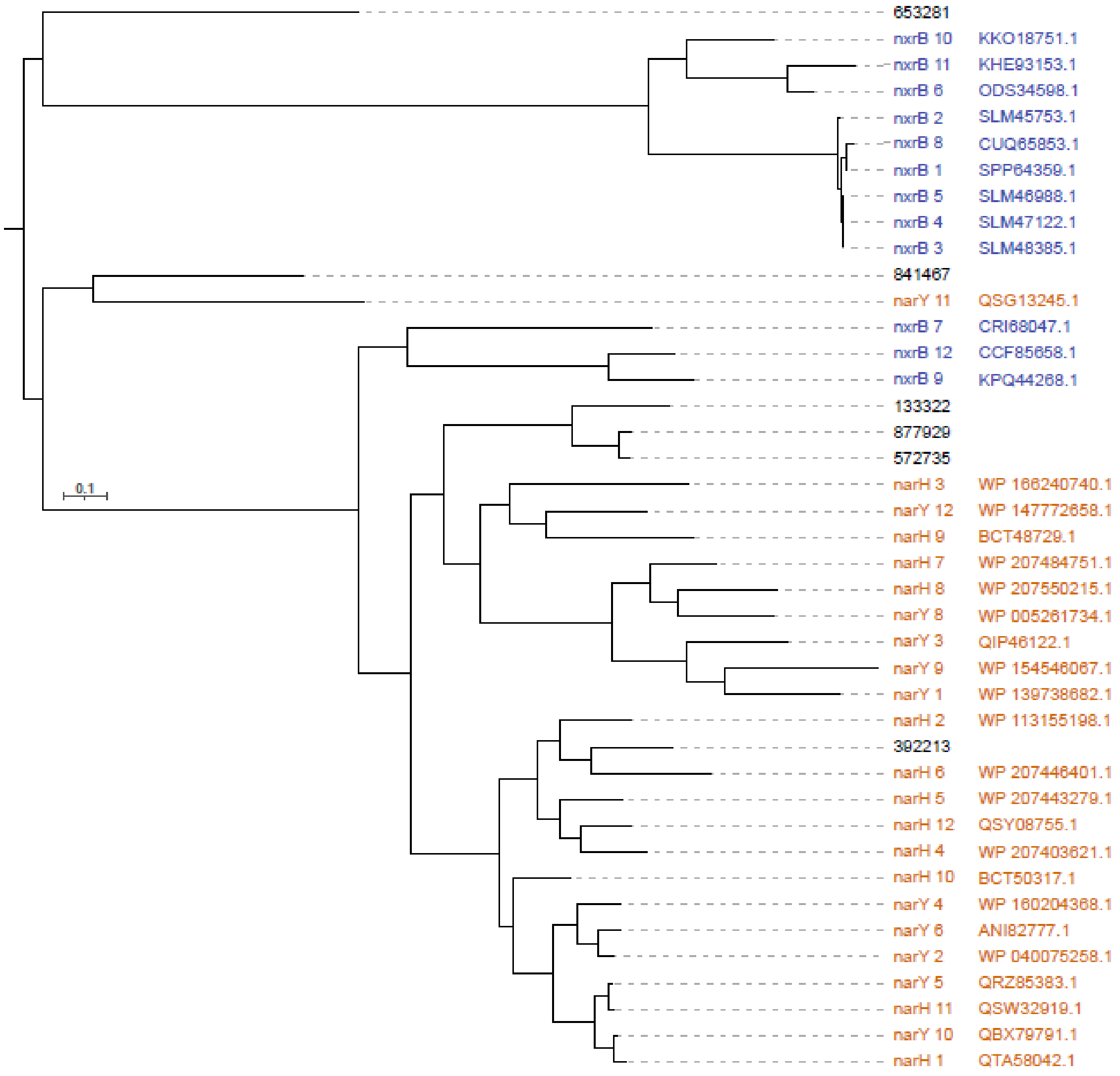

Fig. S6. Partial visualization of the multiple sequence alignment of 52 HAO proteins sequences detected in our study, plus 10 additional HAO sequences from AOB *Nitrosomonas europaea* and *Nitrosomonas mobilis*, HAO from anammox bacteria *Kuenenia stuttgartiensis* and HAO from Campylobacterota (*Campylobacter fecus*, *Campylobacter curvus*, *Caminibacter mediatlanticus* and *Nautilia profundicola)*. HAO from Deltaproteobacteria *(Maridesulfovibrio hydrothermalis)*, HAO from Thermodesulfobacteria *(Thermodesulfatator atlanticus)*, HAO from Gammaproteobacteria *(Oceanospirillum beijerinckii)*.

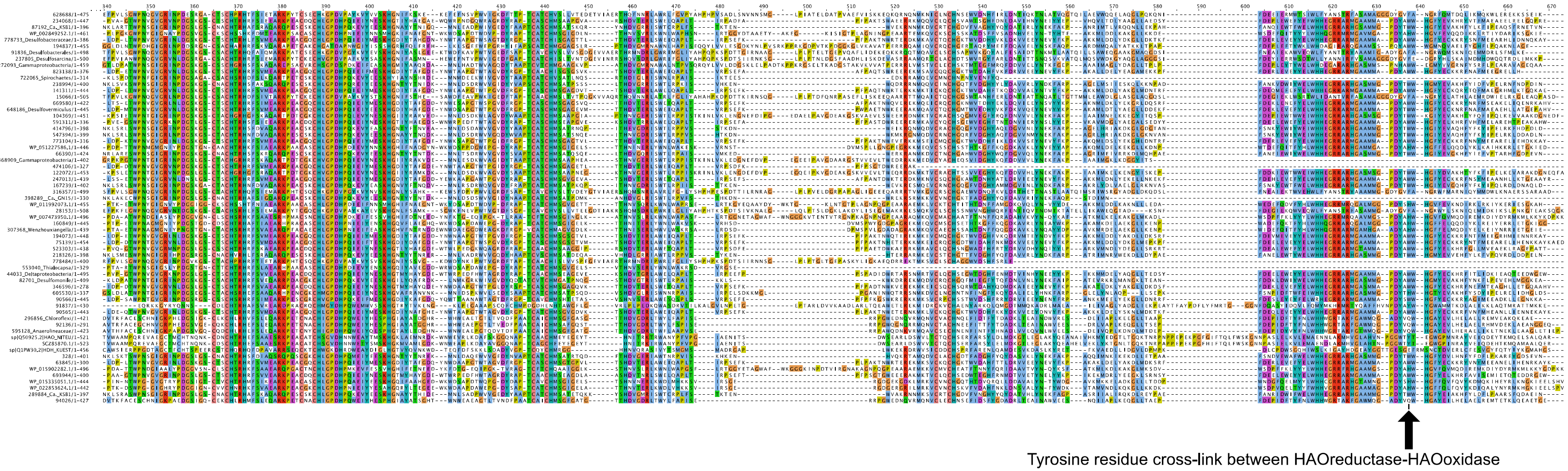


```
sp|Q1PW30.2|HDH_KUEST          ------------------------------------------------------------ 0
91837                          ------------------------------------------------------------ 0
sp|Q50925.2|HAO_NITEU          ------------------------------------------------------------ 0
SCZ85870.1                     ------------------------------------------------------------ 0
```

```
28153                        ------------------------------------------------------------ 0
316357                       ------------------------------------------------------------ 0
91836_Desulfobacterales      ------------------------------------------------------------ 0
778484                       ------------------------------------------------------------ 0
237805_Desulfosarcina        ------------------------------------------------------------ 0
15066                        ------------------------------------------------------------ 0
628688                       ------------------------------------------------------------ 0
90566                        LAASNKMLRYKLFFVFLSLIAVSLAGTVPAGATVDFSASTRLSCVYCHENPAGDDTLTPE 60
648186_Desulfovermiculus     ------------------------------------------------------------ 0
346596                       ------------------------------------------------------------ 0
247013                       ------------------------------------------------------------ 0
474106                       ------------------------------------------------------------ 0
75139                        ------------------------------------------------------------ 0
823138                       ------------------------------------------------------------ 0
669580                       ------------------------------------------------------------ 0
141311                       ------------------------------------------------------------ 0
773104                       ------------------------------------------------------------ 0
778733_Desulfobacteraceae    ------------------------------------------------------------ 0
90565                        ------------------------------------------------------------ 0
63845                        ------------------------------------------------------------ 0
194073                       ------------------------------------------------------------ 0
523303                       ------------------------------------------------------------ 0
693944                       ------------------------------------------------------------ 0
```

```
234068                        --MK---LPKYLTIILILLFIFLNLRGEPATATIEYAQRTGKGCSFCHRDNNGG-PLNAV 54
44033_Deltaproteobacteria     --MS-LRWKRLVFIVF-FFSAVFFFSAPHLTATPEFAEQTEKSCAFCHDGPNGG-PLTTA 55
591311                        ------------------------------------------------------------ 0
WP_015335051.1                ------------------------------------------------------------ 0
WP_022853624.1                ------------------------------------------------------------ 0
WP_007473950.1                ------------------------------------------------------------ 0
WP_015902282.1                ------------------------------------------------------------ 0
WP_002849252.1                ------------------------------------------------------------ 0
WP_011992073.1                ------------------------------------------------------------ 0
307368_Wenzhouxiangella       ------------------------------------------------------------ 0
553040_Thialocapsa            ------------------------------------------------------------ 0
WP_051227586.1                ------------------------------------------------------------ 0
160849                        ------------------------------------------------------------ 0
595128_Anaerolineaceae        ------------------------------------------------------------ 0
94026                         ------------------------------------------------------------ 0
296856_Chloroflexi            ------------------------------------------------------------ 0
92136                         ------------------------------------------------------------ 0
167239                        ------------------------------------------------------------ 0
414796                        ------------------------------------------------------------ 0
547394                        ------------------------------------------------------------ 0
218326                        ------------------------------------------------------------ 0
328                           ------------------------------------------------------------ 0
87192_Ca._KSB1                ------------------------------------------------------------ 0
```

```
289884_Ca._KSB1               ------------------------------------------------------------ 0
66390                         ------------------------------------------------------------ 0
238994                        ------------------------------------------------------------ 0
398289__Ca._GN15              ------------------------------------------------------------ 0
605530                        ------------------------------------------------------------ 0
722065_Spirochaetes           ------------------------------------------------------------ 0
82701_Desulfomonile           ------------------------------------------------------------ 0
468909_Gammaproteobacteria    ------------------------------------------------------------ 0
104369                        ------------------------------------------------------------ 0
122072                        ------------------------------------------------------------ 0
194837                        ------------------------------------------------------------ 0
272093_Gammaproteobacteria    ------------------------------------------------------------ 0
```

```
sp|Q1PW30.2|HDH_KUEST           ------------------------------------------------------------ 0
91837                           ------------------------------------------------------------ 0
sp|Q50925.2|HAO_NITEU           ------------------------------------------------------------ 0
SCZ85870.1                      ------------------------------------------------------------ 0
28153                           ------------------------------------------------------------ 0
316357                          ------------------------------------------------------------ 0
91836_Desulfobacterales         ------------------------------------------------------------ 0
778484                          ------------------------------------------------------------ 0
237805_Desulfosarcina           ------------------------------------------------------------ 0
15066                           ------------------------------------------------------------ 0
628688                          ------------------------------------------------------------ 0
90566                           GNAFRAAGYRLPKEEK--------PSLIKNTGRLFLGFVHVLAAFIWLGTIFYVHLFMGP 112
648186_Desulfovermiculus        ------------------------------------------------------------ 0
346596                          ------------------------------------------------------------ 0
247013                          ------------------------------------------------------------ 0
474106                          ------------------------------------------------------------ 0
75139                           ------------------------------------------------------------ 0
823138                          ------------------------------------------------------------ 0
669580                          ------------------------------------------------------------ 0
141311                          ------------------------------------------------------------ 0
773104                          ------------------------------------------------------------ 0
778733_Desulfobacteraceae       ------------------------------------------------------------ 0
90565                           ------------------------------------------------------------ 0
```

```
63845                       ------------------------------------------------------------ 0
194073                      ------------------------------------------------------------ 0
523303                      -------------------------------------------GVVFFGAIFYIHIFVRP 17
693944                      ------------------------------------------------------------ 0
234068                      GIAYVRNNYDYPIPERILDKSRQLSTPVHQTARLICGYIHLVAVCVLVGTIFYIHLFVKP 114
44033_Deltaproteobacteria   GKAFIRNGYSYPIPKRIIDKTIQLESPFHRTVRGILGYIHLITAAIFVGTIFFVHMFLKP 115
591311                      ------------------------------------------------------------ 0
WP_015335051.1              ------------------------------------------------------------ 0
WP_022853624.1              ------------------------------------------------------------ 0
WP_007473950.1              ------------------------------------------------------------ 0
WP_015902282.1              ------------------------------------------------------------ 0
WP_002849252.1              ------------------------------------------------------------ 0
WP_011992073.1              ------------------------------------------------------------ 0
307368_Wenzhouxiangella     ------------------------------------------------------------ 0
553040_Thialocapsa          ------------------------------------------------------------ 0
WP_051227586.1              ------------------------------------------------------------ 0
160849                      ------------------------------------------------------------ 0
595128_Anaerolineaceae      ------------------------------------------------------------ 0
94026                       ------------------------------------------------------------ 0
296856_Chloroflexi          ------------------------------------------------------------ 0
92136                       ------------------------------------------------------------ 0
167239                      ------------------------------------------------------------ 0
414796                      ------------------------------------------------------------ 0
```

```
547394                          ------------------------------------------------------------ 0
218326                          ------------------------------------------------------------ 0
328                             ------------------------------------------------------------ 0
87192_Ca._KSB1                  ------------------------------------------------------------ 0
289884_Ca._KSB1                 ------------------------------------------------------------ 0
66390                           ------------------------------------------------------------ 0
238994                          ------------------------------------------------------------ 0
398289__Ca._GN15                ------------------------------------------------------------ 0
605530                          ------------------------------------------------------------ 0
722065_Spirochaetes             ------------------------------------------------------------ 0
82701_Desulfomonile             ------------------------------------------------------------ 0
468909_Gammaproteobacteria      ------------------------------------------------------------ 0
104369                          ------------------------------------------------------------ 0
122072                          ------------------------------------------------------------ 0
194837                          ------------------------------------------------------------ 0
272093_Gammaproteobacteria      ------------------------------------------------------------ 0
sp|Q1PW30.2|HDH_KUEST           ------------------------------------------------------------ 0
91837                           ------------------------------------------------------------ 0
sp|Q50925.2|HAO_NITEU           ------------------------------------------------------------ 0
SCZ85870.1                      ------------------------------------------------------------ 0
28153                           ------------------------------------------------------------ 0
316357                          ------------------------------------------------------------ 0
91836_Desulfobacterales         ------------------------------------------------------------ 0
```

778484	--	0
237805_Desulfosarcina	--	0
15066	--	0
628688	--	0
90566	RSLTGGLPKSEVMLGRISILVIATTGILLSVIRFRNASEIFSTTFGIVWTVKVILFLMMV	172
648186_Desulfovermiculus	--	0
346596	--	0
247013	--	0
474106	--	0
75139	--	0
823138	--	0
669580	--	0
141311	--	0
773104	--	0
778733_Desulfobacteraceae	--	0
90565	--	0
63845	--	0
194073	--	0
523303	SRLTGGLPKHERMLGLSCMAVLAVTGGYLTWVRIGRWEQFFDNTFGLMLFAKICLFLLMG	77
693944	--	0
234068	ANITGGIPRGERILGLICLAVLLVTGAYLTWYRLDSFAGFFASHFGILLFIKLLLFALMV	174
44033_Deltaproteobacteria	RSLRGGIPGGEKKLGLSCMSILAASGIYLTWYRLDSAAAFFDSRFGVLLFIKILLFVLLL	175
591311	--	0

282	WP_015335051.1	--	0
283	WP_022853624.1	--	0
284	WP_007473950.1	--	0
285	WP_015902282.1	--	0
286	WP_002849252.1	--	0
287	WP_011992073.1	--	0
288	307368_Wenzhouxiangella	--	0
289	553040_Thialocapsa	--	0
290	WP_051227586.1	--	0
291	160849	--	0
292	595128_Anaerolineaceae	--	0
293	94026	--	0
294	296856_Chloroflexi	--	0
295	92136	--	0
296	167239	--	0
297	414796	--	0
298	547394	--	0
299	218326	--	0
300	328	--	0
301	87192_Ca._KSB1	--	0
302	289884_Ca._KSB1	--	0
303	66390	--	0
304	238994	--	0

```
398289__Ca._GN15             ------------------------------------------------------------ 0
605530                       ------------------------------------------------------------ 0
722065_Spirochaetes          ------------------------------------------------------------ 0
82701_Desulfomonile          ------------------------------------------------------------ 0
468909_Gammaproteobacteria   ------------------------------------------------------------ 0
104369                       ------------------------------------------------------------ 0
122072                       ------------------------------------------------------------ 0
194837                       ------------------------------------------------------------ 0
272093_Gammaproteobacteria   ------------------------------------------------------------ 0
```

```
sp|Q1PW30.2|HDH_KUEST         ------------------------------------------------------------ 0
91837                         ------------------------------------------------------------ 0
sp|Q50925.2|HAO_NITEU         ------------------------------------------------------------ 0
SCZ85870.1                    ------------------------------------------------------------ 0
28153                         ------------------------------------------------------------ 0
316357                        ------------------------------------------------------------ 0
91836_Desulfobacterales       ------------------------------------------------------------ 0
778484                        ------------------------------------------------------------ 0
237805_Desulfosarcina         ------------------------------------------------------------ 0
15066                         ------------------------------------------------------------ 0
628688                        ------------------------------------------------------------ 0
90566                         AIAAFVTTRVDRLLKERKPPG------------------------------------ADL 196
648186_Desulfovermiculus      ------------------------------------------------------------ 0
346596                        ------------------------------------------------------------ 0
247013                        ------------------------------------------------------------ 0
474106                        ------------------------------------------------------------ 0
75139                         ------------------------------------------------------------ 0
823138                        ------------------------------------------------------------ 0
669580                        ------------------------------------------------------------ 0
141311                        ------------------------------------------------------------ 0
773104                        ------------------------------------------------------------ 0
778733_Desulfobacteraceae     ------------------------------------------------------------ 0
90565                         ------------------------------------------------------------ 0
```

63845 -- 0
194073 -- 0
523303 ALGLTAVTTIHRRMKSEAATGNE------------------------VDEGEITAANLSR 113
693944 -- 0
234068 ILALLAVTVIHRRMRAEARAQAAAV-----------------------DKREVTSDTLAQ 211
44033_Deltaproteobacteria ALGILAVTLINKRMRREAQGQSGGSGDGAAAAQARTGSGGASGTAAGAPPDGFSVDNLGR 235
591311 -- 0
WP_015335051.1 -- 0
WP_022853624.1 -- 0
WP_007473950.1 -- 0
WP_015902282.1 -- 0
WP_002849252.1 -- 0
WP_011992073.1 -- 0
307368_Wenzhouxiangella -- 0
553040_Thialocapsa -- 0
WP_051227586.1 -- 0
160849 -- 0
595128_Anaerolineaceae -- 0
94026 -- 0
296856_Chloroflexi -- 0
92136 -- 0
167239 -- 0
414796 -- 0

```
547394                          ------------------------------------------------------------ 0
218326                          ------------------------------------------------------------ 0
328                             ------------------------------------------------------------ 0
87192_Ca._KSB1                  ------------------------------------------------------------ 0
289884_Ca._KSB1                 ------------------------------------------------------------ 0
66390                           ------------------------------------------------------------ 0
238994                          ------------------------------------------------------------ 0
398289__Ca._GN15                ------------------------------------------------------------ 0
605530                          ------------------------------------------------------------ 0
722065_Spirochaetes             ------------------------------------------------------------ 0
82701_Desulfomonile             ------------------------------------------------------------ 0
468909_Gammaproteobacteria      ------------------------------------------------------------ 0
104369                          ------------------------------------------------------------ 0
122072                          ------------------------------------------------------------ 0
194837                          ------------------------------------------------------------ 0
272093_Gammaproteobacteria      ------------------------------------------------------------ 0
```

sp|Q1PW30.2|HDH_KUEST	--	0
91837	--	0
sp|Q50925.2|HAO_NITEU	--	0
SCZ85870.1	--	0
28153	--	0
316357	--	0
91836_Desulfobacterales	--	0
778484	--	0
237805_Desulfosarcina	--	0
15066	--	0
628688	--	0
90566	PDGSEGQATRFIYDGHIYDASGSGMWKNGTHMGRHHAGGDLTKAMDGAPHGAEVLEKIKY	256
648186_Desulfovermiculus	--	0
346596	--	0
247013	--	0
474106	--	0
75139	--	0
823138	--	0
669580	--	0
141311	--	0
773104	--	0
778733_Desulfobacteraceae	--	0
90565	--	0

```
63845                          ------------------------------------------------------------ 0
194073                         ------------------------------------------------------------ 0
523303                         FDGSGGKPAYVAFEGVLYDVTGSDKWKDGRHFGKHAAGSDLTGALGGAPHGAEVLEKFSR 173
693944                         ------------------------------------------------------------ 0
234068                         YDGTNGKPAYILYQDTVYDVTDSPKWKEGKHFRKHAAGRDLTTDLAGAPHADEVFADLPV 271
44033_Deltaproteobacteria      YDGKEGRPAYVMYRNKVYDVTGSQKWKEGSHFTQHAAGTDLTKAMGNAPHTEEVLSRFPV 295
591311                         ------------------------------------------------------------ 0
WP_015335051.1                 ------------------------------------------------------------ 0
WP_022853624.1                 ------------------------------------------------------------ 0
WP_007473950.1                 ------------------------------------------------------------ 0
WP_015902282.1                 ------------------------------------------------------------ 0
WP_002849252.1                 ------------------------------------------------------------ 0
WP_011992073.1                 ------------------------------------------------------------ 0
307368_Wenzhouxiangella        ------------------------------------------------------------ 0
553040_Thialocapsa             ------------------------------------------------------------ 0
WP_051227586.1                 ------------------------------------------------------------ 0
160849                         ------------------------------------------------------------ 0
595128_Anaerolineaceae         ------------------------------------------------------------ 0
94026                          ------------------------------------------------------------ 0
296856_Chloroflexi             ------------------------------------------------------------ 0
92136                          ------------------------------------------------------------ 0
167239                         ------------------------------------------------------------ 0
414796                         ------------------------------------------------------------ 0
```

```
547394                          ------------------------------------------------------------ 0
218326                          ------------------------------------------------------------ 0
328                             ------------------------------------------------------------ 0
87192_Ca._KSB1                  ------------------------------------------------------------ 0
289884_Ca._KSB1                 ------------------------------------------------------------ 0
66390                           ------------------------------------------------------------ 0
238994                          ------------------------------------------------------------ 0
398289__Ca._GN15                ------------------------------------------------------------ 0
605530                          ------------------------------------------------------------ 0
722065_Spirochaetes             ------------------------------------------------------------ 0
82701_Desulfomonile             ------------------------------------------------------------ 0
468909_Gammaproteobacteria      ------------------------------------------------------------ 0
104369                          ------------------------------------------------------------ 0
122072                          ------------------------------------------------------------ 0
194837                          ------------------------------------------------------------ 0
272093_Gammaproteobacteria      ------------------------------------------------------------ 0
```

```
sp|Q1PW30.2|HDH_KUEST          --------------------MRKF--------LKVTLASALIGCGVIGTVSSLMVKEAKA 32
91837                          -------------------------------VKNRVML--VLGMVVLVWSFGSF------ 21
sp|Q50925.2|HAO_NITEU          -------------------------MRIGEWMRGLLLCAGLMMCGVVHADIS-------T 28
SCZ85870.1                     -------------------------MHAGFWLKGVLLACGLMVAGGIQANIS-------S 28
28153                          --------------------MY-----SGYEMISR---LL-VILGVFLCLLVSQ---A-- 26
316357                         -------------------------------MRNI---LAAAILALLWA----P---T-- 17
91836_Desulfobacterales        -------------------------------MKSI---VWMFVSGILLTSLGVG---G-- 21
778484                         ------------------------------HMRRV---VFLFCAVLFFPCSGF------- 20
237805_Desulfosarcina          -------------------------------MR----HANLFVSVVLIVLLTAS---S-- 20
15066                          -------------------------------MHRI-ASLGWLFA-AFLILPGSV---Q-- 22
628688                         ------------------------------------------------------------ 0
90566                          VGTAEKES---A--DKTPPVMKLFEILARSAL----VFGVLILLCVSWWEWGPP---LV- 303
648186_Desulfovermiculus       ---------------------MRPWSTV----------PVTVLFMTALWLCIP------- 22
346596                         ------------------------------------------------------------ 0
247013                         --------------------MKRY------------I---ILGIAAA-----------VA 14
474106                         -------------------------------MENR-MFFKTAAVAAALVLIV-------- 20
75139                          --------------------MKRFW-----------TMGSI-GLAAICLMVAAN---ALG 25
823138                         ------------------------------------------------------------ 0
669580                         ------------------------------------------------------------ 0
141311                         -------------------------------MRKG-AVVVF-CLAAALWLA-------SG 20
773104                         ------------------------------------------------------------ 0
778733_Desulfobacteraceae      ------------------------------------------------------------ 0
90565                          -------------------------------MKNR-LFIII-GVVSAFWMV-------LA 20
```

63845	--	0
194073	--------------------MKKLHIQVAALIVSG-VFLVA-GV----FLV--------A	26
523303	VGEVAAGDDEP----PRTPSAGRIF--VFMAYTNL-VIVFLILACIAVWRLGFP---ASI	223
693944	--	0
234068	ITTIA--EPGEA--KKSLSLTRKIY--IGFAYANS-IITFLILLCVSGWLWGFP---FEV	321
44033_Deltaproteobacteria	VAAASEEEKGSG--RKAFGIAQRIF--VTMAYVNL-AIVILILLCVGAWLFGFP---RFS	347
591311	--	0
WP_015335051.1	---------------------------MLKQMIKV-MTVTMLIAL---SA--AL------	21
WP_022853624.1	-----------------------------MRVRSF-LSLSFLLALIIGVL--GS------	22
WP_007473950.1	-------------------------------MKKV-LSAGLSLAAISSLAFAAN------	22
WP_015902282.1	-------------------------------MKKA-LTAGLSVAAIASFAFAAN------	22
WP_002849252.1	-------------------------------MLKK-FI--LALTCIAAIGFADS------	20
WP_011992073.1	-------------------------------MFKK-VA--ILLACLVSFGFATG------	20
307368_Wenzhouxiangella	--------------------------------MKM-LR--MMAACAMTAFLA--------	17
553040_Thialocapsa	--------------------MNKDT--VLARGLVL-LT--LLVAAMLAAGMEPA---YGQ	32
WP_051227586.1	--------------------MANH----YQ--YII-LV--SLILCIILLG----------	21
160849	--------------------MSKI-----T--ERL-VA--LVGICLLGLS----------	20
595128_Anaerolineaceae	---------------------SSAR--LLILGLVAILIVAGLTITVMAVGVGGE---TA-	33
94026	-MAVNDSYSEPKSAGRSQNIIRSTR--VLIIGLVVVILVLGLGLIMLAIGRSGT---TS-	53
296856_Chloroflexi	-----------MTENNQTNSVYSAR--ILVIGLVAVIVVLGLALLVMVLGEAGA---TP-	43
92136	--	0
167239	-------------------------------MTRVLVILAAF---VLAASF---------	17
414796	-------------------------------MKRVILTIGLL---VLL------------	14

```
547394                           ------------------------------AMKYSFLLLALA---VLP------------ 15
218326                           --------------------------------MKRIPGLVMV--MVLISG---------- 16
328                              -------------------------------MIR--------ACLIALCGLAFL---ASG 18
87192_Ca._KSB1                   -----------------------------------MHMYRLVLMILVSA----------- 14
289884_Ca._KSB1                  -----------------------------------MPLRQLLLYLLLST----------- 14
66390                            -------------------------------MLRSHPFLVLPLLLALVSGVSGT---PSG 26
238994                           -------------------------------MNRSIVMSMIVFVLLCYC----------- 18
398289__Ca._GN15                 ---------------------------MKMREVRRLAVAVIIVVIAMSS----------- 22
605530                           ------------------------------------------------------------ 0
722065_Spirochaetes              --------------------------------MKKMIIIIILLLLMII----FV---NCT 21
82701_Desulfomonile              --------------------------------MKRTLILITLIGLVTT----------C- 17
468909_Gammaproteobacteria       ---MKNSSRGPLTLPPP-----VLR--KTLHALRTLPMLALLLLFAAQ----------V- 39
104369                           -------------MTTQ-----RLR--LDLW---AALLGLVLILAAMA----------A- 26
122072                           -----------MALMHT-----TLR--DVLW---SVLCGAVLLFHAGL----------A- 28
194837                           ---MKK---------------------VRGL-FYGGL--TGILLLATWAGVAGA---E-- 28
272093_Gammaproteobacteria       ---MKA--------SPN-----RSR--PRPHRHRQTP--AAWLLLALCALLA-------- 32
```

```
sp|Q1PW30.2|HDH_KUEST           VEIITHW--VPH---EVYGMPGEPDNSGKVFFSGLKAKYMGYPKDAQRSPYPGKYSKFWK 87
91837                           ------------------------------------------------------------ 21
sp|Q50925.2|HAO_NITEU           VP---------DETYDALKLDRGKATPK-ETYEALVKRY----KDPAHGAGKGTMGDYWE 74
SCZ85870.1                      VP---------DVTYEALGLDRSKATPK-ETHEALVKRY----KDPAQGAGKGTMGEYWE 74
28153                           ------------------------------------------------------------ 26
316357                          ------------------------------------------------------------ 17
91836_Desulfobacterales         ------------------------------------------------------------ 21
778484                          ------------------------------------------------------------ 20
237805_Desulfosarcina           ------------------------------------------------------------ 20
15066                           ------------------------------------------------------------ 22
628688                          ------------------------------------------------------------ 0
90566                           ------------------------------------------------------------ 303
648186_Desulfovermiculus        ---MTGSALAAT------------------------------------------------ 31
346596                          ------------------------------------------------------------ 0
247013                          ALAATVALGQSN------------------------------------------------ 26
474106                          -SGAVFGQESGN------------------------------------------------ 31
75139                           QEDTADPQAPGN------------------------------------------------ 37
823138                          ------------------------------------------------------------ 0
669580                          ---MMPAEAKEN------------------------------------------------ 9
141311                          ANAQKPAETAPN------------------------------------------------ 32
773104                          ------------------------------------------------------------ 0
778733_Desulfobacteraceae       ------------------------------------------------------------ 0
90565                           AA--SAFAQDAD------------------------------------------------ 30
```

```
63845                       ------------------------------------------------------------ 0
194073                      GA--VRAQTAQN------------------------------------------------ 36
523303                      VPEDRP------------------------------------------------------ 229
693944                      ------------------------------------------------------------ 0
234068                      AKTDTSYHRA-------------------------------------------------- 331
44033_Deltaproteobacteria   PGDGQRFGGVGAGGVDARGVDAGEWPRGNGTVEVTVT-----------RGGSGADGGSA- 395
591311                      ------------------------------------------------------------ 0
WP_015335051.1              --------AYAA---------------------------------------TEPFPN--- 31
WP_022853624.1              --------QVQA---------------------------------------KQEYTN--- 32
WP_007473950.1              ----------------------------------------------------SLDSN--- 27
WP_015902282.1              ----------------------------------------------------SLDSN--- 27
WP_002849252.1              --------V----------------------------------------------GN--- 23
WP_011992073.1              --------TDGN---------------------------------------KTEAIN--- 30
307368_Wenzhouxiangella     --------------------------------------------------------A--- 18
553040_Thialocapsa          ASAETSGGQQAA---------------------------------------GESLGD--- 50
WP_051227586.1              ---STSTVLAND---------------------------------------HSSSGD--- 36
160849                      ---TGHAADQAR---------------------------------------TGNVGD--- 35
595128_Anaerolineaceae      ------------------------------------------------------------ 33
94026                       ------------------------------------------------------------ 53
296856_Chloroflexi          ------------------------------------------------------------ 43
92136                       ------------------------------------------------------------ 0
167239                      ------------------------------------------------------------ 17
414796                      ------------------------------------------------------------ 14
```

```
547394                          ------------------------------------------------------------ 15
218326                          ------------------------------------------------------------ 16
328                             A----------------------------------------------------------- 19
87192_Ca._KSB1                  ------------------------------------------------------------ 14
289884_Ca._KSB1                 ------------------------------------------------------------ 14
66390                           Q-------GENP-------------------------------------------GP--- 33
238994                          ------------------------------------------------------------ 18
398289__Ca._GN15                ------------------------------------------------------------ 22
605530                          ------------------------------------------------------------ 0
722065_Spirochaetes             V-------DENE-------------------------------------------Q---- 27
82701_Desulfomonile             ---------PAL-------------------------------------------A---- 21
468909_Gammaproteobacteria      ---------HAV-------------------------------------------R---- 43
104369                          ---------EAA-------------------------------------------R---- 30
122072                          ---------EAA-------------------------------------------R---- 32
194837                          -------------------------------------------------------DK--- 30
272093_Gammaproteobacteria      ------------------------------------------------------------ 32
```

Hemo1

```
sp|Q1PW30.2|HDH_KUEST        TLPAYRYYIPDYMYNRDEVRPSNPIKGTFKLEQCVACHS----VMTPGIVRDYNKSAHSK 143
91837                        -----------------------GSSGFAATEICVGCHR----SVSPGLVADWEASSHSK 54
sp|Q50925.2|HAO_NITEU        PIAISIYMDPN-----TFYKPPVSPKEVAERKDCVECHS----DETPVWVRAWKRSTHAN 125
SCZ85870.1                   PIPYSMYLDPA-----TFYKPPTSMRDKASRKECVECHT----DESPVWVQAWKRSTHAN 125
28153                        -------------------FALENAPISEFTEECLMCHV----TATPGIVADWKRSRHYR 63
316357                       -------------------AGAQAPEPSEDTAVCIDCHR----DVHPGIVADWERSRHAR 54
91836_Desulfobacterales      -------------------ALASRLPISEDTKRCLECHR----SLHPGIVADWQKSRMAW 58
778484                       ---------------------AGEPPVSESTLVCIDCHN----LATPGIVAGWKKSTMAQ 55
237805_Desulfosarcina        -------------------VIGDDAPVSDATQECLDCHA----AIHPGVVSDWRKSRHAT 57
15066                        -------------------AAQGKPQLSSTSTTCQACHA----SVHPGIVEGWKKSLHSR 59
628688                       ------------------------VQQSTETEECLACHS----VLHPGLVESWKKSRHAQ 32
90566                        ---------DA-----SAET-----WTAEKTVACIECHR----NKTPAIYRDWSQSLHAR 340
648186_Desulfovermiculus     ----QLKEKEF-----RIER-----SMSKQAKACIMCHK----EEHPALFVDWANSRHAS 73
346596                       ------------------------------------------------------------ 0
247013                       ----FPKPKEF-----RIER-----SMPPQAVACIECHQ----RETPGLFTDWAGSRHAA 68
474106                       ----APKEKAF-----RIER-----SMPPEAVACIQCHK----QEHPGLFADWAASRHAS 73
75139                        ----MPKEKAF-----RVER-----SMSREAVTCLECHK----KEHPGIFADWAASRHAH 79
823138                       -------------------------------------------------FADWAASRHAS 11
669580                       ----MAKEKEF-----RIER-----SIPDAGVACIQCHK----REHPGIFADWANSGHAS 51
141311                       ----QAKEKAF-----RIER-----SMPKQAVACIQCHK----REHPGIFSDWANSRHAS 74
773104                       ------------------------------------------------------------ 0
```

778733_Desulfobacteraceae	--EHPGLFADWAQSRHAN	16
90565	----KEKVKAF-----RIER-----SMPDAAVACIECHK----REHPGLYTDWANSRHAS	72
63845	--	0
194073	----QAKEKTF-----RIER-----SMPKEAVACIECHK----RDHPGLFADWANSRHAS	78
**523303**	--------------------------AVAAAEGCIECHR----KKNPGLHADWASSTHAA	259
693944	----------F-----RTAAGAGIATVSDREAACIDCHQ----TEKPALTADWEKSVHAK	41
**234068**	---------------------------VSVADECADCHARDHENVLPGIYHDWERSIHAK	364
44033_Deltaproteobacteria	----SGAAEAG-----RAAGGI---GAGGGDSDCVACHW----EKKPALVEDWRLSIHGK	439
591311	--	0
WP_015335051.1	----LA-PKEL-----VVKR-----GFSKEATNCIECHA----KKTPGIVENWKMGKMAH	72
WP_022853624.1	----LA-GKKL-----VITR-----GYSKDAIKCIECHS----KKTPGIVQDWKMSRMAH	73
WP_007473950.1	----PNYVKLK-----NF-K-----PKGVSTDQCLMCHK----TTDPGIVADWQHSKHAK	68
WP_015902282.1	----PNYKKLK-----NF-K-----PQGVTNDQCLMCHK----AQDPGIVADWQHSKHAK	68
WP_002849252.1	----INLTKTM-----KVDR-----NLSPLAVKCIECHK----DKTPGIVNDWKSSRHAH	65
WP_011992073.1	----LNVIKNI-----KVAH-----KMSDLSKSCVECHS----EKTPGIVADWKNSRHAH	72
307368_Wenzhouxiangella	----ASWAQAE-----GEAP-----RMNSAEKGCVECHA----EVSPNTHSDWMSSAHYQ	60
553040_Thialocapsa	----LSQMQSI-----VIDR-----GLTPLGKSCVECHK----QHNPGIISDWKRSRHGH	92
WP_051227586.1	----LSQIKTI-----SFKR-----GLSDSDKACVSCHQ----QKQPGIVADWKDSRHSH	78
160849	----LSHVKSI-----TIDR-----NLTEEGAACVACHK----DKNPGIINDWKDSRHAH	77
595128_Anaerolineaceae	------------------EAEEEVNVLAGSDDECVVCHQ----RSTPGIIQQYGHSTMAA	71
94026	------------------AEPERVNVLANSDNECVVCHR----RTTPGIVEQYGHSTMAA	91
296856_Chloroflexi	-------A----------EEPERVNALANSEDECVACHR----RTTPGIVVQYGYSTMAA	82
92136	--	0

```
167239                             -----------------SLQPAAAQDEETVAGKCMTCHK----KKSPGLYQQWYRSNHGA 56
414796                             ------------------TGPALAVAADSPAGKCMTCHK----DKSPGLYKQWFTSKHGA 52
547394                             ------------------AAPVQAVTGDSPAGKCMTCHK----ETSRGLYHQWYNSKHGA 53
218326                             --------------------VVWAQTESTVAGRCMTCHK----EQSRGLYNQWYESSHAA 52
328                                --------------------TAQLPDEASVAGKCMTCHK----EKHPGLYQQWYESAHGI 55
87192_Ca._KSB1                     --------------------CLVTAAENNSAGRCMVCHK----EKTRGLYNQWFNSSHAM 50
289884_Ca._KSB1                    --------------------CALFSAENNAAGRCMVCHK----EQTRGLYNQWLNSAHAV 50
66390                              ------SVLSP-----DPAPSLEQTQQSTQAGRCLTCHK----EKSPGLYGQWLDSEHAR 78
238994                             --------------------LLMAQGGTQVAGKCMTCHK----EKSPGLYNQWYNSKHAL 54
398289__Ca._GN15                   --------------------GLLAAVDSEVAGRCMTCHK----EKSPGLYQQWFNSQHAV 58
605530                             ------------------------------------------------------------ 0
722065_Spirochaetes                -----------------LTFYTSKAVVSDRTNECLRCHT----KKQPSIINSWQGSAHAE 66
82701_Desulfomonile                -----------------IMKVDPKLYGSPEGQVCIKCHE----LKTPGLYKMWRSGRMGQ 60
468909_Gammaproteobacteria         -----------------PAGQPGVDWGDPKGQECVDCHM----SENPGLYWEWNHSQHGQ 82
104369                             -----------------PPGQAGTDWGDPAGQVCVDCHT----QENPGLFRQWNDSQHGQ 69
122072                             -----------------PDGKMGEDWGAPQGEKCVECHA----METPGLYWEWNDSQHGQ 71
194837                             ------PELLA-----EPEKETEPTWGHPAGEDCLKCHR----ENSPLLVAQWEDSPHAE 75
272093_Gammaproteobacteria         ------LPAAA-----EKVKLDPSDWGHPAGENCASCHE----KASPGIAEQWHQSAHKT 77
                                                                             Hemo2

sp|Q1PW30.2|HDH_KUEST              AEP------------------------------------APTGCDTCHGNN------H-- 159
91837                              ---------------------------------------EDVTCSTCHGEA------HK- 68
sp|Q50925.2|HAO_NITEU              LDKIRNLKSDDPLYYKKGKLEEVENNLRSMGKLGEKETLKEVGCIDCHVDV------NK- 178
```

645	SCZ85870.1	LDKVRKLQPSDPTYYKKAKLEEVEANLRSIGKLGEKEPLKEVGCIDCHVSI------NAD	179
646	28153	TTPSQALEAPE-----------LE-RRVSIKTPPERNKNVAVGCAECHMLN------PDD	105
647	316357	TRPLDSLAKPE-----------IE-RRLSAPTVPGELRDKTVGCAECHTLN------AEA	96
648	91836_Desulfobacterales	TTPKAAKSMIA-----------LK-RRVSFDNIPERLADVSVGCAECHTLN------PGG	100
649	778484	KTPEEARKEEE-----------KS-RRVSFDTLPDKLAGVVVGCAECHTLD------PGK	97
650	237805_Desulfosarcina	VTPQAAMAVDE-----------LQ-RRMSGRKTPEPLLATSVGCAECHTLR------GDA	99
651	15066	SSPAMGMAKPE-----------LE-RRISARNVPEALREVSVGCAECHTLN------PES	101
652	628688	TLPVEALQKDK-----------LE-RRISTETVDESLGNVTVGCYECHSLN------TDQ	74
653	90566	---------------------------------------ARVSCLHCHQGK------K-D	354
654	648186_Desulfovermiculus	---------------------------------------ANITCLDCHLAE------D-H	87
655	346596	--	0
656	247013	---------------------------------------ANISCLDCHMAE------E-H	82
657	474106	---------------------------------------ANITCYDCHKAE------S-F	87
658	75139	---------------------------------------ANITCYDCHRAE------E-H	93
659	823138	---------------------------------------ANITCLDCHQAE------P-T	25
660	669580	---------------------------------------ANITCIDCHKAE------E-H	65
661	141311	---------------------------------------ANITCYDCHKAD------E-R	88
662	773104	--	0
663	778733_Desulfobacteraceae	---------------------------------------ANITCFDCHKAD------E-T	30
664	90565	---------------------------------------ANITCYDCHVAE------S-F	86
665	63845	--	0
666	194073	---------------------------------------ANITCLDCHGAE------S-F	92
667	523303	---------------------------------------VGVNCATCHQTL------PK-	273

693944	---------------------------------------TGVTCLDCHEVL------DPK	56
234068	---------------------------------------VGVSCADCHRVN------DDA	379
44033_Deltaproteobacteria	---------------------------------------LGVACADCHKVA------GER	454
591311	--	0
WP_015335051.1	---------------------------------------ATVSCYDCHIVE------K-N	86
WP_022853624.1	---------------------------------------AGVSCYDCHVVP------K-G	87
WP_007473950.1	---------------------------------------AGVGCVECHVVP------K-D	82
WP_015902282.1	---------------------------------------VGVGCVECHVVP------K-N	82
WP_002849252.1	---------------------------------------AAVSCVDCHAVP------A-D	79
WP_011992073.1	---------------------------------------VGVSCMDCHSVA------A-D	86
307368_Wenzhouxiangella	---------------------------------------AGVTCLDCHRVK------P-D	74
553040_Thialocapsa	---------------------------------------VGVTCIDCHQVP------A-D	106
WP_051227586.1	---------------------------------------VGVGCNDCHQAQ------P-G	92
160849	---------------------------------------VGVSCIDCHEKP------A-D	91
595128_Anaerolineaceae	---------------------------------------AEVTCRDCHEVE------A-D	85
94026	---------------------------------------AEVTCQDCHEVN------A-D	105
296856_Chloroflexi	---------------------------------------AEVDCRDCHEVA------F-D	96
92136	--	0
167239	---------------------------------------AGVTCIQCHQAD------P-S	70
414796	---------------------------------------AGVTCIDCHGAE------D-G	66
547394	---------------------------------------AGVTCIECHGAN------E-G	67
218326	---------------------------------------HQVTCIDCHGAD------R-D	66
328	---------------------------------------HNVTCISCHKAD------E-D	69

```
87192_Ca._KSB1                       ---------------------------------------HNVTCIDCHRAK------K-N 64
289884_Ca._KSB1                      ---------------------------------------HHVTCLDCHQAA------K-K 64
66390                                ---------------------------------------HDVTCLDCHQAE------R-G 92
238994                               ---------------------------------------HKVTCIDCHQAD------R-D 68
398289__Ca._GN15                     ---------------------------------------HNVTCLDCHRAA------E-G 72
605530                               ------------------------------------------------------------ 0
722065_Spirochaetes                  ---------------------------------------AGVGCFECHQAE------L-G 80
82701_Desulfomonile                  ---------------------------------------AGVNCYDCHKAAKKTDKGP-A 80
468909_Gammaproteobacteria           ---------------------------------------NGVTCLDCHEAK------E-G 96
104369                               ---------------------------------------RGVNCFDCHRAA------P-G 83
122072                               ---------------------------------------VGVNCYDCHQSE------T-G 85
194837                               ---------------------------------------IGVNCMDCHQAG------Q-D 89
272093_Gammaproteobacteria           ---------------------------------------AGVNCMDCHQAD------A-A 91
```

Hemo3

```
sp|Q1PW30.2|HDH_KUEST          ----------------------------QKLTMPSSKACGTAECHETQYNEQGQGGIGSH 191
91837                          --GAR---------------------NANLAVLPDETVCK--ACHETQFNQFVKGKHNLG 103
sp|Q50925.2|HAO_NITEU          -KDKADHT--------------------KDIRMPTADTCG--TCHLREFAERESERDT-- 213
SCZ85870.1                     SKQKIDHS--------------------KDLVMPTADVCG--TCHLREFAERESERDT-- 215
28153                          HKDTFEHN------------------GRKVHTMVTPKDCQ--TCHPVEREQFSKNLMAHA 145
316357                         HDDAFEHS------------------GYTVHVVVSPEDCA--TCHPIEREQYAENIMSRA 136
91836_Desulfobacterales        HPDTVDHN------------------GHRIHIVVTPEDCA--VCHPQERRQYGKNIMSHA 140
778484                         HKDSFDHS------------------GFQVHTVVSPGDCA--TCHPVEAEQFKENLMSEA 137
237805_Desulfosarcina          HADTFEHN------------------GYDIHIVVSPDDCA--TCHTIEREQYAQNIMAMA 139
15066                          HADTFNHN------------------GFQVHTVVTPEDCA--VCHPQEREQFTRNIMSQA 141
628688                         HQDAFEHN------------------GYVINVVVSPNDCA--TCHPVEREQYSKNLMAFA 114
90566                          DPDISVDHAEYY-KDNGPSYAVTE-YRTPVSALVSPRDCS--RCHPREVKQFSESKHAHA 410
648186_Desulfovermiculus       DPDVSQSHFEQY-QRDDTKWGASE-YRVPIATVVTPKDCS--RCHPDEVTQYEKSKHANT 143
346596                         ------------------------------------------------------------ 0
247013                         DPDVAKDHFQYY-ERADLPYGRQE-YKIPVAGVVTPKDCS--RCHPDEVTQYNRSKHANT 138
474106                         DPDVSKSHEKEY-QSSTSKYGTRE-YMVPVSAVVTPKDCS--RCHPDEAKQYSKSKHANT 143
75139                          DPDVSKGHYQYY-DEYVRKFGPKDFYKVPVSGVVTPKDCS--RCHPDEAKQYSVSKHANT 150
823138                         DPDVSKSHNQYY-DEYRRKYGPKEFYDVPIAAAVTPKDCS--RCHPDEAKEYARSKHANT 82
669580                         DPDVAKDHYQQY-EKSDTEWGRPE-YRVPVAGVVTPKDCS--RCHPDEAKQYSKSKHANT 121
141311                         DPDVSQSHFKQY-ERSDLKYGTRE-YQVPVAAVVTPKDCS--RCHPDEAMQYNKSKHANT 144
```

```
773104                      ------------------------------------------HPDEAKQYGRSKHANT 16
778733_Desulfobacteraceae   DPDVSQEHFKQY-QRVDQPYGTSE-YKIPIAAVVTPKDCS--RCHPDEAKQYSQSKHANT 86
90565                       DPDVSESHYQQY-QRSDLKYGTEA-YRVPVAAVVSPKDCS--RCHPDEAKQYSVSKHANT 142
63845                       ---------------------------------------------------------- 0
194073                      DPDVSQSHFKQY-ERSDLKYGTKE-YSTPISAVVTPKDCS--RCHPDEAKQYSVSKHANT 148
523303                      SPLVHQAHLKH--------------DPTPIRVVVTPRTCA--ACHPEEAAQYDRSKHAHT 317
693944                      TGWTSAAHSEY--------------SEVPVSPLVTPKRCA--MCHQEEVDQYGASKHANT 100
234068                      DVTISERHLAN--------------SPIPISALVTPKRCR--GCHPDSAAEYARSKHAHT 423
44033_Deltaproteobacteria   DVWVSKSHYEY--------------TDVPVTPLVTPRRCA--RCHQKEVDQYARSKHAHT 498
591311                      --------FEY--------------SRVAISGLVSPARCA--MCHEQEVEEYGRSKHANT 36
WP_015335051.1              SPMASQCEGLKGT-------------GLFISPMVSSKTCS--RCHPQEVDQFLKSGHARL 131
WP_022853624.1              SPMASQCEGVKGT-------------NIFTSPMVSPKTCA--KCHPSEVEQFTKSAHAAM 132
WP_007473950.1              YPTAFKSHPMQGA-------------NWTVQIAVSSVTCA--KCHAKEVTEFLNSGHARG 127
WP_015902282.1              YPTAFKAHPMQGS-------------NWTVQIAVSSVTCA--KCHAKEVTEYMNSGHARG 127
WP_002849252.1              SPMAIKKEHPKDS-------------GNHVSILVSPKTCA--KCHAKEVEQFQQSGHARG 124
WP_011992073.1              SPMASVKVHPKDS-------------NNHVSMLVSPKTCA--KCHENEVDEFTKSGHARG 131
307368_Wenzhouxiangella     SETAIQHEDVFDMSGYDTSLL--D-ERIHISVLVPPSTCA--KCHDAEHQQFTEGGHYRS 129
553040_Thialocapsa          APNATQHESLVGT-------------DTYISVLVSPDTCG--RCHPREQEQFNRSGHFRG 151
WP_051227586.1              QPGAQLHNKGELK-------------NAYITPLVSPATCG--RCHAEEQEQFNRSGHFRS 137
160849                      SPMAYQCEGIEGT-------------DTYISALVPPSTCG--RCHAQEHDQFNASGHFRS 136
595128_Anaerolineaceae      YPAAIEHE------------------GTHVLNTPTTAMCE--DCHQAQVAQFNQSRHALP 125
94026                       YPGSVEHE------------------GTYVLQSPTTAMCE--QCHTAEVAQFNQSRHGIP 145
296856_Chloroflexi          YPGSVEHE------------------GTSVLNQPSTAMCE--KCHTAEVAQFNQSRHGIP 136
```

```
92136                               ------------------------------------------------------------ 0
167239                              EPDAYEHE------------------GAYIATLVTPKDCG--ACHEEEAKQVQNSYHAKA 110
414796                              EPDGYMHE------------------GALIATLVTPEDCG--SCHEQVAEEVMNSYHAHA 106
547394                              EPDAFEHE------------------GALIATLVTPKDCG--TCHQKEADQVQNSYHAHA 107
218326                              DPDAFEHY------------------DAWIATMVTPKDCG--ECHPKESEQMSGSYHARA 106
328                                 DPDAYEHY------------------DATIATLVTPKDCG--RCHEKEAEQVSNSYHAKA 109
87192_Ca._KSB1                      EKDAFLHY------------------DSYIATLVTPKDCG--TCHPAEMKEVSGSHHARA 104
289884_Ca._KSB1                     EKDAFLHY------------------DQYIATLVTPKDCG--SCHPTEAEEVSGSHHAKA 104
66390                               EPDAFMHE------------------GARIATLVTPRDCG--ECHEEETAQVEASYHANA 132
238994                              EPDAFMHE------------------GAYIATLVTPDDCG--TCHAGETEEVNNSYHASA 108
398289__Ca._GN15                    EPDGFMHY------------------DARIATLVTPKDCG--TCHPTESKQVSESYHASA 112
605530                              -----------------------------------PRDCA--KCHEKEVDEFKNSHHAKA 23
722065_Spirochaetes                 EADAFEHF------------------DSIIATLVTPKDCS--QCHQAEAEQFLDSHHAKG 120
82701_Desulfomonile                 DVDGHDHR------------------GFWIVTLVSPKDCS--RCHKEQFEQQQPSHHAKG 120
468909_Gammaproteobacteria          EIDAWRHE------------------DTYVSIVVTPKDCS--KCHEKEFEEMDGSHHAKG 136
104369                              DKDGFMHH------------------GERISVIVSPQDCA--RCHQNEYEQMDGSHHSKG 123
122072                              DADGFMHH------------------EERITIVVSPKDCA--QCHETEFEEMDGSHHAKG 125
194837                              DPDAITHH------------------GQTVSTLVSPLDCG--RCHEQEYNQHRGSTHAKA 129
272093_Gammaproteobacteria          DVDAIEHE------------------GHVIATIVSPKDCG--RCHEKEYREQEGSVHAEA 131

sp|Q1PW30.2|HDH_KUEST               ASCSS---------------------------------------------FAQVECAWSI 206
91837                               WTAMNA-LPV-------THV-------------------------E-------------P 117
sp|Q50925.2|HAO_NITEU               --MVWP-NGQWPAGRPSHALDYTANI---------------------------ETTVWAA 243
```

```
SCZ85870.1                         --MIWP-NGQWPDGRPSHALDYSANV---------------------------ETTVWAA 245
28153                              RGNLTK---N----KVYMSLVESVNGT-----QELEDS-KLATKII-------------P 179
316357                             HGNLVD---N----PVYNDLAATTNGV-----PVIEDD-AAEPGLL-------------N 170
91836_Desulfobacterales            YGNLKH---N----TIYADLVTQVNGV-----HTFNDG-TITV--G-------------E 172
778484                             YVNLEN---N----ALYQDLITSVNGL-----QSFENG-KLVS--S-------------E 169
237805_Desulfosarcina              EKNLSA---N----SLYEDLERHISGT-----PEIKPD-RLHF--S-------------P 171
15066                              RGNLID---N----PVFSDLQQSIIGP-----PTLEQA-GLRF--G-------------E 173
628688                             HSNLVD---N----ALYQDFMKTINNP-----YTFQDQ-ELVL--G-------------E 146
90566                              VDIMWK-TDP-----------------------WLRDK---------------------M 425
648186_Desulfovermiculus           LELIWK-IDP-----------------------WLNDG---------------------M 158
346596                             ------------------------------------------------------------ 0
247013                             IEIIWK-IDP-----------------------WLNDG---------------------M 153
474106                             IEIIWK-IDP-----------------------WLNDG---------------------M 158
75139                              IEIIWK-IDP-----------------------WLNHG---------------------M 165
823138                             MEIIWK-IDP-----------------------WLNHG---------------------M 97
669580                             LEIIWK-IDP-----------------------WLNDG---------------------M 136
141311                             LEIIWK-IDP-----------------------WLNHG---------------------M 159
773104                             LELIWK-IDP-----------------------WLNDG---------------------M 31
778733_Desulfobacteraceae          MEIIWK-IDP-----------------------WLNKG---------------------M 101
90565                              LEIIWK-IDP-----------------------WLNDG---------------------M 157
63845                              -EIIWK-IDP-----------------------WLNDG---------------------M 14
194073                             IEIIWK-IDP-----------------------WLNDG---------------------M 163
523303                             REIMWK-VDP-----------------------WLNFG---------------------M 332
```

798	693944	IKIINT-VDN-----------------------WLIHD---------------------M	115
799	234068	LEIINQ-IDK-----------------------WLIYG---------------------M	438
800	44033_Deltaproteobacteria	LEIINR-IDN-----------------------WLIHG---------------------M	513
801	591311	LQIINT-IDK-----------------------WLIHG---------------------M	51
802	WP_015335051.1	SGVPVIESKK------FIKLMYYYEGA-----------------------EFIGVKAGSG	162
803	WP_022853624.1	ASRPVL--TK------FVKLWRDLEGG-----------------------VFAGMDPKSK	161
804	WP_007473950.1	AAQWL-ATPKNPHGIAMTRLAYGYETLRGNHPKYLADGKTLTKGIRKDDKFFRANEKNPR	186
805	WP_015902282.1	AAQWL-ATPKNKHGYLMTKLSYHYESLKGANQSYMVNGKKMEKGIRTDGPVFQANEKSPR	186
806	WP_002849252.1	GVQMF-AKKG------MVELMYHYEAN---GKP-----------YENDAPFYAGNE---N	160
807	WP_011992073.1	AMQMY-ANPA------IVKLMYHYEGA---DHP--------------------------D	155
808	307368_Wenzhouxiangella	YHQII-PKDN------LHALTQVHEGQ---NHP--------------------------E	153
809	553040_Thialocapsa	YRQQI-PKDD------LHALVHRHEGR---GHP--------------------------Q	175
810	WP_051227586.1	YRQII-PKDS------LHALVRVHEGR---NNK--------------------------E	161
811	160849	YRQTI-PKDS------LHALVNRHEGQ---NHP--------------------------E	160
812	595128_Anaerolineaceae	AYVAYA-GQE----ALSEELLAQYQAIPEGG--YLPE------------VTRARNSLHAL	166
813	94026	AYVAMV-GTE----SLSPELLSAYESIPEAQ----PE------------PNEARNALFRL	184
814	296856_Chloroflexi	AYVAMV-GTE----SLSEELLAQYQQIPEAQ----PK------------PNEARNALYEL	175
815	92136	---AMV-GLE----GLEPPMQALYQTIPEAQ----PK------------PNEARNALYAL	36
816	167239	GEILQS-KDA----YLAHAV-GG--------------------------D----------	128
817	414796	GEILDS-KDA----YLAHAA-GG--------------------------H----------	124
818	547394	GEILDS-KDA----YLAHAA-GG--------------------------H----------	125
819	218326	GEILES-NDA----YLAHTS-AG--------------------------L----------	124
820	328	GQILDS-ADA----YLAHVG-AG--------------------------H----------	127

```
87192_Ca._KSB1                  GQILES-NDA----YLAHVA-AG--------------------------Y---------- 122
289884_Ca._KSB1                 GQILKS-NDA----YLAHVA-AG--------------------------Y---------- 122
66390                           GQILES-ADA----YLAHAV-GG--------------------------E---------- 150
238994                          GLILES-TDA----YLAHVS-AG--------------------------E---------- 126
398289__Ca._GN15                GKILES-KDA----YLAHVT-AG--------------------------K---------- 130
605530                          GRILGS-LDN----VLAEVV-EG--------------------------NKGLKTMAFPN 51
722065_Spirochaetes             GEILNS-LDN----YLGEVV-EG--------------------------P---------- 138
82701_Desulfomonile             ADILNS-ADN----YLGTVV-GG--------------------------K---------- 138
468909_Gammaproteobacteria      GQILGS-LDN----LLGEVV-GG--------------------------P---------- 154
104369                          GQILAS-LDN----LLGEVV-GG--------------------------P---------- 141
122072                          GQILAS-LDN----LLGEVI-GG--------------------------P---------- 143
194837                          ARRSKA-WSE----VLVHRL-SG--------------------------E---------- 147
272093_Gammaproteobacteria      YAIIKD-RVP----ALAHNV-TG--------------------------P---------- 149
```

P460

```
sp|Q1PW30.2|HDH_KUEST          ERPPGDTAGCTFCHTSP--------------------------------------EERCS 228
91837                          DELMEGGRGCGGCHNMGIKTEAQREEQRK-----------------KG---YRYQNNSCD 157
sp|Q50925.2|HAO_NITEU          MPQREVAEGCTMCHTN---------------------------------------QNKCD 264
SCZ85870.1                     MPQREVAEGCTMCHTN---------------------------------------QNKCD 266
28153                          ANKMTDADSCFHCHGTEVKVIGKETRDTEYGELEFPK-FEGWPNQGVGRVNTDGSKGSCS 238
316357                         PDALTEEASCLYCHGTRVEVRELAPRETDYGEMSFPV-LSGWPNQGVGRINPDGSLGSCT 229
91836_Desulfobacterales        PDSETLANSCFYCHGTVVEVTGKETRDTVEGPLTFPV-LSGWPNQGVGRINPDGSKGSCS 231
778484                         PDAETDAESCFSCHGTAVEVKGAETRTTSMGDMRFPV-LSGWPNQGVGRINPDGVKGSCT 228
237805_Desulfosarcina          PNDMTQADACYYCHGTRLQVTGSEVRDTVAGELEFPV-IANWPNQGVGRVNLDGSRGACS 230
15066                          PDRLTTEEACLYCHGTKIEVQGQEQRKTVMGPMTFPV-LKGWPNQGVGRLNPDGSLGSCS 232
628688                         HDPLTANESCLYCHGTKVKVTGKTIKETDFGELEFPV-LSGWPNQGVGRINPDESRGACT 205
90566                          VSKVEKNTGCDICHGSVVEA-------SN----GQLD-PSAWPNTGVGRINPDGSRGSCS 473
648186_Desulfovermiculus       NSDWERSAGCFYCHGTVLKQD------AE----GKLD-PMTWPNVGVGRVNLDGSRGSCT 207
346596                         --------GCYACHGTVLKM-------KD----GAID-PDTWPNVGVGRVNLDGSLGSCT 40
247013                         NSDTERKSGCFYCHGTVLEM-------KE----GKLS-SETWPNVGVGRVNLDNSLGSCT 201
474106                         NSETERISGCYHCHGTIIET-------KD----GALD-PDTWPNVGVGRVNPDGSKGSCT 206
75139                          NSDFERASGCFHCHGTVLEM-------KE----GELD-PDTWPNTGVGRVNLDGSLGSCT 213
823138                         NSDFERASGCYHCHGTVLKM-------KD----GTLD-PETWPNVGVGRVNLDGSKGSCT 145
669580                         NSDIERASGCYYCHGSVLKT-------ED----GNLT-SETWPNVGVGRLNLDGSKGSCT 184
141311                         NSDLERASGCFHCHGTVLKQ-------SD----GRLD-PETWPNVGVGRVNLDGSKGSCT 207
773104                         NSDFERATGCFYCHGTVLKQ-------VD----GKLD-PKTWPNVGVGRINLDGSKGSCT 79
```

860	778733_Desulfobacteraceae	NSDFERASGCYHCHGTVLKM-------KD----GKLD-PLTWPNVGVGRINLDGSKGSCT	149
861	90565	NSDFERASGCYYCHGSIVKK-------KD----GRLD-EQTWPNVGVGRINLDGSKGSCT	205
862	63845	NSDFERASGCYHCHGTVVKM-------AD----GKLD-PLTWPNVGVGRINLDGSKGSCT	62
863	194073	NSDFERASGCFHCHGTVLKQ-------KD----GALD-PETWPNVGVGRLNLDGSKGSCT	211
864	523303	NSELERTSGCYACHGTQVKL-------EE----GRPV-QGTWPNVGVGRMNPDGSLGSCS	380
865	693944	NNAAERTTGCYACHGSKIEI-------IN----GRPA-AGTWPNVGVGRINPDGSLGSCT	163
866	234068	NNATERSTGCYACHGSVVEF-------AR----GQPV-AGTWPNVGVGRVNPDGSKGSCT	486
867	44033_Deltaproteobacteria	NSSIERATGCFACHGSVVEI-------ED----GEPV-PGTWPNVGVGRKNPDGSLGSCT	561
868	591311	NNETERATGCFACHGTTVVF-------ED----GVPV-EGSWPNVGVGRKNPDGTLGSCT	99
869	WP_015335051.1	TSMASRASGCQMCHGTQVELG------PD----NKPI-NNTWPG-GVGTRYPDGSIGTCT	210
870	WP_022853624.1	LTTAPRQSGCQACHGAEVKLG------PD----NKPT-KDSWPG-GIGHRYPDGGIGNCV	209
871	WP_007473950.1	LSDLSVANICIQCHGTIIKLD------KN----GKPD-AATWPNDGIASLYPDGGVGNCL	235
872	WP_015902282.1	VADLNVANICIQCHGTTIKLD------KN----GRPD-ATTWPNDGIAALYPDGGVSNCL	235
873	WP_002849252.1	LKDAPASTGCIQCHGMEIKLD------KE----GYPLPGKGWPNYGIGNAYPDGSVGSCK	210
874	WP_011992073.1	FKMAPDATGCTQCHGTVIKLD------AD----HKPT-KETWPNYGIGNVYPDGGIGGCK	204
875	307368_Wenzhouxiangella	LAGAPNETGCMQCHGTEIELD------ED----GRPT-AETWPNAGMGNIYPNGSTGNCT	202
876	553040_Thialocapsa	LSAAPDMTGCMQCHGTEIKLD------DD----GRPT-ARTWPNSGIGSLYPDGSTGNCT	224
877	WP_051227586.1	LGNAPDETGCMQCHGTKIKLD------EK----GKPD-PTTWPNMGMGNIYPDGSTGNCA	210
878	160849	LHGAPGETGCMQCHGTEIKLD------ED----NRPT-PETWPNAGMGNIYPDGSTGDCD	209
879	595128_Anaerolineaceae	EGPAVTHFACESCH-------------------------------NIGKPAPDGSVGQCQ	195
880	94026	EGPDVTKFACESCH-------------------------------NIGKPAEDGSIGQCE	213
881	296856_Chloroflexi	EGPDVTRFACESCH-------------------------------NIGKPHEDGSIGKCE	204
882	92136	EGPAVTRFACEGCH-------------------------------NVGRPHEDGSVGECQ	65

```
167239                          ---PVAIAGCESCHGAKVEIDPE----RD----NKLS-RLSWPNSGIGRINPDGSKGACT 176
414796                          ---PVAIAGCESCHGAKMVIDEN----SP----NKLS-RLSWPNSGIGRINPDGSKGSCT 172
547394                          ---PVAVAGCESCHGARIRIDPE----SP----NKLS-RLSWPNSGIGRLNPDGSKGSCT 173
218326                          ---PVAITGCESCHGAKIEVDEA----RE----NKLS-MESWPNNGIGRIHPDGSRGSCN 172
328                             ---PAAIQGCESCHGAKMEIDPE----SP----NKLS-KTSWPNSGIGRINPDGSLGSCN 175
87192_Ca._KSB1                  ---PVAIQGCESCHGANVVIDSK----AE----NKLA-RTTWPNSGIGRINPDGSLGSCN 170
289884_Ca._KSB1                 ---PVAIQGCESCHGANVVIDSQ----AE----NKLS-RASWPNSGIGRINPDGSLGSCN 170
66390                           ---PAAIAGCESCHGTNVQIDPD----SP----NRLA-RESWPNSGIGRINPDGSKGSCN 198
238994                          ---PVEILGCESCHGARIEIDES----RE----NKLS-VKSWPNSGIGRINPDGSKGSCN 174
398289__Ca._GN15                ---PVAILGCESCHGARMEIDPD----QP----NKLA-KECWPNSGIGRINPDGSLGSCN 178
605530                          GTSAAAVNGCWQCHGSEIKVH------DD----GSLD-LATWPNTGIGRINPDGSEGSCT 100
722065_Spirochaetes             ---AASVSGCQQCHGSQVNVT------ED----GKLS-PDTWPNFGIGRINPDGSAGACS 184
82701_Desulfomonile             ---ACVSTGCRQCHGSNIKVL------KD----GKLD-PATWPNTGIGRINPDGSKGSCS 184
468909_Gammaproteobacteria      ---AAVNAGCKQCHGSKLEFITEGSRRDK----GRPK-PGTWPNTGIGRINPDGSLGSCT 206
104369                          ---AAVHAGCWQCHGAEVKVG------PD----GKPS-IETWPNTGIGRINPDGSKGSCT 187
122072                          ---AAVNAGCRQCHGAEIKIE------ED----GKPS-LETWPNTGIGRVNPDGSLGSCT 189
194837                          ---IMQDIGCERCHGGEVKVLE-----NQ----GGLD-LNTWPDHGIGRLNPDDSRGNCS 194
272093_Gammaproteobacteria      ---EMQAAGCDQCHGSQVKVR------GD----GTLD-PATWPNSGIGRINPDGSKGSCS 195
                                         *  **                                            *


                                Hemo5             Hemo6
sp|Q1PW30.2|HDH_KUEST           TCHQRHQFDPAVARRSEQCKTCHWGKDHRDWEAYDIGLHGTVYQVN-----KWDTEQFDF 283
91837                           ECHTRHAFSVREARDPRACRQCHMGYDHPQWEMWVSSKHGTRYTVK-E---NG------- 206
```

sp|Q50925.2|HAO_NITEU	NCHTRHEFSAAESRKPEACATCHSGVDHNNWEAYTMSKHGKLAEMN-RDKWNWEVRLKD-	322
SCZ85870.1	SCHTRHEFSAAESRKPEACATCHSGVDHNNWETYSMSKHGKMVAML-GDKWNWEAPLKD-	324
28153	ACHSRHEFSIEMARKPYTCGQCHKGPDVPAYKAYEVSKHGNLFSAM-K--SGWKFNEVP-	294
316357	ACHTRHQFSIEMARKPHTCSQCHKGPDVPGYKVYNVSKHGNLYSAH-K--KEWSFDPVP-	285
91836_Desulfobacterales	ACHTRHQFAIQMARKPATCSECHKGPDVPGYKVYEVSKHGNIYASL-GEEKTWDFKAVP-	289
778484	ACHPRHRFTIETARKPHTCSQCHKGPDVPAYKVYSVSKHGNIYSSV-G--KDWDYNKIP-	284
237805_Desulfosarcina	ACHTRHRFSIEMARKPYTCKECHVGPDVPAFKVYSASKHGNIFASM-N--HEWEFNTVP-	286
15066	ACHTRHDFSIVTARKPYTCKECHIGPDVPAYKVYSTSKHGNIFSTH-E--SAWDFEAVP-	288
628688	SCHPRHTFSIETARKPYTCSECHKGPDVPAYKVYVVSKHGNIFKSK-E--KEFEFNSVP-	261
90566	SCHSRHRFSIMEARKPESCGRCHVGPDHPQKEIFMESKHGDIYKAF-GDQYQWTTAANA-	531
648186_Desulfovermiculus	SCHTRHRFSVMEARKPEACGQCHLGPDHPQIEIYEESKHGDIYHAF-GDEYNWDSAPGT-	265
346596	SCHTRHRFSVAEARKPEACDQCHLGPDHPQIEIYNESKHGTMYHAY-QDEYNWDAAPGT-	98
247013	SCHTRHRFSVMEARKPEACGQCHLGPDHPQIEIYMESKHGDIYTAF-GDDYEWKAAPGA-	259
474106	SCHTRHRFSVMEARKPEACGQCHLGPDHPQIEIYMESKHGDIYTAF-GDEYNWTAAPGT-	264
75139	SCHTRHRFSVMDARKPEACGQCHLGPDHPQIEIFTESKHGDIYDAF-GDEYNWTAAPGT-	271
823138	SCHTRHRFSVMEARKPEACGQCHLGPDHPQIEIFMESKHGDIYTAF-GDEYNWTAAPGT-	203
669580	SCHTRHRFSIMEARKPEACGQCHLGPDHPQIEIYMESKHGDIYTAF-GDDYNWESAPGT-	242
141311	SCHTRHRFSVMEARKPEACGQCHLGPDHPQIEIYMESKHGDIYTAF-GDQYNWEAAPGT-	265
773104	SCHTRHRFSVMEARKPEACGQCHLGPDHPQIEIYMESKHGDIYTAF-GDEYNWKSAPGT-	137
778733_Desulfobacteraceae	SCHTRHLFSVMEARKPEACGQCHLGPDHPQIEIYMESKHGDIYTAH-GDNYNWTAAPGT-	207
90565	SCHTRHRFSVMDARKPEACGQCHLGPDHPQIEIYMESKHGDIYNAF-GHEYNWTAAPGT-	263
63845	SCHTRHRFSVMEARKPEACGQCHLGPDHPQIEIYMESKHGDIYTAF-GDQYNWDAAPGT-	120
194073	SCHTRHRFSVMEARKPEACGQCHLGPDHPQIEIYMESKHGDIYTAF-GDDYNWNAAPGT-	269

929	523303	SCHTRHRFSKAEARKPEACDQCHLGPDHPQIEIYNESKHGTLYHAE-GDEWTWLPEDGK-	438
930	693944	SCHTRHRFSVVEARKPEACDQCHLGPDHPQIEIYNESKHGTMYHAY-GDEWTWRPEDFH-	221
931	234068	SCHTRHRFSIEEARKPEACDQCHLGPDHPQIEIYNESKHGTIYHAE-GAEWQWRPNDGQ-	544
932	44033_Deltaproteobacteria	SCHTRHRFSVVEARKPEACDQCHLGPDHPQIEIYNESKHGTMYHAY-GDEWTWRPEDFH-	619
933	591311	SCHTRHSFSIEEARKPEACDQCHLGPDHPQIEIYNESKHGTIYHAE-GDSWNWRPEDFT-	157
934	WP_015335051.1	VCHTRHMFSIKEARKPEACASCHLGPDHPQAEIYEESKHGQIFAAH-GEDWKWDSAPDT-	268
935	WP_022853624.1	VCHNRHKFSVAEARKPEACGKCHLGPDHPNIEIYYESAHGQRYLTE-GEEWKWDAAPDA-	267
936	WP_007473950.1	SCHSRHKFSAAESRHPMACSNCHLGPDHPDKEIFESSVHGHIFDTN-EEDYNFKT--GE-	291
937	WP_015902282.1	SCHSRHKFSAAEARQPGACTNCHLGPDHPMKEVFESSVHGHIFETN-EEDYKFDT--GE-	291
938	WP_002849252.1	SCHSSHKFDMTEARKPSACASCHLGPDHPNIEIYNNSMHGKIFNAE-GNTWKWDSAPDT-	268
939	WP_011992073.1	SCHSSHTFNIAEARKPAACASCHLGPDHPDIEIFNNSMHGHIFNAE-GNTWKYDSAPDT-	262
940	307368_Wenzhouxiangella	ACHSRHRFSVAEARQPFACAECHLGPDHPNIEIFEASKHGHVFNTRNDDEWNWDAEGGD-	261
941	553040_Thialocapsa	TCHTRHRFSLAEARHPAACASCHLGPDHPDIEVYENSKHGQIYAVE-GDDWRWDSAPDA-	282
942	WP_051227586.1	ACHTRHKFSIAEARKPAACASCHLGPDHPDIEVYNNSKHGHIFNAD-GYNWKWDSPPDG-	268
943	160849	SCHTRHAFTIAEARHPRACASCHLGPDHPNIEIYENSKHGQVFLAE-GHQWKWDSAPDA-	267
944	595128_Anaerolineaceae	DCHLRHEFSLEQARKPETCNYCHIGPDHPQWEIYQESPHGTAYLTD-GHNWNWEA--EP-	251
945	94026	KCHLRHMFSLEQARKPETCNACHIGPDHPQWEIYHESPHGIAYATS-GHTWNWEA--EA-	269
946	296856_Chloroflexi	KCHLRHVFSLEQARKPETCNACHIGPDHPQWEIYHESPHGIAYATD-GHRWHWEA--ET-	260
947	92136	KCHLRHEFSLEQARKPETCNACHIGPDHPQWEIYHESPHGIAYATG-GHRWHWEA--EP-	121
948	167239	ACHTRHNFDVAQARKPEACSKCHLGPDHPQKEVYEASKHGNVYFTN-QDVMNLRS--DR-	232
949	414796	ACHTRHSFDVAQARKPEACSKCHLGPDHPQKEVYEASKHGNTYFTN-VEAMNLDS--DA-	228
950	547394	ACHTRHNFDVAQARKPESCSKCHLGPDHPQKEVYEESKHGNAYYTN-QDKMNLDS--DR-	229
951	218326	ACHVRHLFSRAQARQPESCSKCHLGPDHPQKEVYEESKHGNTYYTN-RERMNLKA--DR-	228

```
328                          ACHTRHSFDIAQSRQPQSCGKCHLGPDHPQMEIYEESKHGNTYYTN-REKMNLDA--QE- 231
87192_Ca._KSB1               ACHTRHAFSRAQARQPESCSKCHLGPDHPQKEVYEESKHGNAYYTN-IGQMNLEA--DP- 226
289884_Ca._KSB1              ACHTRHAFSKAQARQPESCSKCHLGPDHPQKEVYEESKHGNAYYTH-IDEMNLSA--DP- 226
66390                        ACHTRHSFSKRQARQPESCSKCHLGPDHPQKEVYEESKHGNTYYTN-TDKMNLTS--DR- 254
238994                       ACHTRHSFAKSQARQPESCSKCHLGPDHPQREIYEESKHGNTYYTN-IDKMNLDS--DR- 230
398289__Ca._GN15             ACHTRHAFSIGQARQPEACSKCHLGPDHPQKEVYEESKHGNTYYTN-TDKMNLDS--DS- 234
605530                       ACHSRHEFSAAQARHPENCGKCHMGPDHPQIEIYNESKHGVAFRAN-VNKMNIDS--PK- 156
722065_Spirochaetes          ACHSRHDFSLKQARTPETCSKCHLGPDHPQKEVYEESKHYIAYNSH-RDEMNMDE--EK- 240
82701_Desulfomonile          ACHTRHRFSVEQARRPDACGKCHLGPDHPQKEVYEESKHGILYQAF-RDKLNMKG--RK- 240
468909_Gammaproteobacteria   ACHGRHRFSKAQARTPDTCGKCHVGPDHPQIEVYNESKHGIIYRAK-VDEMNLES--DK- 262
104369                       ACHGRHGFSKAQARTPDTCGKCHLGPDHPQLEVYNESKHGIIYRAR-VEDMNLDS--DK- 243
122072                       ACHGRHGFSRAQARTPDTCGKCHVGPDHPQIEVYNESKHGIIYRAM-KDKMNLDS--DK- 245
194837                       ACHARHRFSKVQARAPETCAKCHGATDAPNWGIYISSSHGRHFQLF-REHLKLSG--EE- 250
272093_Gammaproteobacteria   SCHGRHSFSKAQAREPSACVRCHSGPDSPDKEIFEASKHGMIYAAQ-RDAMNLHA--DT- 251
                              **  * *    :*    *  **  . *      :  . *
```

```
                                                   Hemo7
sp|Q1PW30.2|HDH_KUEST         SKKLSDADY-VGPTCQYCHMRGGHH-----------NVQR-------------------- 311
91837                         ---KLPEG-AAAPSCQFCHMPDGDH-------------ENHTAWGFLGVRLPLPED---- 245
sp|Q50925.2|HAO_NITEU         -AFSKG-G-QNAPTCAACHMEYEGE--------YTHNITRKTRWANYPFVPGIAENIT-- 369
SCZ85870.1                    -AYAVG-G-QNAPTCAGCHFEYEGE--------YTHNITRKIRWANYPFVPGIAENIT-- 371
28153                         --WTVGTDF-SGPTCAVCHVSLLVTEEGDTIAKRSHQMSDRLPWRILGLIYAHPHTKSPD 351
316357                        --WVLGRDF-SAPTCAVCHVSLTVDEYGTVIAERSHQMSNRLAVRLFGL-YSHPHPESPD 341
91836_Desulfobacterales       --WTVGEDF-SAPTCAVCHVSLVVSGDGEVVAERTHRMNDRLGWRIMGLIYAHPQPKSPD 346
778484                        --WTVGRDF-TAPTCAACHISLVVSPEGEVIAERTHRMNDRIPWRLFGLIYSHPHPKSPD 341
237805_Desulfosarcina         --WVIGEDF-GAPTCATCHISLTVNTDGEVINRRSHQVSDRLGWRIFGLIYAHPQPKSPD 343
15066                         --WKIGEDF-TAPTCAACHMSLTVTPQGKVVAQRTHQVNNRLATRLFGLIYAHAHPKDPD 345
628688                        --WVIGKDF-TTPTCATCHVSLLVTEDETVIAERTHQFNNRLAWRLFGVPYAHPHPVSAD 318
90566                         --WTAGTDF-RGPTCAVCHMSHTET------ASVSHNVSERLAWESQSPLSV-RPSEFE- 580
648186_Desulfovermiculus      --WTPGVDF-RGPTCASCHMSGAGE------VMTTHDVTERLSWEIQAPLTV-RPSEFK- 314
346596                        --WTPGLDY-RSPTCASCHMSGAGQ------VKTTHDVTERLSWETQAPLTV-RPSEFK- 147
247013                        --WTPGVDY-RAPTCASCHMSGVGE------VATSHDVTERLSWETQAPLTI-RPSEFS- 308
474106                        --WTPGIDY-RGPTCASCHMSGAGE------TLTTHDVTERLSWEIQAPLTV-RPSDFK- 313
75139                         --WSPGVDY-RGPTCASCHMSGSGT------VMTSHDVTERLAWEIQAPLTV-RPSEFK- 320
823138                        --WTAGVDY-RGPTCAACHISGSGS------VKTSHDVTERLAWEIQAPLTV-RPSEFA- 252
669580                        --WSPGVDF-RGPTCASCHMSGAGS------VQTTHDVTERLSWETQAPLTV-RPSEFK- 291
141311                        --WTPGVDF-RGPTCASCHMSGAGD------VTTSHDVTERLAWELQAPLTI-RPSEFK- 314
773104                        --WTPGIDF-RGPTCASCHMSGAGT------VLTTHDVTERLAWEIQAPLTV-RPSEFK- 186
778733_Desulfobacteraceae     --WSAGTDY-RGPTCATCHISGAGT------TLTTHDVTERLSWEIQAPLTI-RPSEFK- 256
```

992	90565	--WTAGVDY-RGPTCATCHMSGVGD------VKTSHDVTERLAWEIQAPLTV-RPSEFK-	312
993	63845	--WTAGVDF-RGPTCASCHMSGTGP------VLTSHDVTERLAWEIQAPLTI-RPSEFK-	169
994	194073	--WTPGVDF-RGPTCASCHMSGSGT------VLTSHDVTERLAWEIQAPLTI-RPSEFK-	318
995	523303	--WQAGRDF-RAPTCAACHMSAAGG------IPRSHDVTERLAWETQAPLTV-RPSDFE-	487
996	693944	--WTAGRDY-RAPTCAACHMSEAGD------LAASHDVTNRLSWELQAPLTI-RPSEFT-	270
997	234068	--WRAGRDY-RAPTCASCHMSAAPG------VARSHDVTERLSWELQAPLTI-RPADFA-	593
998	44033_Deltaproteobacteria	--WTAGRDY-RAPTCAACHMSEAGE------VEKSHDVTGRLAWELQAPLTI-RPEEFP-	668
999	591311	--WTAGVDY-RAPTCASCHMSEAPG------VEKSHDVTRRLAWELQAPLTI-RPSEFA-	206
1000	WP_015335051.1	--WQPG-DY-DAPTCAVCHMSGIGE------LSTSHNVNERLKWDLMHKKSVIRSGER--	316
1001	WP_022853624.1	--WEPG-DY-SAPTCATCHMSGIGE------LATTHNVTERLKWDLVHKKSVIRSGER--	315
1002	WP_007473950.1	--QIPGKTL-RAATCFTCHMSGING------LKATHNVSLRLKWNLWAPASFLRTGGNET	342
1003	WP_015902282.1	--QIPGKTV-RAGTCFTCHQAAIGG------LKSTHNVSLRLKWNLWAPGSFLRTGGYET	342
1004	WP_002849252.1	--WDVP-DY-RAPTCATCHMSGIGD------LNTTHNVSVRLKWNLWAPHSNLRTGGYDT	318
1005	WP_011992073.1	--WDVP-DF-RAPTCATCHMSGVGE------TTTTHNVSQRLKWNLWAPRSELRTKGYEQ	312
1006	307368_Wenzhouxiangella	--WEAGKDF-RGPVCATCHMAGVGD------LKTTHNISERLHWVLWSKRSALRDSDD--	310
1007	553040_Thialocapsa	--WEPG-DY-RAPTCATCHMSGVGE------LSVTHNVSERLYWNLWAKRSDVRGSE---	329
1008	WP_051227586.1	--WEPG-DY-RAPTCATCHMSGIGE------LSTTHNINERLYWNLWAKESKVRNSTD--	316
1009	160849	--WEPG-DY-RAPTCATCHMSGIGE------LKTTHNITERLYWNLWAKVSKVRHSDD--	315
1010	595128_Anaerolineaceae	--GTQDVTDFPAATCAICHMSGFGA------TGTTHDVGDRLTWFLAAPVSERR------	297
1011	94026	--GTLTVNDFPAATCAICHMSGFGA------TGTTHDVGDRLTWYLFAPISERR------	315
1012	296856_Chloroflexi	--GTLTVQDFPAATCAICHMSGFGA------SGTTHDVGDRLTWYLFAPISERR------	306
1013	92136	--GTLTVEDFPAATCAICHMSAFGQ------STTTHDVGDRLTWYLFSPISERR------	167
1014	167239	--WQVGVDYFRAPTCATCHMSATPK------QPITHNVGDRISWTLRPIISTTK------	278

```
414796                        --WVVGVDYYAAPTCATCHMSATRN------QPITHEVGDRISWTLRPPVSSTK------ 274
547394                        --WVVGQDYWVAPTCATCHMSATSN------QEITHNVGDRISWTLRPPVSHTK------ 275
218326                        --WVVGQDYHAAPTCVSCHMSATDE------QAVHHDLGRRIAWTLRPPVSTYK------ 274
328                           --WVVGEDYNVAPTCATCHMSATRT------QDFTHDVGERIAWTNRPVISKHK------ 277
87192_Ca._KSB1                --WIVGEDYFAAPTCASCHMSATSK------QRYNHDVGRRISWTCRPLFSTKT------ 272
289884_Ca._KSB1               --WVVGEDYYAAPTCATCHMSATETQ-----KKYSHDVGMRISWTCRPLFSTKT------ 273
66390                         --WVVGVDYSVAPTCATCHMSATQS------QELTHDVGQRISWTLRPAISVMK------ 300
238994                        --WVVGIDYYVAPSCATCHMSATPS------SSLTHDVGRRISWTLRPKISVHK------ 276
398289__Ca._GN15              --WVVGIDYNAAPTCATCHMSATPD------MKANHDVGLRISWTLRPVFSTHK------ 280
605530                        --WVLSEDYSAAPTCATCHMSATPS------QDVSHDVGLRISWNNRPAISIRPELSDKK 208
722065_Spirochaetes           --WIVGVDYTAAPTCATCHVSATLN------QSRTHDIGLRISWNLRPAISIKT------ 286
82701_Desulfomonile           --WIVGVDYQDTATCVTCHMGATPN------QGSTHDVGTRISWTLRPAISKKL------ 286
468909_Gammaproteobacteria    --WVAGIDYTAAPTCATCHMSAAPH------EASTHNVGERISWTLRPPISTKINLVKLE 314
104369                        --WLAGVDYWAAPTCATCHMSAAPG------IAPTHNVGERISWTLRPPISTKINLVKLE 295
122072                        --WVAGVDYSAAPTCATCHMSAAPN------EGKTHNVGERISWTLRPPISKRINLVKLE 297
194837                        --WEPGRHYIEAPSCATCHMGGAGS------LRPTHDVGMRNAWNLHAPISEQQYLVVLE 302
272093_Gammaproteobacteria    --WVAGQDYTAAPTCVTCHMGAAGK------VPATHDVGMRNAWALNTPVSQRQYLVVLD 303
                                            *  **
```

```
                                                                                        Hemo8
sp|Q1PW30.2|HDH_KUEST           ASIVYTSMGM---------SMADR------------------GAPLWKE-KRDRWVSICD 343
91837                           KQWASDRVT---ILKALGVLNPETGEPTARLDVVKAADLARLTQEAWET-EREKMIDRCK 301
sp|Q50925.2|HAO_NITEU           --------------------------------------------SDWSEARLDSWVLTCT 385
SCZ85870.1                      --------------------------------------------SDWSEARLDSWVVTCT 387
28153                           TSIVKNAEGL--QLPTSLDGKLAK--------------DYLISEGEMAK-RKESMQKVCL 394
316357                          TTILRNRAGL--PLPVELDGRPAP--------------AGLIGEEEQAR-RRTVMKGVCR 384
91836_Desulfobacterales         TTGIRNAAGL--PLPTELTGEPVQ--------------AFLIDEKEQKK-RQQTMQAVCL 389
778484                          TSIIRNNSGL--PLPTGLTGEPAS--------------KYLIGKAEQDR-REKTMKSVCL 384
237805_Desulfosarcina           TSIIRNKSGL--PLPTNLDGSFAA--------------DHLISKDEVAS-RRAAMQRTCL 386
15066                           TTKIRNSQGL--PLPTDFQNRPAS--------------EFLISKEEQAA-RRTTMQAICT 388
628688                          LSNVNNSMGL--PIATELDATPVA--------------EFVISKKEQKQ-RNQNMKNICL 361
90566                           -------------------PFPFK--------------------GSWKR-ERKKMKSVCR 600
648186_Desulfovermiculus        -------------------PWPAE--------------------TNWKE-ERAKMQAVCT 334
346596                          -------------------PFPAD--------------------TNWKV-EREKMKEICS 167
247013                          -------------------AFPAN--------------------TDWKT-ERDKMKNVCS 328
474106                          -------------------PFPSG--------------------TDWRE-ERAK------ 327
75139                           -------------------PFPAK--------------------TNHET-ERKKMKEICR 340
823138                          -------------------AFPAQ--------------------TSWRE-EREKMSAVCQ 272
669580                          -------------------AFPAK--------------------TNWQV-EREKMQEICR 311
141311                          -------------------PFPAQ--------------------TNWRT-ERKKMKTICK 334
773104                          -------------------PFPAK--------------------TNWEV-ERKKMSEICM 206
778733_Desulfobacteraceae       -------------------PFPAK--------------------TNWRT-ERDKMKAVCT 276
```

90565	-------------------PLPAK--------------------TNWET-ERNKMKLVCK	332
63845	-------------------PLPAK--------------------TNWRT-EREKMKSVCK	189
194073	-------------------PFPAK--------------------TNWQT-EREKMKEVCM	338
523303	-------------------PFPAE--------------------TDWKA-ERRKMRAVCL	507
693944	-------------------PFPSG--------------------TGWEE-ERAKMKTVCL	290
234068	-------------------PFPAK--------------------TSWAE-ERRKMQQVCL	613
44033_Deltaproteobacteria	-------------------PSPAD--------------------IDWRT-ARSNMRTVCL	688
591311	-------------------PFPAS--------------------TDWRE-ERERMKAVCY	226
WP_015335051.1	--GDGEK-GDKLMRKVCV	331
WP_022853624.1	--GDGER-GRILMRKVCV	330
WP_007473950.1	AGWAFWNGGGKVTENTVTRGNPKAGN-----------------PNGPEA-ARAQMKQVCM	384
WP_015902282.1	AGWAFWKGGGKINPDTVIRGNAKAGN-----------------PQGPEA-ARAEMKKVCM	384
WP_002849252.1	AAETYAKEGK------ISIGTPLAGN-----------------INGPEA-ARTEMKQVCK	354
WP_011992073.1	AAYDYWKTGK------LNTGTPLAGN-----------------PQGPEA-ARAEMKLVCK	348
307368_Wenzhouxiangella	------------------PMSPVLG--------------------DADA-GRAKMKQVCA	331
553040_Thialocapsa	--	329
WP_051227586.1	------------------VMSPLLG--------------------NGPE-GRKKMEQVCS	337
160849	------------------VMSPWYG--------------------DGPA-GREKMEQVCM	336
595128_Anaerolineaceae	--PAWQD-NKVRMQAVCS	312
94026	--PGWED-NQVRMQNVCL	330
296856_Chloroflexi	--PGWED-NQVRMQNVCV	321
92136	--PGWQG-NQRRMQNVCT	182
167239	--ENWEV-KRESMQEVCR	293

```
414796                          --------------------------------------------DNWEI-KRQNMQDVCR 289
547394                          --------------------------------------------DNWKM-KRGNMQDVCR 290
218326                          --------------------------------------------ENHTE-RRARMKEVCS 289
328                             --------------------------------------------ENHVQ-KRTAMQDVCL 292
87192_Ca._KSB1                  --------------------------------------------ENWVE-KRNNMKDVCR 287
289884_Ca._KSB1                 --------------------------------------------ENWVA-KRNNMKSVCR 288
66390                           --------------------------------------------EDWER-KRANMKDVCT 315
238994                          --------------------------------------------ENWQD-RRRNMQQVCI 291
398289__Ca._GN15                --------------------------------------------DNWRE-KRSRMKKVCV 295
605530                          MG----L-------PG--------------------------ANINWQT-RRSNMKDVCL 230
722065_Spirochaetes             --------------------------------------------EESED-KRRAMQEVCM 301
82701_Desulfomonile             --------------------------------------------SKWKD-RREKMQDVCR 301
468909_Gammaproteobacteria      DGNEFDV-------PE-GEEIPAVGDAA--------RGSTVVEVLTWED-RRKKMEDVCY 357
104369                          NGNEFDI-------PGEDAELPAVGDEA--------KGSKVAEVLSWEE-RRDNMKTVCR 339
122072                          NGDEFDV-------PE-GQEIPKVGDEA--------KGSKVVEVLTWEQ-RRDKMKTVCR 340
194837                          SGDKYNL-------PVSR-KPPRKGDPVTKPDG---GKGLVKAVATPER-RRQAMIQVCR 350
272093_Gammaproteobacteria      DGSKLEL-------PADT-KPPKRGSELTKADG---STAKVKVVATPER-RRLVMSTVCL 351
```

```
sp|Q1PW30.2|HDH_KUEST       DCHSPRFARENLQAMDESVKDASL-KYRETFKVAEDLLIDGVLDPM-------PKDLCP- 394
91837                       VCHAENYARKQLQMGDTMMQKADR-LMAEAIEIVAGLYADGLI----EKPEAYTFAY-P- 354
sp|Q50925.2|HAO_NITEU       QCHSERFARSYLDLMDKGTLEGLA-KYQEANAIVHKMYEDGTLTGQKTNRPNPPEPEKPG 444
SCZ85870.1                  QCHSERFARSYLELMDKGTLEGLA-KYQEAHELVEKLYKTGNLTGQKTNRPAPPEPEKPG 446
28153                       SCHSSNWVNGHWQRFENTIQVTND-MTKTATELLIKAWEQGTAD----KSNFFDEGI--- 446
316357                      SCHGAEWVDNQFKVLDRTHETTNA-STLAATQIMSRIWSRGYADGLDKQDSLFNEAI--- 440
91836_Desulfobacterales     SCHSAPWVKGQYALFESAIDTTNK-MTLAATQILLSAWEQGAAKGMAQGDSIFNEAI--- 445
778484                      SCHAGGWVDSHFSRLE-------------------------------------------- 400
237805_Desulfosarcina       ACHDTSWVRGYFARLDNTITTSNQ-SVKVATQLMQSVWDKGYAQGLAGGGSIFDEYI--- 442
15066                       QCHGGSWTANHFERLDNTIATTNQ-ATLTGTTMMLEAWDLGLASGLAQGQSLFDEGI--- 444
628688                      NCHNSEWVDNHFIRLDNTIQKTNL-ATVQGTQILAEVWQQELAQGLPQKQNIFDEEI--- 417
90566                       PCHSSTWVDSHFRRYDRVIEEYNENYFRPANKKMEELYEKGLL----DNRRFFDESL--- 653
648186_Desulfovermiculus    QCHGMTWVEDHYVKFDKVVQEYNEVYYKPAKAMLDTLYAEGLL----DDEKFFDEKL--- 387
346596                      QCHSQRWTDAHYQDMDKAIEEYNEVYFKPAKATLDKLYAKGLL----DKDSFFDEKL--- 220
247013                      QCHGKAWTDNHYATLDRVIEEYNEVYFKPAKKMLDNLYEKELL----NKEKFFDEHL--- 381
474106                      ------------------------------------------------------------ 327
75139                       QCHGDTWIDAHFNKMDKVVEEYNEVYFKPAKKMLDDLYDKGLM----EPKPFFDEEL--- 393
823138                      QCHGKTWTDAHFVKFDRVVEEYNEVYFKPAKALLDELYEAGAM----EKKPFFDERL--- 325
669580                      QCHGKNWVTDHYEKLDQVIEEYNSVYYKPAKNMLDKLYEKGRL----DQSRFFDEKL--- 364
141311                      QCHGKTWVDAHFTKQDQVVAEYNTVYFKPAKKMLDDLYAKGLM----DNTRFFDEQM--- 387
773104                      QCHGKTWVDDHFIKLDKVVSEYNEVYFKPAKQMLDSLYEKGHL----DNTRFFDERL--- 259
778733_Desulfobacteraceae   QCHGKTWVDDHYVKLDKVVEEYNEVYFKPAKKMLDDLYDKGLL----DKTKFFDERL--- 329
90565                       QCHGKTWVDDHFKKYDKVVEEYDNVYFKPAKKKLDELYSKNLM----DKTKFFDEQL--- 385
```

1124	63845	QCHGKTWVDAHFTTLDRVVEEYNEVYFKPAKAKLDELYAKDLM----DKSRFFDERL---	242
1125	194073	QCHGKTWVDGHFVKQDRVMTEYNDVYFKPAKKMLDDLYAKDLL----DKSRFFDEVM---	391
1126	523303	QCHSNGWADEHFRTYDRVVDHYNQIYYLPARKVMNDLYDEGLL----SRKRTFDEAL---	560
1127	693944	QCHSEDWTEGHFRNMDTVVETYNELYYKPIKEKMDRLYEEGKL----SRNSYFDEEL---	343
1128	234068	QCHSVAWTSGHFDNLDAVIHNYNEIYYEPVHQVLTDLYAAGLL----AEDSYFDEPL---	666
1129	44033_Deltaproteobacteria	QCHSEGWTDGHFENMDTVINHYNEEYYKPIKKMMDELYAQGLL----TEDSYFDEEL---	741
1130	591311	QCHSGEWVEGHFTNTDNVIERYNSLYYEPVKAVMDGLYAEGAL----SEDYYFDEEL---	279
1131	WP_015335051.1	NCHGQTHTDVQRQLLDDAVALYNT-YWDGAVKMKKELADKGLL----LTDDPWNDGF---	383
1132	WP_022853624.1	NCHSKVFVDAHFNKLDNSVALYNF-YWDKVTAMVNDLKQKGLL-----KKDKWSDPV---	381
1133	WP_007473950.1	ACHAATFTNNFFQRADAHVKVYNQ-YKAFATKMLKELKAKGLM-----KADLWSDPF---	435
1134	WP_015902282.1	VCHEATFTNNYFQRIDAAVTVYNQ-YKSFATKMLKDLKAKGLM-----KSDVWSDPF---	435
1135	WP_002849252.1	SCHSSKATDSFFVSADNHVELYNT-YHTEAKKMLDDLKAKGLL-----KKDEWSDEF---	405
1136	WP_011992073.1	TCHTITHTDNFFAMGDKQVQLYNV-YYDEAKKMLDDLKAKNLL-----LEDAWEDEF---	399
1137	307368_Wenzhouxiangella	ECHSSAHTDNFFQQGDKAVKLYNQAYYDPAVKMRDELAEKGLL-----KENPWIDEF---	383
1138	553040_Thialocapsa	--	329
1139	WP_051227586.1	SCHSSTHTKGFFKQGDKAVKLYNVEYYAPAKQMLDELKEKGLL-----KDNPWTDEF---	389
1140	160849	SCHSETHTKGFFKQGDKAVRLYNEAYWKPAKAMKDELAEKGLL-----KENPWADEF---	388
1141	595128_Anaerolineaceae	ECHNENFINTFYHNADLVVEQVNL-WVEESDEIVQPLKDNDLM----TD-EPFDEPI---	363
1142	94026	ECHNSEFIDSFYGDADRLTEAINA-WVEESNQIIAPLKEQGLL----TA-EPFDEPI---	381
1143	296856_Chloroflexi	ACHNQEFIDTFYTDADKLTEAIND-WVRESDEIIAPLKEEGLL----TA-APFDEPI---	372
1144	92136	ACHNEEFITTFYTDADKLTEAINA-WVEESDRLVAPLKEQGLL----TS-EPFDEPI---	233
1145	167239	NCHGQGFVDGHYAMMDGVINLYNDKFARPAKRLYDLVAELGLR----KNVASFSNKY---	346
1146	414796	ACHGQGFVDGHYAQLDGVVNLYNEKFARPAGELHRIAKDKGLG----DGTANFSNKY---	342

```
547394                        NCHGQRFVDGHYAQFDGVVNMYNEKFAKPAGRLHRIAKDKGLG----KNVANFSNKY--- 343
218326                        TCHGTTFVDGHFYQFDAVVRLYNDKFARPATRIMNRVWEKDLL----DYPAEFANEL--- 342
328                           ACHGDTFADGHFYQYDASVKLYNEKFAKPAQQIMNKIKEKDLL----ETPAAFSNDV--- 345
87192_Ca._KSB1                TCHGDVFVAGHYYQYDATIRLYNEKFAKPANRIMTLVRQQNLL----EKPAHFANQI--- 340
289884_Ca._KSB1               TCHGDVFVNGHYYQYDATVHLYNEKFAGPASRIIALIRQKDLR----EYPAEFANRI--- 341
66390                         NCHGAHWVDGHYWQFDGVVQLYNEKFAEPAGRIMEIVRRKDLM----QHPAEFANEI--- 368
238994                        NCHGQTFVDGHYYLYDAVVRLYNDKFARPAGELMSIIESKQLK----KNPADFANKV--- 344
398289__Ca._GN15              NCHGKTFADGHYYQFDAVVRIYNEKFAQPSTDIMN------------------------- 330
605530                        NCHNTNFINGFYKQYDALIELYHNKFARPGLELYNL--AQPLL----SE-PKFSNPI--- 280
722065_Spirochaetes           NCHNPNYVENFYQ----------------------------------------------- 314
82701_Desulfomonile           QCHGQFMIEGFYTQFDNLVNLYNEKFAIPATEVRKRLMANGKL----TE-ANFDDKL--- 353
468909_Gammaproteobacteria    ACHEQSVIDGHYKQFDDVVVLYNEKFAKPIAAIMGKLKDGGYI----TS----------- 402
104369                        ACHSQGMVEGFYRQFDNVVVLYNEKFAKPIAAIMNELKKEGYI----TQ-SPFDEPI--- 391
122072                        ACHTSSVVEGHYKQFDDVVHLYNEKFAKPIAAIMKELKENGYI----SK-EPFDDKI--- 392
194837                        QCHGERTAQRYMEEFDQAVELYNSKFAQPARDMMQALYATKKL----TP-APFDEPL--- 402
272093_Gammaproteobacteria    ECHSKTMTEGFMRQFDDVVELYNTKFGEPAAAIMAGLYDAGLL----TP-LPFDEPL--- 403
```

```
sp|Q1PW30.2|HDH_KUEST         DWSGQHI-WSLKIGAYHDGEAYGGTTGESGEFRMSNCTDVERLCFESVGYFQTYIYKGMA 453
91837                         DFLYFM--RTGG------GNFDQA-------------SYIDQVLFQMYMKHRMRTYQAFF 393
sp|Q50925.2|HAO_NITEU         FGIFTQLFWSKG------NNP----------------ASLELKVLEMAENNLAKMHVGLA 482
SCZ85870.1                    FHIFTQLFWSKG------NNP----------------ASIELKVLEMAENDLAKMHVGLA 484
28153                         EKQWVEQ-WLFY------ANS----------------T---------------R--FASA 466
316357                        EKTWVEH-WLFY------ANS----------------T---------------R--YAAA 460
91836_Desulfobacterales       EKAWVEQ-WLFY------ANT----------------T---------------R--YAAA 465
778484                        ------------------------------------------------------------ 400
237805_Desulfosarcina         ERRWSDT-WLLY------ANN----------------I---------------R--FVSA 462
15066                         EKLWSRT-WLID------ANT----------------V---------------R--FASA 464
628688                        ERMWTSI-WLFY------ANS----------------T---------------R--FASA 437
90566                         EYEYFEL-WRHE------GRR----------------A---------------R--MGAA 673
648186_Desulfovermiculus      EVEFYEL-WHHE------GRR----------------A---------------R--MGAA 407
346596                        EVEFYEL-WHHE------GRR----------------A---------------R--MGAS 240
247013                        EVEFYEL-WHHE------GRR----------------A---------------R--MGAA 401
474106                        ------------------------------------------------------------ 327
75139                         EVEYYEL-WHHE------GRR----------------A---------------R--MGAA 413
823138                        EVEYYEL-WHHE------GRR----------------A---------------R--MGAA 345
669580                        ENEFYEL-WHHE------GRR----------------A---------------R--MGAA 384
141311                        EFEFYEL-WHHE------GRR----------------A---------------R--MGAA 407
773104                        EVEFYEL-WHHE------GRR----------------A---------------R--MGAA 279
778733_Desulfobacteraceae     EVEYYEL-WHHE------GRR----------------A---------------R--MGAA 349
90565                         EVEYYEL-WHHE------GRR----------------A---------------R--MGAA 405
```

1189 63845 EVEFYEL-WHHE------GRR----------------A---------------R--MGAA 262
1190 194073 EVEYYEL-WHHE------GRR----------------A---------------R--MGAA 411
1191 523303 EWEFYEL-WHHE------GRR----------------A---------------R--MGTA 580
1192 693944 EWEYYEL-WHHE------GRR----------------A---------------R--MGAA 363
1193 234068 EWEFYEF-WHHE------GRR----------------A---------------R--MGAA 686
1194 44033_Deltaproteobacteria EWEYYEL-WHHE------GRR----------------A---------------R--MGAA 761
1195 591311 EWEFYEL-WHHE------GRR----------------A---------------R--MGAA 299
1196 WP_015335051.1 QELMYYL-WHHC------GRR----------------A---------------R--HGTA 403
1197 WP_022853624.1 QELTYYF-WHHV------GRR----------------A---------------R--HGAA 401
1198 WP_007473950.1 FKLYYYL-WHHE------GRR----------------M---------------R--QAAV 455
1199 WP_015902282.1 FKLYYYL-WHHE------GRR----------------F---------------R--HGAA 455
1200 WP_002849252.1 QITYYYL-WHHQ------GRR----------------M---------------R--MGAV 425
1201 WP_011992073.1 QDVFYHL-WHHE------GRR----------------M---------------R--QGAL 419
1202 307368_Wenzhouxiangella QITFYHL-WHHQ------GRR----------------A---------------R--MGAM 403
1203 553040_Thialocapsa -- 329
1204 WP_051227586.1 QITFYHL-WHHE------GRR----------------A---------------R--HGAM 409
1205 160849 QKTFYHL-WHHQ------GRR----------------A---------------R--QGAL 408
1206 595128_Anaerolineaceae DYVYFNL-WHHW------GRT----------------A---------------K--FGTW 383
1207 94026 DFTYFNL-WHHW------GRT----------------A---------------K--FGAW 401
1208 296856_Chloroflexi DFTYFNL-WHHW------GRT----------------A---------------K--FGAW 392
1209 92136 DFTYFNL-WHHW------GRT----------------A---------------K--FGAW 253
1210 167239 EWTFWEL-WHHE------GRR----------------A---------------R--HGAA 366
1211 414796 EWIYWEI-WHHE------GRR----------------A---------------R--HGAA 362

```
547394                          EWIYWEI-WHHE------GRR----------------A---------------R--HGAA 363
218326                          EWTYWEL-WHHE------GRR----------------A---------------R--HGAS 362
328                             EWAFWEL-WHHE------GRR----------------A---------------R--HGAS 365
87192_Ca._KSB1                  EWTFWEL-WHHE------GRR----------------A---------------R--HGSA 360
289884_Ca._KSB1                 DWIFWEL-WHHE------GRR----------------A---------------R--HGAA 361
66390                           EWIYWEL-WHHE------GRR----------------A---------------R--HGAS 388
238994                          DWIYWEL-WHHE------GRR----------------A---------------R--HGAS 364
398289__Ca._GN15                ------------------------------------------------------------ 330
605530                          DFTWFEI-WHHE------GRR----------------A---------------R--HGAS 300
722065_Spirochaetes             ------------------------------------------------------------ 314
82701_Desulfomonile             DWIYWEL-WHHE------GRR----------------A---------------R--HGAS 373
468909_Gammaproteobacteria      ------------------------------------------------------------ 402
104369                          EWTWWEI-WHHE------GRR----------------A---------------R--HGAS 411
122072                          EWTWWEI-WHHE------GRR----------------A---------------R--HGAS 412
194837                          EFTYWKL-WHDA------GIR----------------A---------------R--QGAA 422
272093_Gammaproteobacteria      EYTYWEL-WHDE------GAR----------------A---------------R--HGAS 423
```

RESIDUAL TYROSINE

```
sp|Q1PW30.2|HDH_KUEST         HGS-WNDATYSDGSFGMDRWLV-----NVKQNASRARRLAA------------LEK----  491
91837                         HVN-PDYAYW-YGWAMMTKDLA-----EIKKLAQTMRATTMKKE----------------  430
sp|Q50925.2|HAO_NITEU         HVN-PGGWTYTEGWGPMNRAYV-----EIQDEYTKMQELSA------------LQARVNK  524
SCZ85870.1                    HTN-PGGWTYTEGWGPINRAYV-----EIQDEHTKMQAMAA------------LQERVNK  526
28153                         MMG-ADYGVFANGRWYLSKNIQ-----EMIDKIKSLPNKGTEAKS---------------  505
316357                        MMG-GDYGVFANGRWFMARNIQ-----YMMDWLKNAERSSARAAD---------------  499
91836_Desulfobacterales       MIG-ADYGAFANGRWQMSKNIQ-----EMMDRLSFMLKE---------------------  498
778484                        ------------------------------------------------------------  400
237805_Desulfosarcina         MAGGGDYGVFEDGRYHLSKAIM-----DMHDWQQTRDLIMKKP-----------------  500
15066                         MAGGGDYGVFANGRYELATHLA-----EMRDWIELRKGLEAQPAS---------------  504
628688                        MAGGGDYGVFANGRFQMTDQLI-----KMQKWLEREKKSSEIK-----------------  475
90566                         MMA-PDYSWW-HGFYECKKRYA-----GIMEEADKLLEGNKKALR--------RKD----  714
648186_Desulfovermiculus      MMA-PDYTWW-HGFYECKNRFN-----AFMAEAEHLLATGEPAHV--------Y------  446
346596                        MMA-PDYAWW-HGFYECKHRFN-----EFMTEAGHLLETGQAAHV--------YED----  281
247013                        MMA-PDYAWW-HGFYECKKRFN-----SYMEAANHLLKTGEAAYR--------YPN----  442
474106                        ------------------------------------------------------------  327
75139                         MMA-PDYAWW-HGFYECKKRYN-----TFMEEARELIHENKKAYK--------AED----  454
823138                        MMA-PDYAWW-HGFYECKKRFN-----AFMEEGRELLH----------------------  376
669580                        MMA-PDYAWW-HGFYECKKRYN-----RFMSEAKELIQENRKAHV--------YED----  425
141311                        MMA-PDYAWW-HGFYECKQRYT-----QFMAEGRHMLKTGQKAEK--------AKD----  448
773104                        MMA-PDYAWW-HGFYECKKRFN-----NYMEEARELIEHDKKAYV--------AED----  320
778733_Desulfobacteraceae     MMA-PDYAWW-HGFYECKKRYS-----NFMEEARHLIDNNQKAYV--------AED----  390
```

```
1254  90565                        MMA-PDYAWW-HGFYECKKRFV-----NFMHEANLLIENNEKAYK--------AKD----  446
1255  63845                        MMA-PDYAWW-HGFYECKKRYV-----NYMEEANELIHENRKAYK--------AEN----  303
1256  194073                       MMA-PDYAWW-HGFYECKKRFN-----TFMEEGRHMIENNRKAYK--------AED----  452
1257  523303                       MMA-PDYAWW-HGFYELKHRFG-----LLMVEAEKLRAGE-PATT--------FHP----  620
1258  693944                       MMA-PDYAWW-HGFYELKHRFA-----VISEMIETQTEDD-RGEW--------IQL----  403
1259  234068                       MMA-PDYAWW-HGFYEVKHRYV-----TFMAEAEELRELG-QPRE--------YEH----  726
1260  44033_Deltaproteobacteria    MMA-PDYAWW-HGFYECKHRFI-----TLIDKIEAQTEED-WGEW--------IEV----  801
1261  591311                       MMA-PDYAWW-HGFYELKHRFV-----HFMELAREHTPEA-KAHW--------LED----  339
1262  WP_015335051.1               MNG-PDYSHW-HGFFQVFQVYK-----DMQKIHEYRLKNG----K--------IEE----  440
1263  WP_022853624.1               MAG-PDYAHW-HGFFQLFQVYK-----DIQALYNYRMKHG----K--------IEE----  438
1264  WP_007473950.1               MGS-PDYAHW-HGVFQVMQDIR-----EMKDIYDYRMKML----K--------KYK----  492
1265  WP_015902282.1               MGS-PDYAHW-HGVFQVMQDIR-----EMKDIYDYRMKML----K--------KYG----  492
1266  WP_002849252.1               MGA-PDYAHW-HGVFEVQQDIK-----KLRTIYDARIKSG----K--------IE-----  461
1267  WP_011992073.1               MGG-PDYSHW-HGVFEVKNDIR-----KLRKIYKERIESG----K--------AH-----  455
1268  307368_Wenzhouxiangella      HGA-ADYAHW-HGFFELMQDIY-----ELKEIYNRRIETG----E--------IE-----  439
1269  553040_Thialocapsa           ------------------------------------------------------------  329
1270  WP_051227586.1               MGA-PDYAHW-HGFFELQQDLY-----KLKAIHKKRLETG----K--------IED----  446
1271  160849                       MGG-PDYAHW-HGFFELQQDIY-----KLREIYERRLETG----K--------IGG----  445
1272  595128_Anaerolineaceae       MQG-PDYVQW-HGAYEVLHE-----RAELIKLTNEKLEAAGLETIEPGRPP-LIEELPID  435
1273  94026                        MQG-ADYVQW-HGAYEILHE-----LAELREMTETKLQEAETGE----------------  438
1274  296856_Chloroflexi           MQG-ADYVQW-HGAYEVLHE-----LAELREMVEAKQKEAE-------------------  426
1275  92136                        MQG-PDYVQW-HGAYEVLHE-----LAELRHMVDEKLAEANGGEQ---------------  291
1276  167239                       MMG-PDYTWW-HGIYEVGQHFYFKYIPQLRDLDNAQLDA-------------AIDEIL-A  410
```

```
414796                          MMG-PDYTWW-HGIYEVAQHFYFKYIPELRKFHDPDLDA-------------AIDRIL-A 406
547394                          MMG-PDYTWW-HGIYEVAQHFYFKYIPELRKLDDADLNA-------------AIEDVL-A 407
218326                          MMG-PDYTWW-HGMYEVVEHFYLKFVPQVRDLDDPELNA-------------ALDSLLQT 407
328                             MMG-PDYTWW-HGFYDVLHNFYFDFLPKAREYGDSEVNA-------------MIDDLLQN 410
87192_Ca._KSB1                  MMG-PDYTWW-HGFYEVAKHFYLKFIPEAQSFRNETVNG-------------VIDSLFTH 405
289884_Ca._KSB1                 MMG-PDYTWW-HGFYEIAKHFYLDFLPAARSFQDAEINA-------------VIDSLLTN 406
66390                           MMG-PDYTWW-HGIYEVGKHFYIEFVPTARHFGDPEVNA-------------ALDSIL-A 432
238994                          MMG-PDYTWW-HGMYEVAKHFYIDFVPELRNFNDPDINA-------------YLDSLITE 409
398289__Ca._GN15                ------------------------------------------------------------ 330
605530                          MMG-PDYTHW-HGTYEVAKHFYSDYIPELRELIDHGLDSDSSDKVQAAEKLQSRLEEILN 358
722065_Spirochaetes             ------------------------------------------------------------ 314
82701_Desulfomonile             MMG-PDYAWW-HGMYEVAKHFYNKFLPEVEHLMGGKEA----A-----APL--LDEVVFK 420
468909_Gammaproteobacteria      ------------------------------------------------------------ 402
104369                          MSG-PDYTWW-KGIYEVAKHTYFQFIPQLKEVVAAKDGNEDFA-----DRM--MEEYFRP 462
122072                          MSG-PDYTWW-HGIYDVAKHTYFKFIPELKEVARAKDGNEQFA-----KAM--LDKYFRP 463
194837                          MSS-PQYAWW-WGMNQVAELFYGHFLPQAKNLAGE---A-------------FIRQHLKD 464
272093_Gammaproteobacteria      MMS-PNHAWW-EGMYVVGRNFYSRFLPEARAVAGEQAAA-------------LVDAHIDG 468
```

```
sp|Q1PW30.2|HDH_KUEST          KVGISWQPEQFWKTGE--------------WLDQLTGPY---IVKNHPGKTIFDLCPDPG 534
91837                          ------------------------------------------------------------ 430
sp|Q50925.2|HAO_NITEU          LEGKQTSLLDLKGTGEKISL-----GGLG---GGMLLAGALALIGWRKRKQTR--A---- 570
SCZ85870.1                     LDGKKTSLLDITTPEEKISL-----GGLG---GGLLLAGTLALIGWRSRKRKQ--A---- 572
28153                          -QGK-------------------------------------------------------- 508
316357                         ------------------------------------------------------------ 499
91836_Desulfobacterales        ------------------------------------------------------------ 498
778484                         ------------------------------------------------------------ 400
237805_Desulfosarcina          ------------------------------------------------------------ 500
15066                          -D---------------------------------------------------------- 505
628688                         ------------------------------------------------------------ 475
90566                          FPK---------------------------AASRNQKPE--IQSPLKTNDRR-------- 737
648186_Desulfovermiculus       ------------------------------------------------------------ 446
346596                         FPN---------------------------DTGDTTKPK--EIFGTKE------------ 300
247013                         FPN---------------------------ATGNTERPP--EIFDR-------------- 459
474106                         ------------------------------------------------------------ 327
75139                          FPN---------------------------ATGDTTKPP--EIFGKSE------------ 473
823138                         ------------------------------------------------------------ 376
669580                         YP---------------------------------------------------------- 427
141311                         FPN---------------------------ATGSTQKPA--PIFGKPE------------ 467
773104                         FPN---------------------------ATGNTTVPA--EISKSRQ------------ 339
778733_Desulfobacteraceae      FPN---------------------------ATGDTT------------------------ 399
90565                          FPN---------------------------ATGSTKKPE--VIFGSQ------------- 464
```

63845	FPN---------------------------ATGDTTKPE--AVFGKTSQ-----------	323
194073	FPN---------------------------ATGSTVKPE--QIFGKTQ------------	471
523303	FPG---------------------------RFR---------------------------	626
693944	IPG---------------------------KYGETAGPD---------------------	415
234068	FPG---------------------------EYGDSLSSG--N------------------	739
44033_Deltaproteobacteria	FPG---------------------------RLKER-------------------------	809
591311	FPG---------------------------MLSEP-------------------------	347
WP_015335051.1	LSH---------------------------VMSTG--PL---------------------	450
WP_022853624.1	LSP---------------------------VMSSA--PY---------------------	448
WP_007473950.1	DPK---------------------------KVLENEPPM--PVVTHE-------------	510
WP_015902282.1	DPK---------------------------KALANEVPM--PVVTHE-------------	510
WP_002849252.1	--	461
WP_011992073.1	--	455
307368_Wenzhouxiangella	--	439
553040_Thialocapsa	--	329
WP_051227586.1	--	446
160849	--	445
595128_Anaerolineaceae	E---	436
94026	--	438
296856_Chloroflexi	--	426
92136	--	291
167239	EPFHAWMQ--RPTEEIKADI---ASGTLQEKYADLFAGP--QTSQ---------------	448
414796	DEFHQWLN--RPTGDIKADI---ESGRLQERYGEMFRAP--LASTK--------------	445

```
547394                          DPFHQWLN--RPTGDIKADI---QSGRMAEMFEEMYAGP--MEQ---------------- 444
218326                          DPLHSWVN--ESTESIRKRL---RSGEIQERYRQYFKAT--MESR--------------- 445
328                             DPMHQWVM--TDTETLKRRI---NEGTMQEVFVNMFDAY--IEAEAREDEEGTE------ 457
87192_Ca._KSB1                  DPMHQWIN--QETGKLRRDI---RNGSFEELYKQMFEQ---------------------- 438
289884_Ca._KSB1                 DPMHQWVN--QDTDELRRKI---RNGSFEAFYRQFFNR---------------------- 439
66390                           GPFHAWMQ--RPTEEIKRDI---RAGRLQEIFDDLYRPG--TTGGEG-----Y------- 473
238994                          DPMHGWVT--QPSSDLKEKI---RNGEIQEIYHQYYEDE--KDR---------------- 446
398289__Ca._GN15                ------------------------------------------------------------ 330
605530                          DQNHQWFL--NKMSPEEKERREERRKEFQKRYEN-------------------------- 390
722065_Spirochaetes             ------------------------------------------------------------ 314
82701_Desulfomonile             DERHRWYK--EGMSKKELQR---MQQFYERRYGGKE------IQ---------------- 453
468909_Gammaproteobacteria      ------------------------------------------------------------ 402
104369                          IEGHAWYF--EGMNTEMLKK---VRKGYEERYGKKA------FK---------------- 495
122072                          IDGHAWYF--EGMSKEAIEK---VRRGFEKRYGKGA------LK---------------- 496
194837                          LDAHQWLG--KPEQAHAI-------------LGETLHPP--REAPKEEKEEKE----KPE 503
272093_Gammaproteobacteria      ADGHAWLA--NQGEGNPI-------------LGWMPPPP--PEPAASADADAESTAPAPD 511
```

sp|Q1PW30.2|HDH_KUEST	WLDTHHAPAEEVEYIERKLKELGITAGSHSAHHH------ESGHDPAARSMKEH	582
91837	--	430
sp|Q50925.2|HAO_NITEU	--	570
SCZ85870.1	--	572
28153	--	508
316357	--	499
91836_Desulfobacterales	--	498
778484	--	400
237805_Desulfosarcina	--	500
15066	--	505
628688	--	475
**90566**	**--**	**737**
648186_Desulfovermiculus	--	446
346596	--	300
247013	--	459
474106	--	327
75139	--	473
823138	--	376
669580	--	427
141311	--	467
773104	--	339
778733_Desulfobacteraceae	--	399
90565	--	464

```
63845                          ------------------------------------------------------     323
194073                         ------------------------------------------------------     471
523303                         ------------------------------------------------------     626
693944                         ------------------------------------------------------     415
234068                         ------------------------------------------------------     739
44033_Deltaproteobacteria      ------------------------------------------------------     809
591311                         ------------------------------------------------------     347
WP_015335051.1                 ------------------------------------------------------     450
WP_022853624.1                 ------------------------------------------------------     448
WP_007473950.1                 ------------------------------------------------------     510
WP_015902282.1                 ------------------------------------------------------     510
WP_002849252.1                 ------------------------------------------------------     461
WP_011992073.1                 ------------------------------------------------------     455
307368_Wenzhouxiangella        ------------------------------------------------------     439
553040_Thialocapsa             ------------------------------------------------------     329
WP_051227586.1                 ------------------------------------------------------     446
160849                         ------------------------------------------------------     445
595128_Anaerolineaceae         ------------------------------------------------------     436
94026                          ------------------------------------------------------     438
296856_Chloroflexi             ------------------------------------------------------     426
92136                          ------------------------------------------------------     291
167239                         ------------------------------------------------------     448
414796                         ------------------------------------------------------     445
```

```
547394                          ------------------------------------------------------    444
218326                          ------------------------------------------------------    445
328                             ------------------------------------------------------    457
87192_Ca._KSB1                  ------------------------------------------------------    438
289884_Ca._KSB1                 ------------------------------------------------------    439
66390                           ------------------------------------------------------    473
238994                          ------------------------------------------------------    446
398289__Ca._GN15                ------------------------------------------------------    330
605530                          ------------------------------------------------------    390
722065_Spirochaetes             ------------------------------------------------------    314
82701_Desulfomonile             ------------------------------------------------------    453
468909_Gammaproteobacteria      ------------------------------------------------------    402
104369                          ------------------------------------------------------    495
122072                          ------------------------------------------------------    496
194837                          QKK----PAKS-----E-------------------------------------    511
272093_Gammaproteobacteria      SQE----PAAE-----R---APDADAGSAAVDAEDAAAVAHAGDTATEETTQ--    551
```

Text 1. Supplementary methods. Metagenomic data processing

Metagenomics processing with annotated code is documented at our Open-Science Foundation [11] site: https://osf.io/9kwn3/wiki. Conda (2020; www.anaconda.com (accessed on 10 March 2021)) was utilized for program installation and environment management. Quality was scanned with FastQC v0.11.9 [12] and reads trimmed/filtered with trimmomatic v0.39 [13]. A co-assembly of all 4 depths was performed with SPAdes v3.14.0 [14], the assembly was filtered and summarized with bit v1.8.16, and each individual samples' reads were mapped to the filtered co-assembly with bowtie2 v2.3.5.1 [15] and sorted and indexed with samtools v1.9 [16]. Co-assembly and read-mapping files were integrated into anvi'o v6.2 [17] for annotation with the KEGG database [18] and parsing and extraction of the gene-level coverage and detection data. Gene-level taxonomic classification was performed with CAT v5.1.2 [19].

Normalization and analyses were performed with R v3.6.3 [20] in Rstudio v1.1.456 (www.rstudio.com). To mitigate non-specific read-recruitment, gene-level coverage information was filtered based on detection (proportion of gene that recruited any reads to it), such that those with a detection less than 50% had their coverage set to 0. This had a net effect of removing less than 3% of the pre-filtered total coverage. Information on KEGG's Nitrogen Metabolism pathway (https://www.genome.jp/kegg-bin/show_pathway?map00910) was accessed with KEGGREST v1.26.0, defining the 65 KEGG Orthology (KO) terms we focused on. Filtering our gene table down to these KOs revealed 1,305 unique genes. Their coverages were normalized to within this Nitrogen Metabolism focus across the 4 samples by dividing each value by its sample's total coverage and multiplying by 1 million to generate values of Nitrogen-focused Coverage per Million (herein referred to as N-CPM). In order to compare the N-CPM of each nitrogen cycling gene to itself across depths examined, we also calculated a Fraction of Depth Integrated Coverage (herein referred to as FDIC). In the FDIC notation, the proportion of any given gene is normalized to a value of 1 across all depths.

Entrez Direct v13.9 (Kans 2021; www.ncbi.nlm.nih.gov/books/NBK179288/) was utilized to search and retrieve reference sequences from NCBI. For making phylogenetic trees, sequences were aligned with Muscle v3.8.1551 [21], and trees were generated with FastTree v2.1.10 [22]. Trees were visualized and edited through the Interactive Tree of Life web-interface [23]. Database of protein families and domains

(Pfam) [23] annotations on select targets (*hao*/*hcp*-annotated genes) were performed on the web-interface (http://pfam.xfam.org/search).

For generating Figure S6, HAO sequences were aligned with ClustalX v2.03 [24], and the alignment was visualized with JalView v2.11.1.4 [25].

References

1. Muyzer G, Waal EC, Uitterlinden AG. Profiling of complex microbial populations by denaturing gradient gel electrophoresis analysis of polymerase chain reaction-amplified genes coding for 16S rRNA. Appl Environ Microbiol, 1993;59:695-700.
2. Yu Z, García-González R, Schanbacher FL, Morrison M. Evaluations of different hypervariable regions of archaeal 16S rRNA genes in profiling of methanogens by Archaea-specific PCR and denaturing gradient gel electrophoresis. Appl Environ Microbiol. 2008;74(3):889-893.
3. Ben-Porath J, Zehr JP. Detection and characterization of cyanobacterial nifH genes. Appl Environ Microbiol. 1994; 60:880-887.
4. Tourna M, Freitag TE, Nicol GW, Prosser JI. Growth, activity and temperature responses of ammonia-oxidizing archaea and bacteria in soil microcosms. Environ Microbiol. 2008; 10:1357-64.
5. Rotthauwe JH, Witzel KP, Liesack W. The ammonia monooxygenase structural gene amoA as a functional marker: molecular fine-scale analysis of natural ammonia-oxidizing populations. Appl Environ Microbiol. 1997; 63:4704-4712.
6. Lücker S, Wagner M, Maixner F, et al. A Nitrospira metagenome illuminates the physiology and evolution of globally important nitrite-oxidizing bacteria. Proc Natl Acad Sci U S A. 2010; 107:13479-13484.
7. Henry S, Bru D, Stres B, Hallet S, Philippot L. Quantitative detection of the nosZ gene, encoding nitrous oxide reductase, and comparison of the abundances of 16S rRNA, narG, nirK, and nosZ genes in soils. Appl Environ Microbiol. 2006; 72:5181-5189.
8. Throbäck IN, Enwall K, Jarvis A, Hallin S. Reassessing PCR primers targeting nirS, nirK and nosZ genes for community surveys of denitrifying bacteria with DGGE. FEMS Microbiol Ecol. 2004 Sep 1; 49:401-17.
9. Humbert S, Zopfi J, Tarnawski SE. Abundance of anammox bacteria in different wetland soils. Environ Microbiol Rep. 2012; 4:484-490.
10. Sonthiphand P, Neufeld JD. Evaluating Primers for Profiling Anaerobic Ammonia Oxidizing Bacteria within Freshwater Environments. PLOS ONE. 2013;8:e57242.
11. Foster M, Deardorff M. Open Science Framework (OSF). J Medical Library Association. 2017: 105.
12. Andrews S. FASTQC. A quality control tool for high throughput sequence data. 2010.
13. Bolger AM, Lohse M, Usadel B. Trimmomatic: a flexible trimmer for Illumina sequence data. Bioinformatics. 2014; 30:2114-2120.

14. Bankevich A, Nurk S, Antipov D, Gurevich AA, Dvorkin M, Kulikov AS et al. SPAdes: a new genome assembly algorithm and its applications to single-cell sequencing. J Comput Biol: a journal of computational molecular cell biology. 2012; 19:455-477.
15. Langmead B, Salzberg SL. Fast gapped-read alignment with Bowtie 2. Nature Methods. 2012; 9:357-359.
16. Li H, Handsaker B, Wysoker A, Fennell T, Ruan J, Homer N et al. The Sequence Alignment/Map format and SAMtools. Bioinformatics, 2009;25:2078-9.
17. Eren AM, Esen OC, Quince C, Vineis JH, Morrison HG, Sogin ML et al. Anvi'o: an advanced analysis and visualization platform for 'omics data. Peer J, 2015;3: e1319.
18. Kanehisa M, Furumichi M, Sato Y, Ishiguro-Watanabe M, Tanabe M. KEGG: integrating viruses and cellular organisms. Nucleic Acids Res. 2020;49(D1):D545-D551.
19. von Meijenfeldt FAB, Arkhipova K, Cambuy DD,Coutinho FH, Dutilh BE. Robust taxonomic classification of uncharted microbial sequences and bins with CAT and BAT. Gen Biol. 2019; 20:217.
20. Core Team R. R: A language and environment for statistical computing. R Foundation for Statistical Computing. Vienna, Austria: URL https://www. R-project.
21. Edgar RC. MUSCLE: multiple sequence alignment with high accuracy and high throughput. Nucleic Acids Research. 2004;32:1792-1797.
22. Price MN, Dehal PS, Arkin AP. FastTree 2 – Approximately Maximum-Likelihood Trees for Large Alignments. PLOS ONE, 2010;5: e9490.
23. Letunic I, Bork P. Interactive Tree Of Life (iTOL) v4: recent updates and new developments. Nucleic Acids Research, 2019;47:W256-W259.
24. Thompson JD, Gibson TJ, Plewniak F, Jeanmougin F, Higgins DG. The ClustalX windows interface: flexible strategies for multiple sequence alignment aided by quality analysis tools. Nucleic Acids Research. 1997; 25:4876-4882.
25. Waterhouse AM, Procter JB, Martin DMA, Clamp M, Barton GJ. "Jalview Version 2 - a multiple sequence alignment editor and analysis workbench" Bioinformatics 2009; 25(9):1189-1191.
